# Genome-wide analysis of the omega-3 fatty acid desaturase gene family in *Gossypium*

**DOI:** 10.1186/s12870-014-0312-5

**Published:** 2014-11-18

**Authors:** Olga P Yurchenko, Sunjung Park, Daniel C Ilut, Jay J Inmon, Jon C Millhollon, Zach Liechty, Justin T Page, Matthew A Jenks, Kent D Chapman, Joshua A Udall, Michael A Gore, John M Dyer

**Affiliations:** USDA-ARS, US Arid-Land Agricultural Research Center, 21881 North Cardon Lane, Maricopa, AZ 85138 USA; Department of Biological Sciences, Center for Plant Lipid Research, University of North Texas, Denton, TX 76203 USA; Plant Breeding and Genetics Section, School of Integrative Plant Science, Cornell University, Ithaca, NY 14853 USA; Plant and Wildlife Science Department, Brigham Young University, Provo, UT 84602 USA; Division of Plant and Soil Sciences, West Virginia University, Morgantown, WV 2650 USA

**Keywords:** Chilling tolerance, Cotton, Drought, Fatty acid desaturase, *Gossypium*, Linolenic acid, Omega-3 fatty acid

## Abstract

**Background:**

The majority of commercial cotton varieties planted worldwide are derived from *Gossypium hirsutum*, which is a naturally occurring allotetraploid produced by interspecific hybridization of A- and D-genome diploid progenitor species. While most cotton species are adapted to warm, semi-arid tropical and subtropical regions, and thus perform well in these geographical areas, cotton seedlings are sensitive to cold temperature, which can significantly reduce crop yields. One of the common biochemical responses of plants to cold temperatures is an increase in omega-3 fatty acids, which protects cellular function by maintaining membrane integrity. The purpose of our study was to identify and characterize the omega-3 fatty acid desaturase (FAD) gene family in *G. hirsutum,* with an emphasis on identifying omega-3 FADs involved in cold temperature adaptation.

**Results:**

Eleven omega-3 FAD genes were identified in *G. hirsutum*, and characterization of the gene family in extant A and D diploid species (*G. herbaceum* and *G. raimondii*, respectively) allowed for unambiguous genome assignment of all homoeologs in tetraploid *G. hirsutum*. The omega-3 FAD family of cotton includes five distinct genes, two of which encode endoplasmic reticulum-type enzymes (*FAD3-1* and *FAD3-2*) and three that encode chloroplast-type enzymes (*FAD7/8-1*, *FAD7/8-2*, and *FAD7/8-3*). The *FAD3-2* gene was duplicated in the A genome progenitor species after the evolutionary split from the D progenitor, but before the interspecific hybridization event that gave rise to modern tetraploid cotton. RNA-seq analysis revealed conserved, gene-specific expression patterns in various organs and cell types and semi-quantitative RT-PCR further revealed that *FAD7/8-1* was specifically induced during cold temperature treatment of *G. hirsutum* seedlings.

**Conclusions:**

The omega-3 FAD gene family in cotton was characterized at the genome-wide level in three species, showing relatively ancient establishment of the gene family prior to the split of A and D diploid progenitor species. The FAD genes are differentially expressed in various organs and cell types, including fiber, and expression of the *FAD7/8-1* gene was induced by cold temperature. Collectively, these data define the genetic and functional genomic properties of this important gene family in cotton and provide a foundation for future efforts to improve cotton abiotic stress tolerance through molecular breeding approaches.

**Electronic supplementary material:**

The online version of this article (doi:10.1186/s12870-014-0312-5) contains supplementary material, which is available to authorized users.

## Background

Cotton is an important crop worldwide, providing the majority of fiber to the textile industry and a significant amount of oilseed for food, feed, and biofuel purposes. The most commonly grown cotton species for commercial production is *Gossypium hirsutum*, an allotetraploid species with a remarkable evolutionary history. The cotton genus (*Gossypium*) originated approximately 12 million years ago (MYA) [1] and underwent rapid radiation and adaptation to many arid or seasonally arid tropical or subtropical regions of the world [2,3]. Despite a wide range of morphological phenotypes, including trees and bushes, cytogenetic and karyotyping analyses revealed that the majority of plants can be categorized as having 1 of 8 distinct types of diploid genomes (n = 13) [3]. The A, B, E, and F genome-containing plants are found in Africa and Arabia, the C, G, and K genomes are common to Australian plants, and the D genome-containing species are found in Mesoamerica. *G. hirsutum* is an AD tetraploid also found predominantly in Mesoamerica, which suggests that this species arose by trans-oceanic dispersal of A-type seed from Africa, followed by chance interspecific hybridization with a D-containing progenitor species in the New World [3,4]. Molecular systematics studies suggest that the A and D diploid species evolved separately for approximately 5–10 million years before being reunited in the same nucleus approximately 1–2 MYA [5]. *G. hirsutum* (the source of upland cotton) was subsequently domesticated for fiber production in the last few thousand years in the New World, and as such, is an interesting model system not only for use in the study of genome evolution, but also for studying the role of polyploidy in crop development and domestication [6].

Given that *G. hirsutum* is native to the tropics and subtropics, it is adapted to the warm temperatures of arid and semi-arid climates [7,8]. In the US, upland cotton is planted at various times throughout the year and the beginning and end of the growing seasons often include sub-optimal growth temperatures and environmental conditions. For instance, heat and drought can cause significant reductions in crop yield during the latter parts of the growing season [9,10]. Exposure of cotton to sudden episodes of cold temperature during the early parts of the growing season, moreover, can cause significant damage to cotton seedlings and the plants may not fully recover [11-15]. Development of upland cotton varieties with improved tolerance to low temperature stress could thus improve the agronomic performance of the crop and thereby significantly impact the cotton industry [12,14].

The adaptation of plants to low temperature is a complex biological process that involves changes in expression of many different genes and alteration in many different metabolites [16-19]. One of the common biochemical responses in plants to cold temperature is an increase in relative content of polyunsaturated fatty acids (PUFAs) [20-23]. Polyunsaturated fatty acids have a lower melting temperature than saturated and monounsaturated fatty acids, and their increased accumulation is thought to help maintain membrane fluidity and cellular integrity at cold temperatures [24]. For instance, cold temperature treatment of cotton seedlings has been shown to induce the accumulation of PUFAs [15,25], and inclusion of an inhibitor of PUFA biosynthesis during the treatment rendered the seedlings more susceptible to cold temperature damage [15]. By contrast, warm temperatures were inversely associated with PUFA content and changed during leaf expansion, and this impacted photosynthetic performance of cotton plants in the field [26]. Thus, gaining a better understanding of the genes that regulate PUFA production in cotton represents a first step in improving cold and thermotolerance in upland cotton germplasm.

The metabolic pathways for PUFA production in plants are generally well understood and have been elucidated primarily by studying various *fatty acid desaturase,* or *fad* mutants, of *Arabidopsis* that are blocked at various steps of lipid metabolism [27]. Briefly, fatty acid biosynthesis occurs in the plastids of plant cells, with a successive concatenation of 2 carbon units resulting in production of the 16- or 18-carbon long fatty acids that predominate in cellular membranes. A soluble fatty acid desaturase is present in the plastid stroma for conversion of 18:0 into 18:1, where the number before the colon represents the total number of carbons in the fatty acid chain and the number after the colon indicates the number of double bonds. The 18:1 fatty acid is subsequently available for further desaturation by one of two parallel pathways operating in either the plastid or endoplasmic reticulum (ER). For instance, 18:1 may be converted to 18:2 in plastids by a membrane-bound fatty acid desaturase called FAD6, or the 18:1 may be exported from the plastids to the ER for conversion to 18:2 by a structurally related enzyme called FAD2. The FAD2 and FAD6 enzymes are similar at the polypeptide sequence level, with the exception that the FAD6 protein contains a longer N-terminal sequence that is characteristic of a chloroplast transit peptide. In a similar fashion, 18:2 may be converted into 18:3 in plastids by the FAD7 or FAD8 enzymes, which are encoded by two closely related genes in *Arabidopsis*, or can be exported to the ER for conversion to 18:3 by the FAD3 enzyme. This latter group of enzymes (FAD7/FAD8 and FAD3) are referred to as omega-3 fatty acid desaturases, since they introduce a double bond at the omega-3 position of the fatty acid structure. Thus the FAD6 and FAD2 enzymes, which produce 18:2, and the FAD7/FAD8 and FAD3 enzymes, which produce 18:3, all play central roles in production of the PUFAs that are present in all plant species.

Knowledge of the FAD genes encoding these enzymes has permitted more detailed analyses of the role of these genes, and their fatty acid products, in plant lipid metabolism and abiotic stress response. For instance, omega-3 fatty acids are known to increase in plants in response to both drought [28,29] and cold temperature [20-23], and over-expression of omega-3 desaturases in various transgenic plants has been shown to improve both drought and chilling tolerance [30-35]. The ER-localized desaturases FAD2 and FAD3 are also involved in production of PUFA components of seed oils [27], and given the importance of these fatty acids to human nutrition, and to determining stability of oils during cooking or other food applications, molecular markers for these genes have been developed for evaluating germplasm and identifying oilseed varieties with improved oil compositions [36-39].

Given the prominent role of PUFAs in chilling and drought adaptation of plants, and the susceptibility of cotton seedlings to both of these environmental conditions, we sought to identify and characterize the genes involved in PUFA synthesis in cotton. Since several *FAD2* genes have been previously reported and characterized in cotton [40-46], we chose instead to focus on the analysis of the omega-3 FAD gene family, of which no members have been previously studied. Here we describe the complete omega-3 gene family in both tetraploid *G. hirsutum* as well as extant A and D diploid progenitor species (*G. herbaceum* and *G. raimondii*, respectively), which allowed clear assignment of all homoeologous genes. We also describe organ and cell-type specific gene expression patterns, and identify a single *FAD7/FAD8*-type gene that is inducible by both drought as well as cold-temperature exposure of cotton seedlings. Collectively, these data define the content and functional genomic properties of this important gene family in commercial upland cotton.

## Results and discussion

### Identification and phylogenetic analysis of the omega-3 FAD gene family in cotton

The omega-3 FAD-type genes in *G. hirsutum* (AD_1_ allotetraploid), *G. herbaceum* (A_1_ diploid), and *G. raimondii* (D_5_ diploid) were cloned and sequenced using a combination of database mining, degenerate primer-based PCR screening, genome resequencing, and gene-specific PCR-based cloning, as described in the Methods. All cloning, DNA sequencing, and RT-PCR primer sequences are provided in Additional files 1, 2, and 3, respectively. During the cloning process, the genome sequence of *G. raimondii* (D_5_) was released [47], which confirmed the identity of omega-3 genes we had identified in this organism. The perfect match between our cloned gene sequences and the genes in the genome database provided a useful check for the fidelity of the cloning process employed here. More recently, a draft of the genome sequence of *G. arboreum* (A_2_) was released [48], which will enable future studies aimed at comparing gene sequences between A genome-containing species.

**Figure 1:**
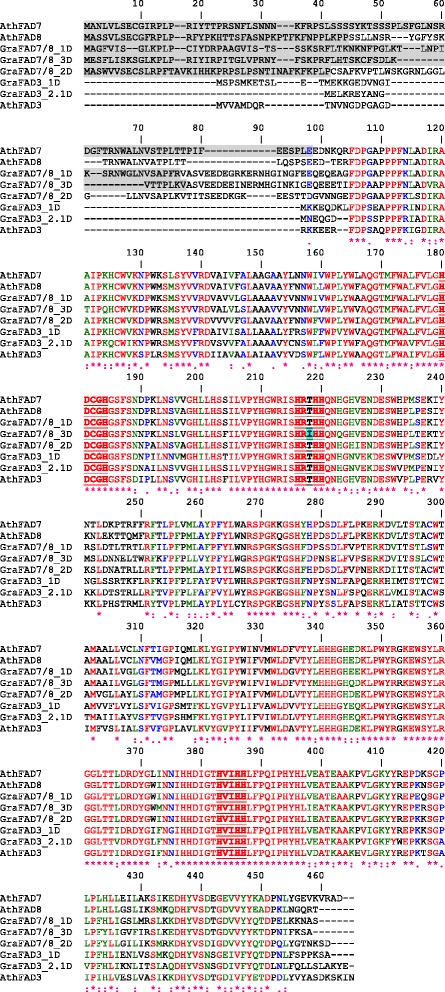
**Alignment of encoded omega-3 FAD polypeptide sequences from** ***G. raimondii*** **(Gra) and** ***Arabidopsis thaliana*** **(Ath).** Polypeptide sequences were aligned using the ClustalW algorithm with default parameters (npsa-pbil.ibcp.fr; [52]). Each polypeptide sequence was evaluated using ChloroP (www.cbs.dtu.dk/services/ChloroP/; [53]) to identify putative chloroplast transit peptides, which are highlighted grey. Identical amino acids are highlighted in red, and the three conserved “histidine boxes” known to be involved in binding two iron atoms at the active site [49] are bolded and underlined. Note the substitution of a threonine residue with isoleucine in the FAD7/8-3 sequence of the second histidine box, which is highlighted blue.

Five distinct omega-3 FAD-type genes were identified, and all of the genes were present in each of the three cotton species studied, which allowed for unambiguous assignment of each homoeolog in *G. hirsutum* (Table 1; see Additional file 4 for GenBank accession numbers and Additional files 5, 6, 7, 8 and 9 for gene alignments). Two of the genes encode FAD3-type enzymes localized in the ER (*FAD3-1* and *FAD3-2*) and three genes encode FAD7/8-type enzymes in the chloroplast (*FAD7/8-1*, *FAD7/8-2*, *FAD7/8-3*) (Figure 1; only the encoded polypeptide sequences from *G. raimondii* are shown for clarity). The latter group of polypeptides contained longer N-terminal sequences predicted to serve as chloroplast targeting peptides (Figure 1). All of the omega-3 FADs shared conserved regions of polypeptide sequence, including three “histidine boxes” that are involved in binding two iron atoms at the enzyme active site (Figure 1; [49]). Notably, the enzyme encoded by *FAD7/8-3* harbored a threonine to isoleucine substitution within the second histidine box (Figure 1), which is typically not observed in FAD7/8-type sequences (Figure 1 and [50]), and this substitution was detected in all *FAD7/8-3* sequences in the three cotton species (data not shown). Given the highly conserved nature of the histidine box sequences in various FAD7/8-type enzymes [50], and that alterations to these regions are known to disrupt or alter enzyme activity [51], these data suggest that the *FAD7/8-3* gene of cotton might encode an enzyme with reduced or altered enzyme activity.

**Table 1:** **Summary of omega-3 FAD genes cloned from cotton**

**Omega-3**				
**FAD gene**	***G. herbaceum***	***G. raimondii***	***G. hirsutum***	**Type**
*FAD3-1*	GheFAD3-1A*	*GraFAD3-1D*	*GhiFAD3-1A, GhiFAD3-1D*	ER
*FAD3-2*	*GheFAD3-2.1A*	*GraFAD3-2.1D*	*GhiFAD3-2.1A*, *GhiFAD3-2.1D*	ER
	*GheFAD3-2.2A*	---	*GhiFAD3-2.2A*	---
			---	
*FAD7/8-1*	*GheFAD7/8-1A*	*GraFAD7/8-1D*	*GhiFAD7/8-1A*, *GhiFAD7/8-1D*	Chloroplast
*FAD7/8-2*	*GheFAD7/8-2A*	*GraFAD7/8-2D*	*GhiFAD7/8-2A*, *GhiFAD7/8-2D*	Chloroplast
*FAD7/8-3*	*GheFAD7/8-3A*	*GraFAD7/8-3D*	*GhiFAD7/8-3A*, *GhiFAD7/8-3D*	Chloroplast

*Gene nomenclature includes the first three letters of the plant genus and species, followed by the gene name, and ending with the genome designation (A for *G. herbaceum* or the A subgenome of *G. hirsutum*, or D for *G. raimondii* or the D subgenome of *G. hirsutum*). The *FAD3-2* gene is duplicated in both *G. herbaceum* and *G. hirsutum*, and the paralogs are designated *FAD3-2.1* and *FAD3-2.2*. The coding sequence of *FAD3-2.2* contains multiple in-frame stop codons and a frame-shift mutation and thus is likely a pseudogene. The single *FAD3-2* gene within *G. raimondii* is designated *FAD3-2.1* for clarity to indicate that it is more similar to the *FAD3-2.1* sequence in the A genome-containing species. GenBank accession numbers are provided in Additional file 4.

**Figure 2:**
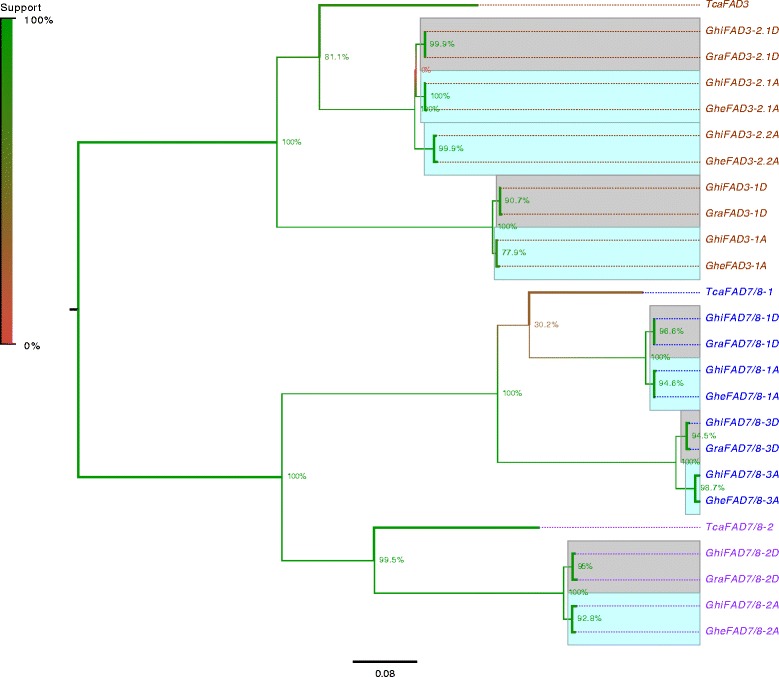
**Phylogenetic tree of omega-3 FAD genes from** ***G. raimondii*** **(Gra),** ***G. herbaceum*** **(Ghe),** ***G. hirsutum*** **(Ghi)** ***,*** **and** ***T. cacao*** **(Tca).** Gene name abbreviations correspond to those in Table 1. Branches are color-coded based on phylogenetic support, and support for individual nodes is indicated on the figure. Taxon names are color-coded based on the three major monophyletic groups: Clade 1 (brown), Clade 2 (blue), and Clade 3 (purple). Cotton A and D genome genes are highlighted in cyan and grey respectively, and dotted lines are used to indicate the terminal branches corresponding to the right-justified labels.

To gain insight to the evolution and function of the omega-3 FAD gene family in cotton, the omega-3 sequences in the three species were compared with the sequences of *Theobroma cacao*, which is a close relative of cotton in the *Malvaceae* family and whose genome has been sequenced [54]. Phylogenetic analysis revealed that the omega-3 FADs in these species separated into three well defined monophyletic groups, each of them containing one cacao and several cotton genes (Figure 2). The establishment of these three groups thus predates the divergence of cotton and cacao approximately 60 MYA [47]. In cotton, the gene family underwent further expansion after divergence from *T. cacao* but before divergence of the A and D genome species circa 6–7 MYA [55], with duplicated gene pairs observed for *FAD3*-type (*FAD3-1* and *FAD3-2.1*) and *FAD7/8*-type (*FAD7/8-1* and *FAD7/8-3*) genes in two of the three monophyletic groups (Figure 2; Table 1). These duplications are consistent with the genome duplication events that occurred in the cotton lineage shortly after its divergence from cacao [47]. Moreover, the *FAD3-2.1* gene underwent further duplication in the A genome species (*G. herbaceum*), but not in the D genome species (*G. raimondii*), and this further duplication persists in tetraploid *G. hirsutum*. These data indicate that the latter duplication event happened after the split of the diploid progenitor species, but before the interspecific hybridization event that gave rise to tetraploid *G. hirsutum* circa 1–2 MYA [4]. The *FAD3-2.2* gene is likely a pseudogene, because the coding sequence contains several in-frame stop codons and a frame-shift mutation that are present in both *G. herbaceum* and *G. hirsutum* sequences (Additional file 6). Taken together, these data reveal that the omega-3 FAD gene family underwent rapid expansion during cotton speciation, with additional elaboration in A genome species prior to interspecific hybridization.

### RNA-seq analysis of gene expression patterns

**Figure 3:**
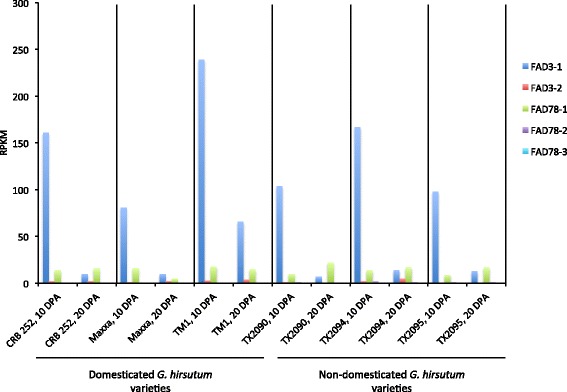
**Expression of omega-3 FAD genes in developing cotton fibers.** Cotton fibers were harvested at 10 and 20 DPA, which represents primary and secondary cell wall synthesis, respectively, and RNA-seq analysis was performed as described [56]. Transcripts were quantified as “reads per kilobase per million mapped reads” (RPKM). For simplicity, data for A and D homoeologous sequences were combined. Plant varieties are listed along the bottom and include Coker315 and TM1, which represent domesticated cotton *G. hirsutum* varieties, and TX2090 and TX2094, which are wild *G. hirsutum* varieties.

To gain insight to the function of the omega-3 FAD genes, the expression patterns in various cotton organs, cell types and treatments were evaluated based on RNA-seq experiments. A recent transcriptomic study of developing cotton fibers in wild and domesticated *G. hirsutum* lines revealed that the domestication process resulted in massive reprogramming of fiber gene expression, with over 5,000 genes showing significant changes in expression between wild and domesticated species [56]. Wild cotton fibers are short and brown, while domesticated fibers are longer and white. Two developmental stages were studied, including 10 days post anthesis (DPA), which represents primary cell wall growth, and 20 DPA, representing the transition to secondary cell wall synthesis [56]. Analysis of RNA-seq data for the omega-3 FAD gene family revealed that the *FAD3-1* gene was predominantly expressed during primary cell wall synthesis, and was reduced during secondary wall synthesis (Figure 3). All other omega-3 FAD genes were expressed at very low levels. This pattern was consistently observed in both wild and domesticated *G. hirsutum* varieties (Figure 3), suggesting that *FAD3-1* expression is involved in a shared, and not domestication-specific, aspect of fiber production. Notably, linolenic acid is the most abundant fatty acid in elongating cotton fibers [57], and a separate study of gene expression in 1 vs. 7 DPA fibers in *G. hirsutum* showed strong induction of a *FAD3*-type gene during primary cell wall synthesis [57]. Comparison of the gene fragment identified in that study with the sequences described here showed that the gene fragment corresponded to the *FAD3-1D* homoeolog of *G. hirsutum* (data not shown). Taken together, these data suggest that the *FAD3-1* gene plays an important role in directing synthesis of high levels of omega-3 fatty acids present in elongating cotton fibers.

**Figure 4:**
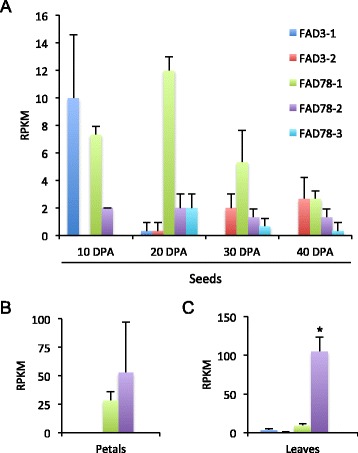
**Expression of omega-3 FAD genes in** ***G. hirsutum*** **seeds, petals and leaves. (A)** Developing cottonseeds were harvested from *G. hirsutum* plants at the indicated times, then RNA-seq analysis was performed as described. Transcripts were quantified as “reads per kilobase per million mapped reads” (RPKM). For simplicity, data for A and D homoeologous sequences were combined. RNA-seq was also performed on cotton petals **(B)** as well as cotton leaves **(C)**, as described [59,60]. Values represent average and standard deviation of three biological replicates. For data presented in panels **(B)** and **(C)**, student’s t-test was used for comparison of FAD7/8-1 to FAD7/8-2, and * denotes *p* <0.05.

Analysis of transcript levels in adjacent, developing seed tissues of domesticated *G. hirsutum* showed a very different gene expression profile than fibers, with low levels of all omega-3 gene family members observed at each time point (Figure 4A). This likely explains the very low level of linolenic acid found in cottonseed oil, which accounts for ~0.2% of seed oil fatty acid composition [58]. Analysis of transcripts in petals, however, showed relatively high levels of expression for both *FAD7/8-1* and *FAD7/8-2* (Figure 4B). Analysis of cotton leaves showed a somewhat similar pattern, but *FAD7/8-1* levels were reduced (Figure 4C). Notably, similar gene expression patterns were detected in fibers, seeds, petals and leaves of other cotton varieties and species, suggesting that the mechanisms of omega-3 FAD gene regulation were anciently established (Additional file 10). Taken together, these data reveal conserved, and differential gene expression patterns in various tissues and organs in cotton.

**Figure 5:**
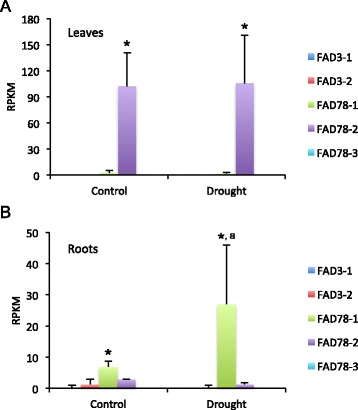
**Expression of omega-3 FAD genes in drought-treated** ***G. hirsutum*** **plants.** The *G. hirsutum* cultivar Siokra L-23 was subjected to drought treatment, then gene expression in cotton leaves **(A)** or roots **(B)** was analyzed by RNA-seq analysis, as described [61]. Transcripts were quantified as “reads per kilobase per million mapped reads” (RPKM). For simplicity, data for A and D homoeologous sequences were combined. Values represent average and standard deviation of three biological replicates. Student’s t-test was used for comparison of FAD7/8-1 to FAD7/8-2, and * denotes *p* <0.05. A comparison was also made between FAD7/8-1 in control and drought treated plants, and the “a” denotes *p* <0.10.

RNA-seq analysis was also performed on cotton plants subjected to drought treatment. The *G. hirsutum* cultivar Siokra L-23 was used for this analysis since it was previously selected for enhanced water-deficit tolerance [61]. Examination of omega-3 FAD transcript levels in control and drought treated cotton leaves confirmed that *FAD7/8-2* was predominantly expressed in leaves, and furthermore that expression of this gene did not change appreciably in response to drought (Figure 5A). Analysis of gene expression in root tissues, however, revealed that the *FAD7/8-1* gene was predominantly expressed, and expression was moderately induced by drought treatment (Figure 5B).

Taken together, these data define organ and cell-type specific gene expression patterns for various members of the omega-3 fatty acid desaturase gene family in *G. hirsutum*, with *FAD3-1* expressed predominantly in fibers, *FAD7/8-2* in leaves, and *FAD7/8-1* induced by drought treatment in cotton roots.

### *FAD7/8-1* expression is induced in cotton seedlings in response to cold temperature

To investigate gene expression patterns in cold-treated *G. hirsutum* seedlings, we first developed gene-specific PCR primers capable of distinguishing each omega-3 FAD homoeolog. We chose to develop PCR-based strategies rather than RNA-seq for monitoring gene expression since the PCR primers developed herein can be used also for future candidate gene association mapping studies. The goal of such mapping studies is to test whether sequence variants (e.g., single-nucleotide polymorphisms, SNPs) at candidate genes are statistically associated with a particular trait (e.g., chilling tolerance) in a panel of diverse lines [62,63]. To develop homoeolog-specific primers, we first aligned the respective omega-3 FAD genes to identify SNPs and insertions-deletions (indels) that were specific to each gene (Additional files 5, 6, 7, 8 and 9). Our general strategy for designing primers was that each primer pair should amplify a fragment of approximately 500 bp from mRNA, and the 3′-most nucleotide of each primer should be unique to each homoeolog. The specificity of each primer set was tested and optimized using gradient PCR annealing conditions and plasmid DNA templates containing either the target homoeolog, or the most closely related sequence. In some cases, the primers amplified both homoeologs and needed redesigning for improved specificity. The final sets of primers capable of distinguishing each homoeolog are listed in Additional file 3. Primer optimization experiments for *FAD3*-type genes are presented in Additional file 11, and *FAD7/8*-type genes are shown in Additional file 12.

**Figure 6:**
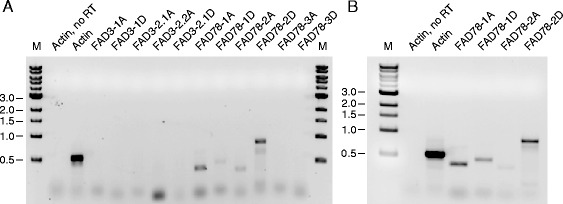
**Detection of omega-3 FAD transcripts in** ***G. hirsutum*** **leaves using semi-quantitative RT-PCR.** The *G. hirsutum* cultivar TM-1 was grown in a growth chamber at 30°C with 12 h light/12 h dark cycles, then the first true leaves were collected on the 13^th^ day after germination **(A)**, or plants were grown until the 7^th^ fully expanded leaf could be collected **(B)**. Leaf samples were immediately frozen in liquid nitrogen and stored prior to use. RT-PCR analysis of cDNA was performed as described in the Methods, and samples were analyzed by DNA gel electrophoresis and ethidium bromide staining. The target gene of each PCR reaction is listed along the top, and Actin reactions without reverse transcription were included as a negative control. M – DNA ladder, with positions of markers (in kbp) listed on the left. Note the similar expression of *FAD7/8-1* and *FAD7/8-2* genes in the 13-day-old leaves **(A)** and the 7^th^ leaf **(B)**.

Semi-quantitative RT-PCR analysis of transcript levels in fully expanded cotyledons (Additional file 12B) and 13-day-old leaves of seedlings (Figure 6A) showed that the *FAD7/8-1* and *FAD7/8-2* genes were each expressed, and homoeologous transcripts for each gene could be detected. Notably, the sizes of all RT-PCR products corresponded to the sizes expected from amplification of the respective homoeologous cDNAs (Additional files 11 and 12), and not from genomic DNA, and no PCR products were detected in Actin control reactions that did not include the reverse transcription step (Figure 6). The presence of relatively similar levels of *FAD7/8-1* and *FAD7/8-2* RT-PCR products in cotyledons and leaves, however, was somewhat unexpected, given the relatively higher level of *FAD7/8-2* expression detected by RNA-seq analysis of cotton leaves (Figure 4C). Since the latter experiments were performed on the 7^th^ true leaf [59], we also measured omega-3 FAD transcript levels in leaves of this age, and observed a similar expression pattern as in the younger leaves and cotyledons (Figure 6B). While the reasons for the differences in relative expression levels measured by the two techniques are currently unknown, the results of the two approaches are at least consistent in that both reveal measurable levels of expression for both *FAD7/8-1* and *FAD7/8-2* genes. Possible explanations for the differences in gene expression include sensitivities of the two techniques employed (such as differences in primer amplification efficiencies that are not accounted for during semi-quantitative RT-PCR) and/or differences in plant growth conditions (chamber vs. greenhouse).

**Figure 7:**
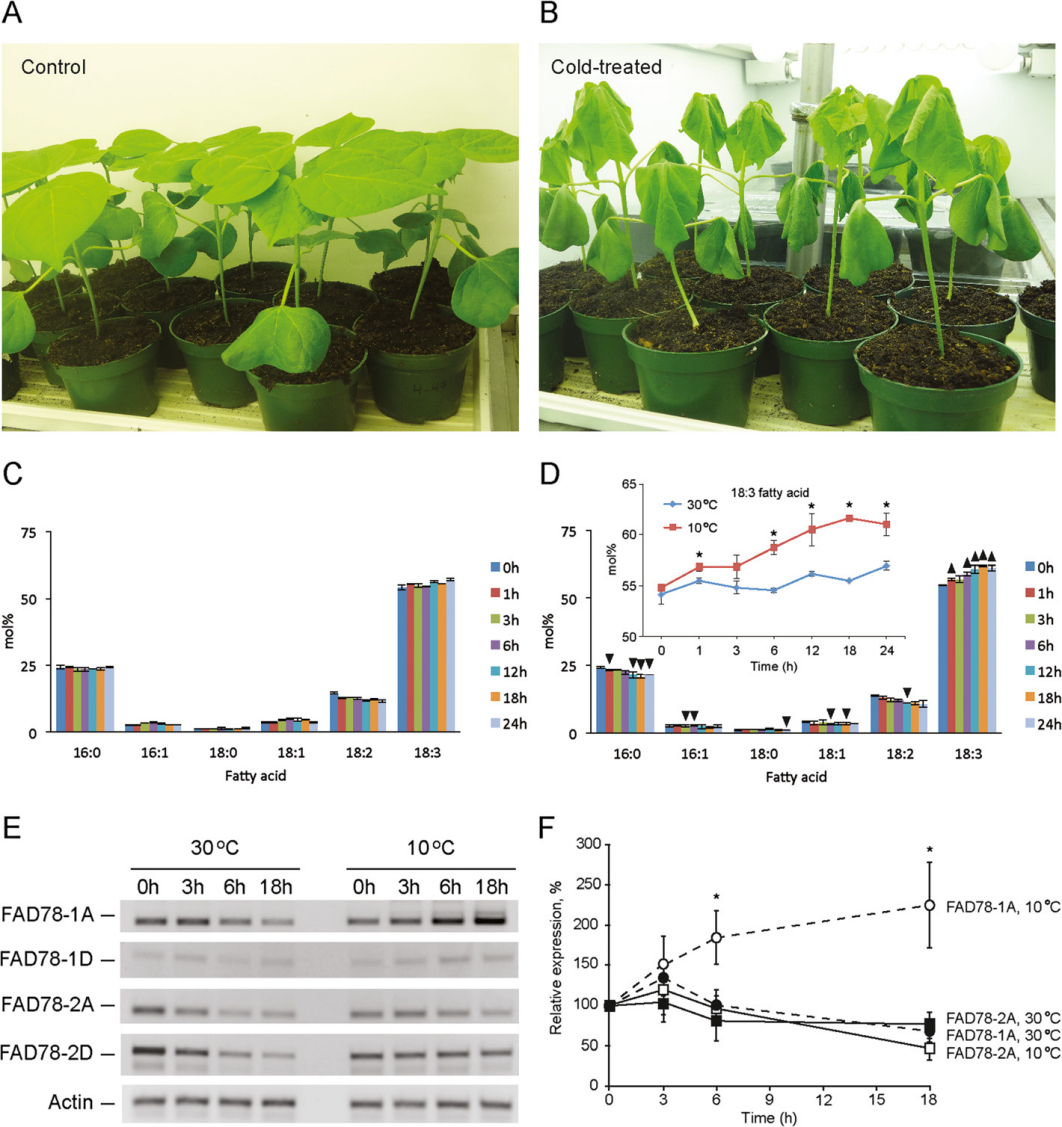
**Cold-temperature treatment of** ***G. hirsutum*** **seedlings.** Plants were grown in a growth chamber at 30°C for 13 days, then a portion of the plants were transferred at the beginning of the 14^th^ day to a similar chamber held at 10°C and the first true leaves were sampled for 24 hours for fatty acid and gene expression analysis. Images of plants grown at either 30°C (Control) **(A)** or 10°C (Cold-treated) **(B)** for 6 hours showed significant wilting of plant leaves at 10°C **(B)**. Fatty acid composition of control **(C)** or cold-treated **(D)** plants was determined over a 24-hour period. Values represent the average and standard deviation of three biological replicates. Student’s t-test was used to compare percentages of each fatty acid between control and cold-treated samples. Solid, upward pointing arrowheads in panel **(D)** represent a statistically significant increase in fatty acid composition (*p* <0.05) in response to cold, while down arrowheads represent a decrease in response to cold (*p* <0.05). The inset in panel **(D)** shows a line graph of 18:3 fatty acid content at either 30 or 10°C (*, *p* <0.05). **(E)** Representative semi-quantitative RT-PCR analysis showing prominent cold-induced expression of the *FAD7/8-1* gene at 10°C (right side) compared to the 30°C control samples (left side). **(F)** Quantitative analysis of band intensities in panel **(E)** relative to t = 0 for the same temperature treatment revealed a statistically significant induction of FAD7/8-1A expression at 10°C (open circles, dashed line) in comparison to 30°C (closed circles, dashed line), while FAD7/8-2A was not induced by cold temperature (open squares, solid line) in comparison to the control (closed squares, solid line). Values represent the average and standard deviation of three biological replicates, and student’s t-test was used for comparison of the same gene at different temperatures. * denotes *p* <0.05.

To determine whether any of the omega-3 fatty acid desaturase genes were induced in *G. hirsutum* seedlings in response to cold temperature, cotton seeds were germinated in pots in a growth chamber at 30°C with a 12 h/12 h day/night cycle and seedlings allowed to establish for 13 days. On the morning of the 14^th^ day, a portion of the plants were moved to a different growth chamber held at 10°C, then leaf samples were collected from both control and cold-treated plants at various time points and immediately frozen in liquid nitrogen prior to use. As shown in Figure 7A and B, cotton seedlings exhibited pronounced wilting after just 6 hours of cold temperature exposure, which is similar to what had been observed previously [13]. Biochemical analysis of leaf fatty acid composition during cold temperature adaptation showed an increase in omega-3 fatty acids (18:3) and decrease in omega-6 fatty acids (18:2) in cold treated plants (Figure 7C and D), which is consistent with enhanced omega-3 FAD enzyme activity [25]. Measurement of omega-3 FAD gene expression patterns in control and cold-treated plants using RT-PCR revealed that the *FAD7/8-1* gene expression increased significantly at 6 and 18 hours (Figure 7E and F), which generally correlated with the temporal increase in 18:3 fatty acids (compare Figure 7D and F). While *FAD7/8-2* was not as dramatically induced, the expression level did appear to be somewhat altered by cold temperature treatment in comparison to the control. Notably, the patterns of gene expression for *FAD7/8-1* and *FAD7/8-2* were observed for both A and D subgenomic copies, suggesting relatively ancient, predominant establishment of *FAD7/8-1* as a cold-responsive gene in cotton.

## Conclusions

Five omega-3 FAD-type genes were identified in cotton, two of which encode ER-localized enzymes (*FAD3-1* and *FAD3-2*) and three that encode chloroplast-type enzymes (*FAD7/8-1*, *FAD7/8-2* and *FAD7/8-3*) (Table 1; Figure 1). Phylogenetic analysis revealed that the genes could be grouped into three major clades (Figure 2), the first of which contained all *FAD3*-type genes. Functional analysis revealed that the *FAD3-1* gene is predominantly expressed in elongating cotton fibers (Figure 3) and likely contributes to the synthesis of linolenic acid, the most abundant fatty acid in fibers. The *FAD3-2* gene is expressed at a relatively low level in all conditions examined here, and is duplicated in the A genome of *G. herbaceum* and A subgenome of *G. hirsutum*. This latter paralog also contains several in-frame stop codons (Additional file 6). Given the low expression of *FAD3-2* compared to *FAD3-1*, it seems likely that *FAD3-1* plays a more dominant role in production of linolenic acid in the ER of cotton cells. Clade 2 contains the *FAD7/8-1* and *FAD7/8-3* genes (Figure 2), and RNA-seq and semi-quantitative RT-PCR showed that *FAD7/8-1* is induced by abiotic stress, including drought treatment in roots (Figure 5B), and cold treatment in cotton leaves (Figure 7E and F). The *FAD7/8-3* gene is expressed at a relatively low level in all experiments described here and includes a substitution mutation in a highly conserved region of the encoded polypeptide sequence (Figure 1). The third clade includes the *FAD7/8-2* gene, which likely serves more of a housekeeping role for production of 18:3 in cotyledons (Additional file 12B), leaves (Figure 4 and Additional file 10D), and petals (Figure 4 and Additional file 10C). Unlike *FAD7/8-1*, the *FAD7/8-2* gene did not show pronounced induction by abiotic stress in response to either drought (Figure 5A) or cold temperature treatment (Figure 7E and F). Notably, the *Arabidopsis FAD7* and *FAD8* genes also show differential response to chilling treatment, with *FAD7* expression unaffected by cold temperature [64] while *FAD8* expression is induced at low temperature [65]. Taken together, these data define the evolutionary and functional properties of the omega-3 FAD gene family in cotton and identify specific members of the gene family associated with fiber biogenesis and abiotic stress response.

## Methods

### Gene cloning and annotation

For the initial search of omega-3 FAD genes in cotton, we employed several different approaches including i) BLAST analysis of extant *Gossypium* sequences in various genome databases (e.g., NCBI, CottonDB, Phytozome) using *Arabidopsis thaliana* omega-3 desaturases as queries; ii) PCR-based screening of cotton genomic DNA and cDNA libraries using “degenerate” PCR primers corresponding to conserved regions of omega-3 fatty acid polypeptide sequences; and iii) PCR amplification using gene-specific primers (see Additional file 1 for primer sequences). Genomic DNA from *G. herbaceum*, *G. raimondii*, *G. hirsutum*, and *G. barbadense* was used as templates in PCR reactions. Additional insight to the omega-3 FAD gene family was obtained with the release of the genome sequence for the diploid progenitor *G. raimondii* [47].

Our preliminary analysis identified five different omega-3 desaturase genes in cotton, including two genes encoding putative endoplasmic reticulum-localized enzyme (*FAD3-1* and *FAD3-2*), and three genes encoding putative chloroplast-localized enzymes (*FAD7/8-1*, *FAD7/8-2*, *FAD7/8-3*). Each of the five genes was subsequently cloned and sequenced from two progenitor-type cotton species, *G. herbaceum* (A genome; PI 175456) and *G. raimondii* (D genome; PI 530901), as well as from modern upland cotton, *G. hirsutum* TM1 (AD tetraploid; PI 662944 MAP; [66]). To ensure fidelity of cloned gene sequences, the following strategy was employed. Gene-specific PCR primers were designed to hybridize in the 5′ and 3′ UTR regions near the start and stop codons, respectively, and used in PCR reactions containing genomic DNA isolated from a single plant from each species. The PCR reaction was divided into three aliquots that were each subjected to PCR amplification using a gradient of annealing temperatures, and extension times appropriate for each gene. The high fidelity polymerase “Phusion” (New England Biolabs, Ipswich, MA) was used for amplification. PCR products were resolved on DNA gels and bands of expected size were extracted and purified using the GeneClean kit (MP Biomedicals, Santa Ana, CA) and ligated into appropriate plasmid vectors. In some cases, blunt-ended PCR products were subcloned into pZero-Blunt (Life Technologies, Carlsbad, CA), while in other cases, unique restrictions sites were added to the 5’ and 3’ ends of gene-specific primers to allow for directional subcloning into pUC19.

Ten individual plasmids derived from each of the three initial PCR reactions for a single gene (30 plasmids total), were subject to DNA sequencing (Retrogen, Inc., San Diego, CA), with DNA sequences determined in both the forward and reverse directions (see Additional file 2 for sequencing primers). Full-length gene sequences were assembled using the ContigExpress module of Vector NTI (v 11.0; Life Technologies, Grand Island, NY). The sequences of all thirty plasmids representing a single gene target were aligned to help identify sequencing artifacts, PCR-based artifacts, and gene sequences resulting from PCR-based recombination. The latter artifact is quite common when amplifying a gene from a polyploid plant, such as *G. hirsutum* [67], and involves essentially random crossing over of homoeologous sequence templates during PCR amplification. Knowledge of the omega-3 FAD gene sequences from the diploid progenitor species was essential for helping to determine the homoeologous gene sequences in tetraploid *G. hirsutum*. Intron/exon assignments were determined by aligning the genomic sequences with cotton FAD cDNAs or ESTs, if available, or predicting splice sites using algorithms available at www.softberry.com and comparison to well characterized sequences of *Arabidopsis* omega-3 desaturases. All of the gene sequences, as well as putative mRNA sequences, from *G. herbaceum*, *G. raimondii*, and *G. hirsutum* were deposited in GenBank and accession numbers are provided in Additional file 4.

### Phylogenetic analysis

The 22 full-length omega-3 FAD gene sequences identified in this study, as well as 3 homologous sequences from the *T. cacao* genome (Additional file 4) were aligned with T-Coffee (v10.0; [68]) using default parameters. The multiple sequence alignment was subsequently cleaned using Gblocks [69] to remove poorly aligned and predominantly gap-containing regions, using the following parameters: a minimum of 15 sequences were required for a conserved position and a flank position, no limit was placed on the number of contiguous nonconserved positions, at least two nucleotides were required for a conserved block, and gaps were allowed in at most 14 taxa at a given position. An unrooted phylogenetic tree was generated using the maximum likelihood method implemented in the online version of the PhyML program (http://www.atgc-montpellier.fr/phyml v3.0; [70]) using a BioNJ [71] initial tree and a GTR + gamma nucleotide substitution model with parameters inferred from the data. Branch support was estimated using the aLRT method [72]. The tree was visualized and annotated using FigTree (v1.4; http://tree.bio.ed.ac.uk/software/figtree). For visualization purposes, the tree was mid-point rooted.

### Evaluation of gene expression using RNA-seq and semi-quantitative RT-PCR

RNA-seq measurements of gene expression were mined from several cotton RNA-seq studies [56,59,60] that sampled fiber, leaf, and petal tissues. RNA-seq expression levels of the FAD genes in developing seeds were obtained from R. Hovav and J. Wendel (personal communication). RNA-seq data for the drought experiment were obtained from [61]. The omega-3 FAD genes identified in this study were aligned against the D-genome reference sequence [47] using GSNAP [73] in order to identify the corresponding gene models, and RPKM (reads per kilobase per million mapped reads) measurements corresponding to the appropriate gene model were extracted from the published data.

For semi-quantitative RT-PCR, gene-specific primers were designed by aligning the homoeologous sequences of omega-3 desaturases from *G. hirsutum* to identify SNPs and indels that were specific to each gene (see Additional files 5, 6, 7, 8 and 9). The specificity of primer binding was then evaluated using PCR and plasmid templates harboring either the target gene or the closest related homoeolog. Optimal annealing temperatures for each primer pair were determined using gradient annealing PCR conditions. In some cases, amplification was observed using both plasmid templates, at all annealing temperatures, and primers were subsequently re-designed to bind to other gene-specific regions and specificity tested. All final primer sets showed homoeolog-specific amplification from plasmid DNA templates at the indicated optimal annealing temperatures (see Additional files 11 and 12).

To evaluate transcript abundance using RT-PCR, total RNA was extracted from frozen leaf tissue using the Spectrum Plant Total RNA Kit (Sigma-Aldrich, St. Louis, MO) then cDNA was constructed using 1 μg of total RNA and the iScript kit, as described by the manufacturer (Bio-Rad, Hercules, CA). Additional reactions were set up with iScript RT supermix, but without reverse transcriptase, to control for potential genomic DNA contamination. Semi-quantitative PCR was conducted using Extract-N-Amp PCR Ready Mix (Sigma-Aldrich) programmed with 1 μl of cDNA template (corresponding to 50 ng of RNA/cDNA) and each FAD gene primer pair, or 0.1 μl of cDNA template (corresponding to 5 ng of RNA/cDNA) and actin gene primers. These amounts of plasmid DNA template, as well as number of cycles, were determined empirically for each gene to ensure that amplification was within the linear range. The PCR included the following program conditions: initial hold at 98°C for 30 sec, then 30 cycles of denaturation at 95°C for 10 sec, annealing at the appropriate T_m_ for each primer pair (determined as described above) for 30 sec, then extension at 72°C for the period determined for each PCR product. After the 30^th^ cycle, reactions were incubated at 72°C for an additional 5 min to complete extension then held at 4°C, followed by storage at −20°C. Four μl of 6X loading dye was added to the 20 μl PCR reactions, then 10 μl of each sample was analyzed by agarose gel electrophoresis and ethidium bromide staining. Gel images were captured using a FUJI LAS-4000 imaging system and band intensities were quantified using MultiGauge V3.1 software.

### Plant growth conditions and sample collections

Cotton seeds were planted in damp soil in 24×16×16 plastic pots (1 seed per pot), covered with a plastic dome, and placed in a growth chamber programmed with a 12 h/12 h day/night cycle at 30°C, 60% relative humidity, and 2.14 Kfc light intensity. Plants were watered twice a week, and fertilized with 20-20-20 once a week. 13-day-old plants were subjected to chilling treatment (10°C) at the beginning of the 14^th^ light/dark cycle. The control group of plants remained at 30°C for the 14^th^ cycle. The first true leaf was collected at 0, 1, 3, 6, 12, 18 and 24 h after the start of the cold treatment for both treatment and control groups and immediately frozen in liquid nitrogen and stored at −80°C until used. Three biological reps were collected for each time point.

### Lipid extraction and GC/FID analysis

Total lipids were extracted from the leaf tissues using a modified Bligh and Dyer method [74]. Briefly, frozen leaves were placed in a glass tube with 3 ml of preheated (75°C) isopropanol and incubated for 15 min. After the samples cooled to room temperature, 1.5 ml of chloroform and 0.6 ml of water were added. The samples were vigorously shaken, and the lipids were extracted for 1 h at RT. The extracts were transferred to clean glass tubes. To these, 1.5 ml of chloroform and 1.5 ml of 1 M KCl were added, and the samples were vigorously shaken. After centrifugation at 3,000 rpm for 3 min the upper phase was discarded and the organic phase was washed once with 2 ml of water. After centrifugation at 3,000 rpm for 3 min the organic (bottom) phase was transferred to a clean tube, dried down under a gentle stream of nitrogen and used for FA transmethylation. 1 ml of methanol HCl (1.25 M) was added to the dried lipid extract. Samples were vortexed and incubated at 80°C for 1 h. Tubes were allowed to cool to room temperature and reactions were terminated with 1 ml of 0.9% NaCl aqueous solution. After addition of 1 ml of hexane the samples were shaken, then centrifuged at 1000 × g for 1 min to facilitate the phase separation. The upper phase (organic) containing FAMEs was transferred to GC vials. FAME samples were analyzed using an Agilent HP 6890 Series GC system equipped with 7683 Series Injector and autosampler. FAME samples were injected on BPX70 (SGE Analytical Science, Ringwood Victoria, Australia) capillary column (10 m × 0.1 mm × 0.2 um) with 50:1 split ratio and separated with constant pressure 25 psi and a temperature program: hold at 145°C for 5 min, 145–175°C at 2°C/min, hold 175°C for 1 min, 175–250°C at 30°C/min. Integration events were detected between 9 and 20 min and identified by comparing to GLC-10 FAME standard mix (Sigma).

### Accession numbers

All accession numbers for genes described in this study are provided in Additional file 4.

### Availability of supporting data

The sequence data supporting the results of this article are available in the GenBank [GenBank; *Gossypium* species: KF460111-KF460114, KF460117-KF460154, KF572120-KF572121; http://www.ncbi.nlm.nih.gov/genbank/] and Phytozome [Phytozome; *Arabidopsis thaliana*: AT2G29980.1, AT3G11170.1, AT5G05580.1; *Theobroma cacao*: Thecc1EG041603t1, Thecc1EG021677t1, Thecc1EG042487t1; http://www.phytozome.net/] repositories. The phylogenetic datasets are available at the LabArchives repository [http://dx.doi.org/10.6070/H4WS8R7Q].

## Additional files

Additional file 1:
**Gene cloning primers.**
Additional file 2:
**DNA sequencing primers.**
Additional file 3:
**RT-PCR primers used for measuring homoeologous gene expression in** ***G. hirsutum.***
Additional file 4:
**GenBank accession numbers.**
Additional file 5:
**Alignment of** ***FAD3-1*** **gene sequences from** ***G. herbaceum*** **(A diploid),** ***G. raimondii*** **(D diploid), and** ***G. hirsutum*** **(AD tetraploid).** The sequences of each gene were aligned using the ClustalW algorithm (http://www.ebi.ac.uk/Tools/msa/clustalw2/; [75]). The start and stop codons are highlighted in bold, and exons are underlined. Gene cloning primers are highlighted in yellow, and in some cases, restriction sites, highlighted in magenta, were included in the sequence to help facilitate subcloning. The name of each forward primer is provided between the gene name and start of the nucleotide sequence, and reverse primers are listed immediately after the end of the nucleotide sequence. The primers used for RT-PCR analysis of gene expression are highlighted green for the A homoeolog of *G. hirsutum*, while the D homoeolog primers are highlighted in blue. The names of all RT-PCR primers are listed above the highlighted sequence, and the arrows indicate whether they are forward or reverse primers. The nucleotide sequences highlighted for all reverse primers correspond to their forward sequence positions. The actual nucleotide sequence of all primers, listed 5’ to 3’, is provided in Additional files 1 and 2. Additional file 6:
**Alignment of** ***FAD3-2*** **gene sequences from** ***G. herbaceum*** **(A diploid),** ***G. raimondii*** **(D diploid), and** ***G. hirsutum*** **(AD tetraploid).** The sequences of each gene were aligned using the ClustalW algorithm (http://www.ebi.ac.uk/Tools/msa/clustalw2/; [75]). The start and stop codons are highlighted in bold, and exons are underlined. The in-frame stop codons in *FAD3-2.2* genes are highlighted in red. Gene cloning primers are highlighted in yellow, and in some cases, restriction sites, highlighted in magenta, were included in the sequence to help facilitate subcloning. The name of each forward primer is provided between the gene name and start of the nucleotide sequence, and reverse primers are listed immediately after the end of the nucleotide sequence. The primers used for RT-PCR analysis of gene expression are highlighted green for the A homoeolog of *G. hirsutum*, while the D homoeolog primers are highlighted in blue. The names of all RT-PCR primers are listed above the highlighted sequence, and the arrows indicate whether they are forward or reverse primers. The nucleotide sequences highlighted for all reverse primers correspond to their forward sequence positions. The actual nucleotide sequence of all primers, listed 5’ to 3’, is provided in Additional files 1 and 2. Additional file 7:
**Alignment of** ***FAD7/8-1*** **gene sequences from** ***G. herbaceum*** **(A diploid),** ***G. raimondii*** **(D diploid), and** ***G. hirsutum*** **(AD tetraploid).** The sequences of each gene were aligned using the ClustalW algorithm (http://www.ebi.ac.uk/Tools/msa/clustalw2/; [75]). The start and stop codons are highlighted in bold, and exons are underlined. Gene cloning primers are highlighted in yellow, and in some cases, restriction sites, highlighted in magenta, were included in the sequence to help facilitate subcloning. The name of each forward primer is provided between the gene name and start of the nucleotide sequence, and reverse primers are listed immediately after the end of the nucleotide sequence. The primers used for RT-PCR analysis of gene expression are highlighted green for the A homoeolog of *G. hirsutum*, while the D homoeolog primers are highlighted in blue. The names of all RT-PCR primers are listed above the highlighted sequence, and the arrows indicate whether they are forward or reverse primers. The nucleotide sequences highlighted for all reverse primers correspond to their forward sequence positions. The actual nucleotide sequence of all primers, listed 5’ to 3’, is provided in Additional files 1 and 2. Additional file 8:
**Alignment of** ***FAD7/8-2*** **gene sequences from** ***G. herbaceum*** **(A diploid),** ***G. raimondii*** **(D diploid), and** ***G. hirsutum*** **(AD tetraploid).** The sequences of each gene were aligned using the ClustalW algorithm (http://www.ebi.ac.uk/Tools/msa/clustalw2/; [75]). The start and stop codons are highlighted in bold, and exons are underlined. Gene cloning primers are highlighted in yellow, and in some cases, restriction sites, highlighted in magenta, were included in the sequence to help facilitate subcloning. The name of each forward primer is provided between the gene name and start of the nucleotide sequence, and reverse primers are listed immediately after the end of the nucleotide sequence. The primers used for RT-PCR analysis of gene expression are highlighted green for the A homoeolog of *G. hirsutum*, while the D homoeolog primers are highlighted in blue. The names of all RT-PCR primers are listed above the highlighted sequence, and the arrows indicate whether they are forward or reverse primers. The nucleotide sequences highlighted for all reverse primers correspond to their forward sequence positions. The actual nucleotide sequence of all primers, listed 5’ to 3’, is provided in Additional files 1 and 2. Additional file 9:
**Alignment of** ***FAD7/8-3*** **gene sequences from** ***G. herbaceum*** **(A diploid),** ***G. raimondii*** **(D diploid), and** ***G. hirsutum*** **(AD tetraploid).** The sequences of each gene were aligned using the ClustalW algorithm (http://www.ebi.ac.uk/Tools/msa/clustalw2/; [75]). The start and stop codons are highlighted in bold, and exons are underlined. Gene cloning primers are highlighted in yellow, and in some cases, restriction sites, highlighted in magenta, were included in the sequence to help facilitate subcloning. The name of each forward primer is provided between the gene name and start of the nucleotide sequence, and reverse primers are listed immediately after the end of the nucleotide sequence. The primers used for RT-PCR analysis of gene expression are highlighted green for the A homoeolog of *G. hirsutum*, while the D homoeolog primers are highlighted in blue. The names of all RT-PCR primers are listed above the highlighted sequence, and the arrows indicate whether they are forward or reverse primers. The nucleotide sequences highlighted for all reverse primers correspond to their forward sequence positions. The actual nucleotide sequence of all primers, listed 5’ to 3’, is provided in Additional files 1 and 2. Additional file 10:
**RNA-seq analysis of omega-3 FAD gene expression in cotton fiber, seeds, petals and leaves.** (A) Fibers were harvested from the indicated cotton varieties at 10 and 20 DPA, which represents primary and secondary cell wall synthesis, respectively, then RNA-seq analysis was performed as described [56]. Cotton varieties are indicated along the bottom. (B) Developing seeds were harvested from the indicated cotton varieties at 10, 20, 30, or 40 DPA then RNA-seq analysis was performed as described. Similar RNA-seq analyses were performed on cotton petals (C) and leaves (D), from the indicated plant lines [59,60]. Transcripts were quantified as “reads per kilobase per million mapped reads” (RPKM). For simplicity, data for A and D homoeologous sequences were combined. Values represent average and standard deviation of three biological replicates. For data presented in panels (C) and (D), student’s t-test was used for comparison of FAD7/8-1 to FAD7/8-2, and * denotes *p* <0.05. Additional file 11:
**Primer optimization for FAD3-type genes in** ***G. hirsutum.*** The gene targets and primer pairs are listed on the left. Primer sequences are described in Additional file 3. PCR reactions were programmed with plasmid DNA containing either the target gene or the closest related homoeolog (listed beneath each gel picture). A gradient of annealing temperatures, with values listed above each gel, was used during the PCR reactions, then an equal volume of each reaction was analyzed by DNA gel electrophoresis and ethidium bromide staining. The optimal anneal temperature for each primer pair, where DNA fragments can be detected for the target gene, but not the homoeolog, is reported to the right. Also listed on the right are the expected sizes of PCR fragments amplified from either genomic DNA or mRNA. Note that the plasmid DNA templates contained genomic copies of each gene, and bands of expected sizes were obtained for all PCR reactions (DNA ladder not shown). Additional file 12:
**Primer optimization for FAD7/8-type genes in** ***G. hirsutum.*** (A) The gene targets and primer pairs are listed on the left. Primer sequences are described in Additional file 3. PCR reactions were programmed with plasmid DNA containing either the target gene or the closest related homoeolog, as indicated above each column of gel pictures. A gradient of annealing temperatures, with values listed immediately above the gel pictures, was used during the PCR reactions, then an equal volume of each reaction was analyzed by DNA gel electrophoresis and ethidium bromide staining. The optimal anneal temperature for each primer pair, where DNA fragments could be detected for the target gene, but not the homoeolog, is reported to the right. Also listed are the expected sizes of PCR fragments amplified from either genomic DNA or mRNA. Note that the plasmid DNA templates contained genomic copies of each gene, and bands of expected sizes were obtained for all PCR reactions (DNA ladder not shown). (B) RT-PCR analysis of RNA extracted from 12-day-old *G. hirsutum* cotyledons, showing that *FAD7/8-1* and *FAD7/8-2* genes, from both subgenomes, are predominantly expressed. Bands of expected sizes, as amplified from cDNA and not genomic DNA, were detected, and no bands were observed in the actin control when PCR reactions were programmed with RNA that was not subjected to reverse transcription (no RT).
