# Supplementary material for: Genome-wide analysis of the omega-3 fatty acid desaturase gene family in Gossypium
Source: BMC Plant Biol. 2014 Nov 18;14:312. doi: 10.1186/s12870-014-0312-5 (PMC4245742; doi:10.1186/s12870-014-0312-5)
Supplement: Additional file 1: — Gene cloning primers. [file 12870_2014_312_MOESM1_ESM.pdf]

Additional file 1 – Gene cloning primers

Primer name	Sequence (5’ to 3’)	Notes
Degenerate primers		
C1F	TTRGGNCAYGAYTGYGGNCA	This study
C2R	YTGNGGRAACAAGTGGTG	This study
C3F	GTGGACATGGGAGTTTYTCNGA	Mikolajczyk et al., 2010
C4R	TGGCATCGACCAARTGRTARTG	Mikolajczyk et al., 2010
C5F	AARATTGCTGARATTAGRGC	Torres-Franklin et al., 2009
C6R	CCAGTRTCACTAACRAARTGRTC	Torres-Franklin et al., 2009
C7F	GCTTGTTGGACTGCAATGGC	Torres-Franklin et al., 2009
C8R	GGGATYTGHGGGAARAGATGATG	Torres-Franklin et al., 2009
FAD3-1 primers		
C9F	CAAAGAGAGACGGGGAACTG	Blunt-end cloning
C10R	AGCGAAAAGAATGAAGGCCC	Blunt-end cloning
C11F	AATGAGCTCCGGGGAACTGAAAGAATG	*Sac*I site underlined
C12R	CCCGCATGCTCTAAATAAAATGGCTCA	*SphI* site underlined
FAD3-2 primers		
C26F	AAGGTTCGAAAGGAGTGGAGATA	Blunt-end cloning
C28R	ATGAACAAATTATGGCCCTTAA	Blunt-end cloning
C29F	AAAGAGCTCATGGAGTTGAAAAGGGAG	*Sac*I site underlined
C31R	GGGGCATGCATCTTAGTTATTGTATCA	*SphI* site underlined
FAD7/8-1 primers		
C13F	CGGTGGTTGAGTTTTTCTTTTTATCAG	Blunt-end cloning
C14R	CCATTGTTGTGGGGCTGAGCTA	Blunt-end cloning
C15F	CCCGAGCTCTTTTTATCAGAGTCTTCAATG	*Sac*I site underlined
C16R	TTGGCATGCGGGGCTGAGCTATTTTA	*Sph*I site underlined
C27F	GGTGGTTGAGTTTTTCTTTTTACCTG	Blunt-end cloning
C30R	CCATTAGTGTGGGGCTGAGCTA	Blunt-end cloning
FAD7/8-2 primers		
C17F	GGCTGAACCTTTGGGAACC	Blunt-end cloning
C18R	CCGAATAGAGGTTTTAGCCAGA	Blunt-end cloning
C19F	TTAGAGCTCTAGCATCAGCCTCCAATG	*Sac*I site underlined
C20R	TTAGCATGCAGAAACTTCAGGGCTTCA	*Sph*I site underlined
FAD7/8-3 primers		
C21F	TGAGGTTCAATTTCCAAGTCGC	Blunt-end cloning
C22R	ATGGCCATACAAAGTTCAGTGCT	Blunt-end cloning
C23R	TTGAAGTTTTATGAAGCATCA	Blunt-end cloning
C24F	GGTGAGCTCTACCTGGGATTTTAATGG	*Sac*I site underlined
C25R	TTAGCATGCTCATGCTGACTTGAAAAT	*Sph*I site underlined

Mikolajczyk K, Dabert M, Karlowski WM, Spasibionek S, Nowakowska J, Cegielska-Taras T, Bartkowiak-Broda I: **Allele-specific SNP markers for the new low linolenic mutant genotype of winter oilseed rape.** *Plant Breed* 2010, **129:**502-507.

Torres-Franklin M-L, Repellina A, Huynh V-B, d’Arcy-Lameta A, Zuily-Fodila Y, Pham-Thi AT: **Omega-3 fatty acid desaturase (FAD3, FAD7, FAD8) gene expression and linolenic acid content in cowpea leaves submitted to drought and after rehydration.** *Environ Exp Bot* 2009, **65:**162-169.
