# Supplementary figures and images for: Genome-wide analysis of the omega-3 fatty acid desaturase gene family in Gossypium
Source: BMC Plant Biol. 2014 Nov 18;14:312. doi: 10.1186/s12870-014-0312-5 (PMC4245742; doi:10.1186/s12870-014-0312-5)

A

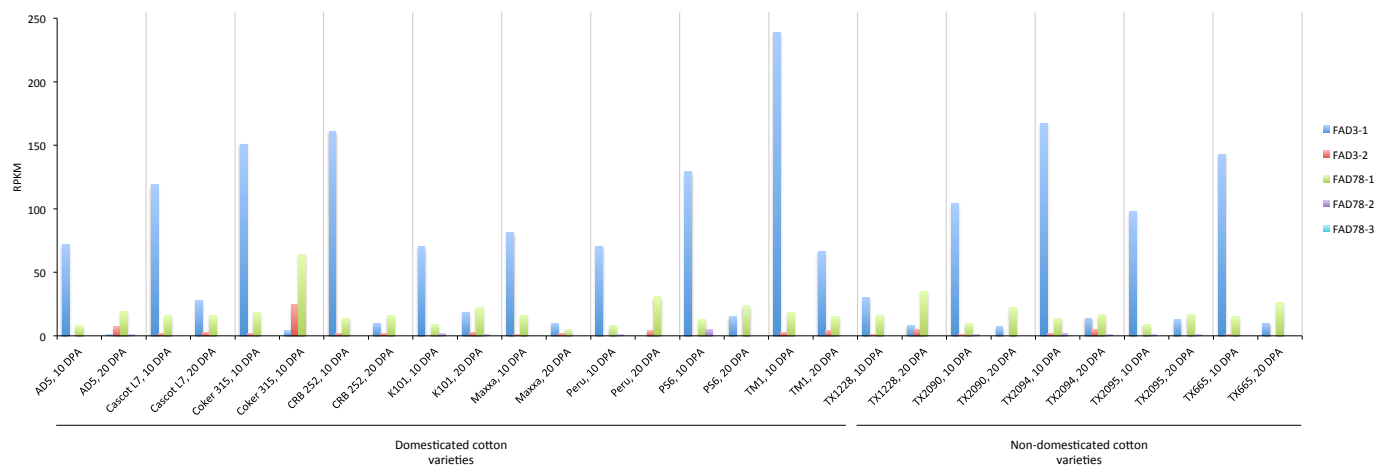

B

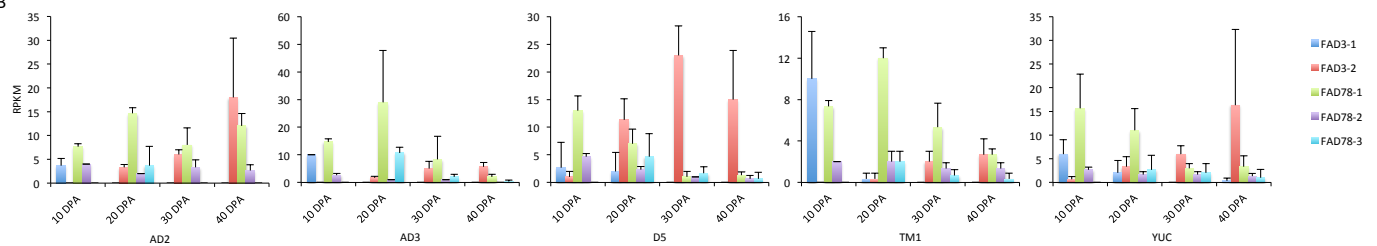

C

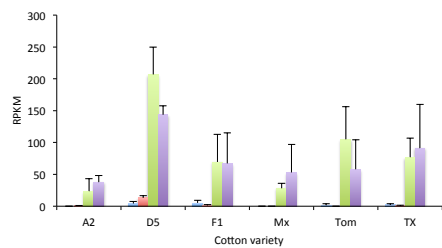

D

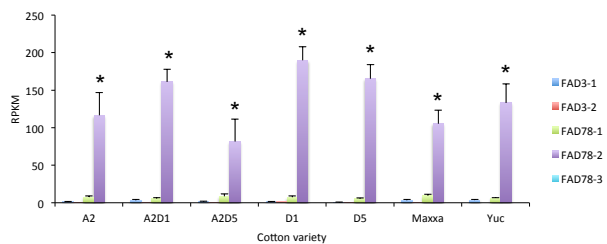

Supplement: Additional file 2: — DNA sequencing primers. [file 12870_2014_312_MOESM2_ESM.pdf]
