# Supplementary material for: Genome-wide analysis of the omega-3 fatty acid desaturase gene family in Gossypium
Source: BMC Plant Biol. 2014 Nov 18;14:312. doi: 10.1186/s12870-014-0312-5 (PMC4245742; doi:10.1186/s12870-014-0312-5)
Supplement: Additional file 3: — RT-PCR primers used for measuring homoeologous gene expression in G. hirsutum. [file 12870_2014_312_MOESM3_ESM.pdf]

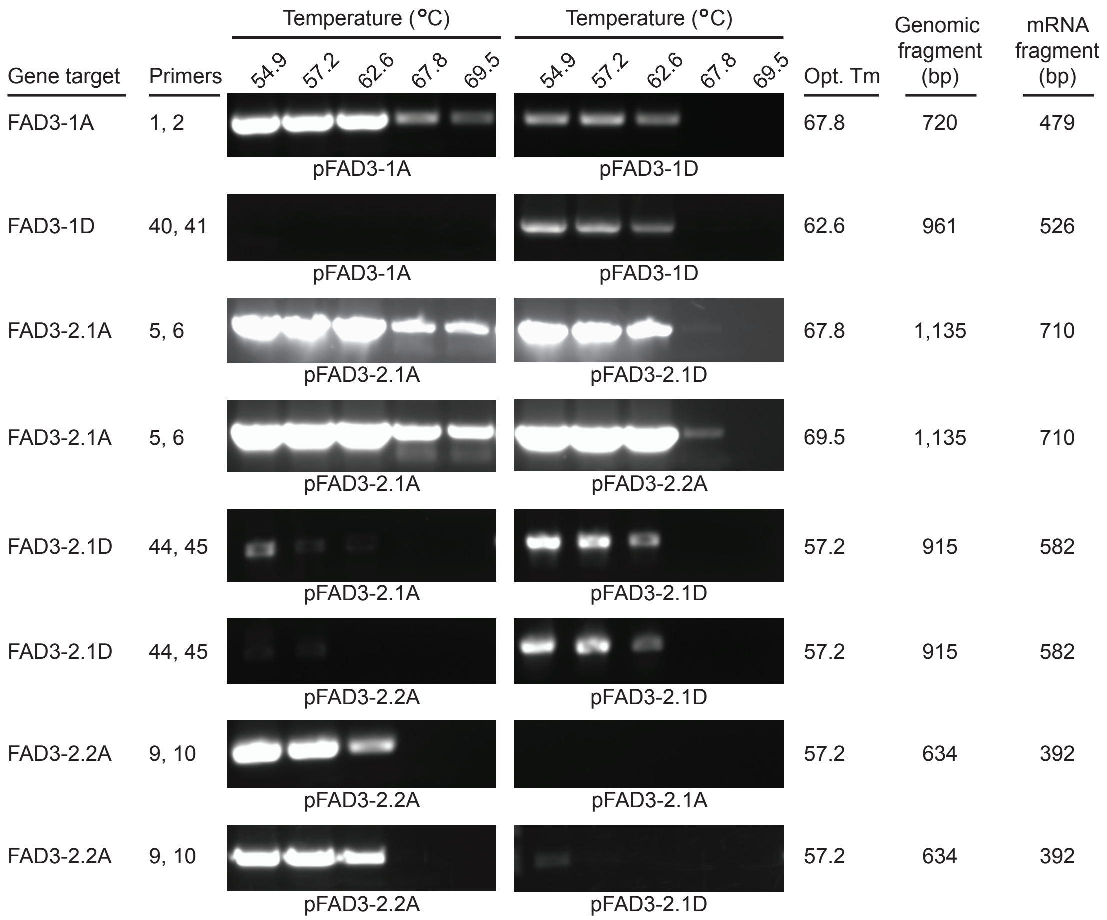

Temperature (°C)
Temperature (°C)
Gene target
Primers
54.9
57.2
62.6
67.8
69.5
54.9
57.2
62.6
67.8
69.5
Opt. Tm
Genomic fragment (bp)
mRNA fragment (bp)
FAD3-1A
1, 2
pFAD3-1A
pFAD3-1D
67.8
720
479
FAD3-1D
40, 41
pFAD3-1A
pFAD3-1D
62.6
961
526
FAD3-2.1A
5, 6
pFAD3-2.1A
pFAD3-2.1D
67.8
1,135
710
FAD3-2.1A
5, 6
pFAD3-2.1A
pFAD3-2.2A
69.5
1,135
710
FAD3-2.1D
44, 45
pFAD3-2.1A
pFAD3-2.1D
57.2
915
582
FAD3-2.1D
44, 45
pFAD3-2.2A
pFAD3-2.1D
57.2
915
582
FAD3-2.2A
9, 10
pFAD3-2.2A
pFAD3-2.1A
57.2
634
392
FAD3-2.2A
9, 10
pFAD3-2.2A
pFAD3-2.1D
57.2
634
392
