# Supplementary material for: Genome-wide analysis of the omega-3 fatty acid desaturase gene family in Gossypium
Source: BMC Plant Biol. 2014 Nov 18;14:312. doi: 10.1186/s12870-014-0312-5 (PMC4245742; doi:10.1186/s12870-014-0312-5)
Supplement: Additional file 4: — GenBank accession numbers. [file 12870_2014_312_MOESM4_ESM.pdf]

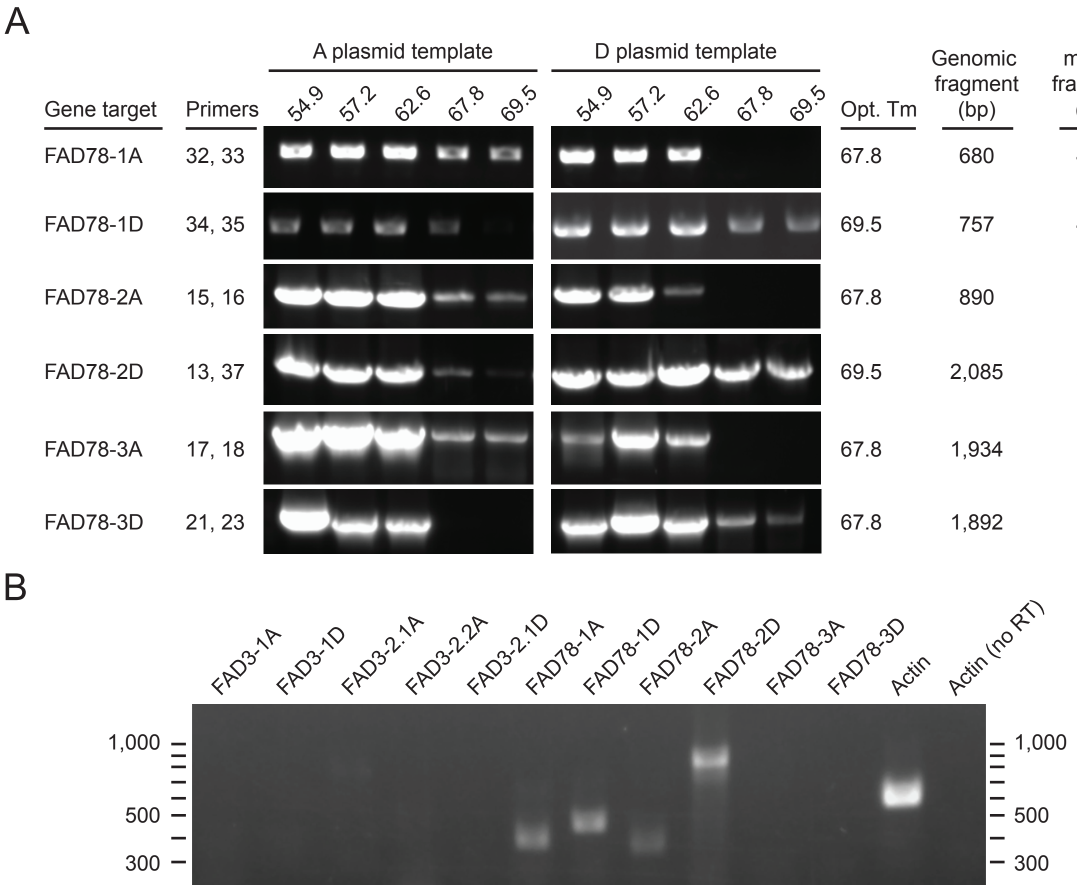
A
A plasmid template
D plasmid template
Gene target
Primers
54.9
57.2
62.6
67.8
69.5
54.9
57.2
62.6
67.8
69.5
Opt. Tm
Genomic fragment (bp)
mRNA fragment (bp)
FAD78-1A 32, 33 67.8 680 402
FAD78-1D 34, 35 69.5 757 484
FAD78-2A 15, 16 67.8 890 367
FAD78-2D 13, 37 69.5 2,085 844
FAD78-3A 17, 18 67.8 1,934 552
FAD78-3D 21, 23 67.8 1,892 737
B
FAD3-1A
FAD3-1D
FAD3-2.1A
FAD3-2.2A
FAD3-2.1D
FAD78-1A
FAD78-1D
FAD78-2A
FAD78-2D
FAD78-3A
FAD78-3D
Actin
Actin (no RT)
1,000
500
300
1,000
500
300
