# Supplementary material for: Genome-wide analysis of the omega-3 fatty acid desaturase gene family in Gossypium
Source: BMC Plant Biol. 2014 Nov 18;14:312. doi: 10.1186/s12870-014-0312-5 (PMC4245742; doi:10.1186/s12870-014-0312-5)
Supplement: Additional file 5: — Alignment of FAD3-1 gene sequences from G. herbaceum (A diploid), G. raimondii (D diploid), and G. hirsutum (AD tetraploid). The sequences of each gene were aligned using the ClustalW algorithm (http://www.ebi.ac.uk/Tools/msa/clustalw2/; [75]). The start and stop codons are highlighted in bold, and exons are underlined. Gene cloning primers are highlighted in yellow, and in some cases, restriction sites, highlighted in magenta, were included in the sequence to help facilitate subcloning. The name of each forward primer is provided between the gene name and start of the nucleotide sequence, and reverse primers are listed immediately after the end of the nucleotide sequence. The primers used for RT-PCR analysis of gene expression are highlighted green for the A homoeolog of G. hirsutum, while the D homoeolog primers are highlighted in blue. The names of all RT-PCR primers are listed above the highlighted sequence, and the arrows indicate whether they are forward or reverse primers. The nucleotide sequences highlighted for all reverse primers correspond to their forward sequence positions. The actual nucleotide sequence of all primers, listed 5’ to 3’, is provided in Additional files 1 and 2. [file 12870_2014_312_MOESM5_ESM.pdf]

Additional file 2 – DNA sequencing primers

Primer name	Sequence (5' to 3')
FAD3-1seq1F	TTCCGTAGCTGGTTCTTTTGG
FAD3-1seq2F	TCACTTCAACCCATACAGCAACTT
FAD3-1seq3F	GGGCTTACAACAATAGACCGTGA
FAD3-1seq1R	CTGAGGAAAGAGATGATGAATGACA
FAD3-1seq2R	GGATGGACCGATTACAAAGGA
FAD3-1seq3R	GTTTTGATGGTGAGTTCTATGGCTT
FAD3-2seq1F	GTGGTGGGACATATCTTACATTCTTC
FAD3-2seq2F	CGGTACAACATTTTCGACCTT
FAD3-2seq3F	TACATGACAGGAATGGAGTTATCT
FAD3-2seq1R	ACGAAAATCTACGAAACCAGAAT
FAD3-2seq2R	TCATCACAAGTTTCCTCTCTTGGG
FAD3-2seq3R	GTTCTGTGGCTTATTCTCCTGT
FAD7/8-1seq1F	AACAAGAACAAGAGGCAGGG
FAD7/8-1seq2F	AGAATTAGCCACAGGACTCACCA
FAD7/8-1seq3F	CGAGAGCCGGAACAATCAG
FAD7/8-1seq1R	TCCATCCATAGTCACGGTCAAG
FAD7/8-1seq2R	CAAATCACTGCTGGGGTCGA
FAD7/8-1seq3R	CAGTCATGACCAAGAACAAAAAGAG
FAD7/8-2seq1F	TGGTGCAGCACCTCCTTTTA
FAD7/8-2seq2F	GCAGGTCATCTTCTTCATTCTTC
FAD7/8-2seq3F	GCTTGTTGGACGGCAATG
FAD7/8-2seq4F	ATCATCTCTTCCCTCAAATCCC
FAD7/8-2seq1R	CGTCGTCAGCCCTCCTCT
FAD7/8-2seq2R	CGGATGGAAATGTGAACCC
FAD7/8-2seq3R	GCAACACTATTCAAAGCTGGATT
FAD7/8-2seq4R	TGTCACTTTCAATGGAGCACT
FAD7/8-3seq1F	ATCTATTGCATTCTTTCATTCTTG
FAD7/8-3seq2F	TACATCCAGCTTATGTTGGACA
FAD7/8-3seq3F	CCACAATTAGCCAACTAGTGAC
FAD7/8-3seq4F	TAAACTAGTCCCTTACGTTAAATCA
FAD7/8-3seq1R	AAATAAGGCAAAATGCAATCTAA
FAD7/8-3seq2R	CATAATAGCAACTTTAATACTCAATG
FAD7/8-3seq3R	TGTCCAACATAAGCTGGATGTA
FAD7/8-3seq4R	CAAGAATGAAAGAATGCAATAGAT
