# Supplementary material for: Genome-wide analysis of the omega-3 fatty acid desaturase gene family in Gossypium
Source: BMC Plant Biol. 2014 Nov 18;14:312. doi: 10.1186/s12870-014-0312-5 (PMC4245742; doi:10.1186/s12870-014-0312-5)
Supplement: Additional file 6: — Alignment of FAD3-2 gene sequences from G. herbaceum (A diploid), G. raimondii (D diploid), and G. hirsutum (AD tetraploid). The sequences of each gene were aligned using the ClustalW algorithm (http://www.ebi.ac.uk/Tools/msa/clustalw2/; [75]). The start and stop codons are highlighted in bold, and exons are underlined. The in-frame stop codons in FAD3-2.2 genes are highlighted in red. Gene cloning primers are highlighted in yellow, and in some cases, restriction sites, highlighted in magenta, were included in the sequence to help facilitate subcloning. The name of each forward primer is provided between the gene name and start of the nucleotide sequence, and reverse primers are listed immediately after the end of the nucleotide sequence. The primers used for RT-PCR analysis of gene expression are highlighted green for the A homoeolog of G. hirsutum, while the D homoeolog primers are highlighted in blue. The names of all RT-PCR primers are listed above the highlighted sequence, and the arrows indicate whether they are forward or reverse primers. The nucleotide sequences highlighted for all reverse primers correspond to their forward sequence positions. The actual nucleotide sequence of all primers, listed 5’ to 3’, is provided in Additional files 1 and 2. [file 12870_2014_312_MOESM6_ESM.pdf]

Additional file 3 – RT-PCR primers used for measuring homoeologous gene expression in *G. hirsutum*

Primer name	Gene target	Sequence (5’ to 3’)
FAD3-1 primers		
S1F	GhiFAD3-1A	GTGGTTTTCCTTGTGTATTTATCCTC
S2R	GhiFAD3-1A	CATGCGGATTTGGGAGGA
S40F	GhiFAD3-1D	CCATGTTTTGGGCTC
S41R	GhiFAD3-1D	TCGTATCCGTGGTGG
FAD3-2 primers		
S5F	GhiFAD3-2.1A	GAACCACGGAAATGTCGAAAAT
S6R	GhiFAD3-2.1A	GCAACTTAAACAGGTTTAGATCAGTCC
S9F	GhiFAD3-2.2A	CTCCTCAATTCCGGATTGCC
S10R	GhiFAD3-2.2A	GTAAATATTCTCAGGCAACGGC
S44F	GhiFAD3-2.1D	TGAAAAGGGAGTATGCC
S45R	GhiFAD3-2.1D	TAAACAAGTTGCTGTAAGGA
FAD7/8-1 primers		
S32F	GhiFAD7/8-1A	ATTTGTTTGTCCCGACCGAAC
S33R	GhiFAD7/8-1A	CCGGCTCTCGATAGTATTTTCG
S34F	GhiFAD7/8-1D	GTCCCGACTGAAAGAAAAGATGTG
S35R	GhiFAD7/8-1D	CAACATCCCCAGTGTCACTAACATAG
FAD7/8-2 primers		
S15F	GhiFAD7/8-2A	GGCTTGCTTGCATATTTGTCG
S16R	GhiFAD7/8-2A	GTAAATAAAATGGTAAAGGCCCG
S13F	GhiFAD7/8-2D	AGATAAAGGTAAAGAAGAGAGCACCAC
S37R	GhiFAD7/8-2D	ATCCCGATCAAGCGTCGTC
FAD7/8-3 primers		
S17F	GhiFAD7/8-3A	TTGGATAACAATGAACTAACATGGCT
S18R	GhiFAD7/8-3A	GATGAGGTGAAAAGGTAGAGGTCTC
S21F	GhiFAD7/8-3D	TTGGAGAGCAAGAAGAAGAGACAAT
S23R	GhiFAD7/8-3D	TGATGCATGTATGTTACAAAATCCAA
