# Supplementary material for: Genome-wide analysis of the omega-3 fatty acid desaturase gene family in Gossypium
Source: BMC Plant Biol. 2014 Nov 18;14:312. doi: 10.1186/s12870-014-0312-5 (PMC4245742; doi:10.1186/s12870-014-0312-5)
Supplement: Additional file 7: — Alignment of FAD7/8-1 gene sequences from G. herbaceum (A diploid), G. raimondii (D diploid), and G. hirsutum (AD tetraploid). The sequences of each gene were aligned using the ClustalW algorithm (http://www.ebi.ac.uk/Tools/msa/clustalw2/; [75]). The start and stop codons are highlighted in bold, and exons are underlined. Gene cloning primers are highlighted in yellow, and in some cases, restriction sites, highlighted in magenta, were included in the sequence to help facilitate subcloning. The name of each forward primer is provided between the gene name and start of the nucleotide sequence, and reverse primers are listed immediately after the end of the nucleotide sequence. The primers used for RT-PCR analysis of gene expression are highlighted green for the A homoeolog of G. hirsutum, while the D homoeolog primers are highlighted in blue. The names of all RT-PCR primers are listed above the highlighted sequence, and the arrows indicate whether they are forward or reverse primers. The nucleotide sequences highlighted for all reverse primers correspond to their forward sequence positions. The actual nucleotide sequence of all primers, listed 5’ to 3’, is provided in Additional files 1 and 2. [file 12870_2014_312_MOESM7_ESM.pdf]

Additional file 4 – Gene accession numbers

Name	Organism	Accession number (G – GenBank; P – Phytozome)
Ath_FAD3	*Arabidopsis thaliana*	AT2G29980.1 (P)
Ath_FAD7	*Arabidopsis thaliana*	AT3G11170.1 (P)
Ath_FAD8	*Arabidopsis thaliana*	AT5G05580.1 (P)
Ghe_FAD3-1A (gene)	*Gossypium herbaceum*	KF460121 (G)
Ghe_FAD3-1A (mRNA)	*Gossypium herbaceum*	KF460122 (G)
Ghe_FAD3-2.1A (gene)	*Gossypium herbaceum*	KF460111 (G)
Ghe_FAD3-2.1A (mRNA)	*Gossypium herbaceum*	KF460112 (G)
Ghe_FAD3-2.2A (gene)	*Gossypium herbaceum*	KF460113 (G)
Ghe_FAD3-2.2A (mRNA)	*Gossypium herbaceum*	KF460114 (G)
Ghe_FAD78-1A (gene)	*Gossypium herbaceum*	KF572120 (G)
Ghe_FAD78-1A (mRNA)	*Gossypium herbaceum*	KF572121 (G)
Ghe_FAD78-2A (gene)	*Gossypium herbaceum*	KF460117 (G)
Ghe_FAD78-2A (mRNA)	*Gossypium herbaceum*	KF460118 (G)
Ghe_FAD78-3A (gene)	*Gossypium herbaceum*	KF460119 (G)
Ghe_FAD78-3A (mRNA)	*Gossypium herbaceum*	KF460120 (G)
Ghi_FAD3-1A (gene)	*Gossypium hirsutum*	KF460123 (G)
Ghi_FAD3-1A (mRNA)	*Gossypium hirsutum*	KF460124 (G)
Ghi_FAD3-1D (gene)	*Gossypium hirsutum*	KF460125 (G)
Ghi_FAD3-1D (mRNA)	*Gossypium hirsutum*	KF460126 (G)
Ghi_FAD3-2.1A (gene)	*Gossypium hirsutum*	KF460127 (G)
Ghi_FAD3-2.1A (mRNA)	*Gossypium hirsutum*	KF460128 (G)
Ghi_FAD3-2.1D (gene)	*Gossypium hirsutum*	KF460129 (G)
Ghi_FAD3-2.1D (mRNA)	*Gossypium hirsutum*	KF460130 (G)
Ghi_FAD3-2.2A (gene)	*Gossypium hirsutum*	KF460131 (G)
Ghi_FAD3-2.2A (mRNA)	*Gossypium hirsutum*	KF460132 (G)
Ghi_FAD78-1A (gene)	*Gossypium hirsutum*	KF460133 (G)
Ghi_FAD78-1A (mRNA)	*Gossypium hirsutum*	KF460134 (G)
Ghi_FAD78-1D (gene)	*Gossypium hirsutum*	KF460135 (G)
Ghi_FAD78-1D (mRNA)	*Gossypium hirsutum*	KF460136 (G)
Ghi_FAD78-2A (gene)	*Gossypium hirsutum*	KF460137 (G)
Ghi_FAD78-2A (mRNA)	*Gossypium hirsutum*	KF460138 (G)
Ghi_FAD78-2D (gene)	*Gossypium hirsutum*	KF460139 (G)
Ghi_FAD78-2D (mRNA)	*Gossypium hirsutum*	KF460140 (G)
Ghi_FAD78-3A (gene)	*Gossypium hirsutum*	KF460141 (G)
Ghi_FAD78-3A (mRNA)	*Gossypium hirsutum*	KF460142 (G)
Ghi_FAD78-3D (gene)	*Gossypium hirsutum*	KF460143 (G)
Ghi_FAD78-3D (mRNA)	*Gossypium hirsutum*	KF460144 (G)
Gra_FAD3-1D (gene)	*Gossypium raimondii*	KF460145 (G)
Gra_FAD3-1D (mRNA)	*Gossypium raimondii*	KF460146 (G)
Gra_FAD3-2.1D (gene)	*Gossypium raimondii*	KF460147 (G)
Gra_FAD3-2.1D (mRNA)	*Gossypium raimondii*	KF460148 (G)
Gra_FAD78-1D (gene)	*Gossypium raimondii*	KF460149 (G)
Gra_FAD78-1D (mRNA)	*Gossypium raimondii*	KF460150 (G)
Gra_FAD78-2D (gene)	*Gossypium raimondii*	KF460151 (G)
Gra_FAD78-2D (mRNA)	*Gossypium raimondii*	KF460152 (G)
Gra_FAD78-3D (gene)	*Gossypium raimondii*	KF460153 (G)
Gra_FAD78-3D (mRNA)	*Gossypium raimondii*	KF460154 (G)
Tca_FAD3	*Theobroma cacao*	Thecc1EG041603t1 (P)
Tca_FAD7-1	*Theobroma cacao*	Thecc1EG021677t1 (P)
Tca_FAD7-2	*Theobroma cacao*	Thecc1EG042487t1 (P)
