# Supplementary material for: Genome-wide analysis of the omega-3 fatty acid desaturase gene family in Gossypium
Source: BMC Plant Biol. 2014 Nov 18;14:312. doi: 10.1186/s12870-014-0312-5 (PMC4245742; doi:10.1186/s12870-014-0312-5)
Supplement: Additional file 8: — Alignment of FAD7/8-2 gene sequences from G. herbaceum (A diploid), G. raimondii (D diploid), and G. hirsutum (AD tetraploid). The sequences of each gene were aligned using the ClustalW algorithm (http://www.ebi.ac.uk/Tools/msa/clustalw2/; [75]). The start and stop codons are highlighted in bold, and exons are underlined. Gene cloning primers are highlighted in yellow, and in some cases, restriction sites, highlighted in magenta, were included in the sequence to help facilitate subcloning. The name of each forward primer is provided between the gene name and start of the nucleotide sequence, and reverse primers are listed immediately after the end of the nucleotide sequence. The primers used for RT-PCR analysis of gene expression are highlighted green for the A homoeolog of G. hirsutum, while the D homoeolog primers are highlighted in blue. The names of all RT-PCR primers are listed above the highlighted sequence, and the arrows indicate whether they are forward or reverse primers. The nucleotide sequences highlighted for all reverse primers correspond to their forward sequence positions. The actual nucleotide sequence of all primers, listed 5’ to 3’, is provided in Additional files 1 and 2. [file 12870_2014_312_MOESM8_ESM.pdf]

```
GraFAD3-1D  C9F     CAAAGAGAGACGGGGAACTGGAAGAATGAGCCCTTCAATGAAGGAAACATCATTAGAAAC 60
GhiFAD3-1D C11F      AATGAGCTCCGGGGAACTGAAAGAATGAGCCCTTCAATGAAGGAAACATCATTAGAAAC 59
GheFAD3-1A  C9F     CAAAGAGAGACGGGGAACTGAAAGAATGAGCCCTTCAATGAAGGAAACATCATTAGAAAC 60
GhiFAD3-1A C11F      AATGAGCTCCGGGGAACTGAAAGAATGAGCCCTTCAATGAAGGAAACATCATTAGAAAC 59
                     ** ***   ********** ***************************************

GraFAD3-1D          AATGGAGAAAAAGGGAGAGGATGTTAATGGGATAATGAAGAAGGAGCAAGATAAGTTGTT 120
GhiFAD3-1D          AATGGAGAAAAAGGGAGAGGATGTTAATGGGATAATGAAGAAGGAGCAAGATAAGTTGTT 119
GheFAD3-1A          AATGGAGAAAAAGGGTGAGGATGTTAATGGGATAATGAAGAAGGAGCAAGATAAGTTGTT 120
GhiFAD3-1A          AATGGAGAAAAAGGGTGAGGATGTTAATGGGATAATGAAGAAGGAGCAAGATAAGTTGTT 119
                    *************** ********************************************

GraFAD3-1D          CGATCCAAGTGAAGCCCCTCCTTTCCGTATTAATGATATCCGAGCTGCTATTCCAAAGCA 180
GhiFAD3-1D          CGATCCAAGTGAAGCCCCTCCTTTCCGTATTAATGATATCCGAGCTGCTATTCCAAAGCA 179
GheFAD3-1A          CGATCCAAGTGAAGCCCCTCCTTTCCGTATTAATGATATCCGAGCTGCTATTCCAAAGCA 180
GhiFAD3-1A          CGATCCAAGTGAAGCCCCTCCTTTCCGTATTAATGATATCCGAGCTGCTATTCCAAAGCA 179
                    ************************************************************

GraFAD3-1D          CTGCTGGGTCAAGAACCCTTGGAGGTCTATGAGTTATGTTTTCAGGGACGCCATTGTCAT 240
GhiFAD3-1D          CTGCTGGGTCAAGAACCCTTGGAGGTCTATGAGTTATGTTTTCAGGGACGCCATTGTCAT 239
GheFAD3-1A          CTGTTGGGTCAAGAACCCTTGGAGGTCTATGAGTTATGTTTTCAGGGACGCCATTGTCAT 240
GhiFAD3-1A          CTGTTGGGTCAAGAACCCTTGGAGGTCTATGAGTTATGTTTTCAGGGACGCCATTGTCAT 239
                    *** ********************************************************

GraFAD3-1D          CTCTGCACTCTTAGCAGCTGCACTTTATTTCCGTAGCTGGTTCTTTTGGCCTGTCTACTG 300
GhiFAD3-1D          CTCTGCACTCTTAGCAGCTGCACTTTATTTCCGTAGCTGGTTCTTTTGGCCTTTCTACTG 299
GheFAD3-1A          CTCTGCACTCTTAGCAGCTGCACTTTATTTCCGTAGCTGGTTCTTTTGGCCTTTCTACTG 300
GhiFAD3-1A          CTCTGCACTCTTAGCAGCTGCACTTTATTTCCGTAGCTGGTTCTTTTGGCCTTTCTACTG 299
                    **************************************************** *******
                                  -----S40F----->
GraFAD3-1D          GGTTGCCCAAGGGACCATGTTTTGGGCTCTCTTTGTCCTTGGACATGATTGGTACCACGA 360
GhiFAD3-1D          GGTTGCCCAAGGGACCATGTTTTGGGCTCTCTTTGTCCTTGGACATGATTGGTACCACGA 359
GheFAD3-1A          GGTTGCCCAAGGGACCATGTTTTGGGCTCTCTTTGTCCTTGGACATGATTGGTACAACGA 360
GhiFAD3-1A          GGTTGCCCAAGGGACCATGTTTTGGGCTGTCTTTGTCCTTGGACATGATTGGTACAACGA 359
                    **************************** ************************** ****

GraFAD3-1D          CTCATTTTATCAATCTCTTTTTCACTTCCATTTTCCATTTCGGTTAATTAACTCCTGGGA 420
GhiFAD3-1D          CTCATTTTATCAATCTCTTTTTCACTTCCATTTTCCATTTCGGTTAATTAACTCCTGGGA 419
GheFAD3-1A          CTCATTTTATCAATCTCTTTTTCACTTCCATTTTCCATTTCGGTTAATTAACTCCTGGGA 420
GhiFAD3-1A          CTCATTTTATCAATCTCTTTTTCACTTCCATTTTCCATTTCGGTTAATTAACTCCTGGGA 419
                    ************************************************************

GraFAD3-1D          TTTTTGTTTTTGTTTTTGCAGTGGACATGGAAGCTTCTCTGATAATCCCATACTTAATAA 480
GhiFAD3-1D          TTTTTGTTTTTGTTTTTGCAGTGGACATGGAAGCTTCTCTGATAATCCCATACTTAATAA 479
GheFAD3-1A          TTTTTGTTTTTGTTTTTGCAGTGGACATGGAAGCTTCTCTGATAATCCCATACTTAATAA 480
GhiFAD3-1A          TTTTTGTTTTTGTTTTTGCAGTGGACATGGAAGCTTCTCTGATAATCCCATACTTAATAA 479
                    ************************************************************

GraFAD3-1D          CGTGATGGGACATATCTTACATTCTACCATCCTTGTTCCTTACCATGGATGGTAAGTTCA 540
GhiFAD3-1D          CGTGATGGGACATATCTTACATTCTACCATCCTTGTTCCTTACCATGGATGGTAAGTTCA 539
GheFAD3-1A          CGTGATGGGACATATCTTACATTCTACCATCCTTGTTCCTTACCATGGATGGTAAGTTCA 540
GhiFAD3-1A          CGTGATGGGACATATCTTACATTCTACCATCCTTGTTCCTTACCATGGATGGTAAGTTCA 539
                    ************************************************************

GraFAD3-1D          TGACTTGATTAGCTCAACATTTGTTCTTCATATACTATGGAACTTCATTTGAACAATTTC 600
GhiFAD3-1D          TGACTTGATTAGCTCAACATTTGTTCTTCATATACTATGGAACTTCATTTGAACAATTTC 599
GheFAD3-1A          TTACTTGATTAGCTCAACATTTGTTCTTCATATACTATGGAACTTCATTTGAACAATTTT 600
GhiFAD3-1A          TTAATTGATTAGCTCAACATTTGTTCTTCATATACTATGGAACTTCATTTGAACAATTTT 599
                    * * *******************************************************

GraFAD3-1D          TTATATAGGAGAATAAGCCATAGAACTCACCATCAAAACCATGGAAATGTGGAGAAGGAT 660
GhiFAD3-1D          TTATATAGGAGAATAAGCCATAGAACTCACCATCAAAACCATGGAAATGTGGAGAAGGAT 659
GheFAD3-1A          TTATATAGGAGAATAAGCCATAGAACTCACCATCAAAACCATGGAAATGTGGAGAAGGAT 660
GhiFAD3-1A          TTATATAGGAGAATAAGCCATAGAACTCACCATCAAAACCATGGAAATGTGGAGAAGGAT 659
                    ************************************************************

GraFAD3-1D          GAGTCTTGGGTTCCGGTATGTATGTCGAATTGAATTCAGTTAGTTCATGCAGTCGAAACT 720
GhiFAD3-1D          GAGTCTTGGGTTCCGGTATGTATGTCGAATTGAATTCAGTTAGTTCATGCAGTCGAAACT 719
GheFAD3-1A          GAGTCTTGGGTTCCGGTATGTATATCGAATTGAATTCAGTTAGTTCATGCAGTCGAAAAC 720
GhiFAD3-1A          GAGTCTTGGGTTCCGGTATGTATATCGAATTGAATTCAGTTAGTTCATGCAGTCGAAAAC 719
                    *********************** **********************************
```

```
GraFAD3-1D     CAAATATACATTCACTTTATATATATATACACTTTAAATTTCAGATGTCCGAAGATCTTT 780
GhiFAD3-1D     CAAATATACATTCACTTTATATATATATACACTTTAAATTTCAGATGTCCGAAGATCTTT 779
GheFAD3-1A     CAAACTTATAT-------ATATATATATATATATTTAATTTCAGATGTCCGAAGATCTTT 773
GhiFAD3-1A     CAAACTTATAT-------ATATATAT--------TTAATTTCAGATGTCCGAAGATCTTT 764
               ****  ** **       ********        * ************************

GraFAD3-1D     ATAATGGTTTGAGCAGCAGGACCAAGTTTCTGAGATTCAAAATCCCATTCCCCCTATTTG 840
GhiFAD3-1D     ATAATAGTTTGAGCAGCAGGACCAAGTTTCTGAGATTCAAAATCCCATTCCCCCTATTTG 839
GheFAD3-1A     ATAATAGTTTGAGCAGCAGGACCAAGTTTCTGAGATTCAAAATCCCATTCCCCCTATTTG 833
GhiFAD3-1A     ATAATAGTTTGAGCAGCAGGACCAAGTTTCTGAGATTCAAAATCCCATTCCCCCTATTTG 824
               ***** ******************************************************

GraFAD3-1D     CATACCCCGTCTATTTGGTGAGAGACATTGATTGAAATGCAAAAGACGATTTTAACAATT 900
GhiFAD3-1D     CATACCCTGTCTATTTGGTGAGAGACATTGATTGAAATGCAAAAGACGATTTTAACAATT 899
GheFAD3-1A     CATACCCTGTCTATTTGGTGAGAGACATTGATTGAAATGCAAAAGATGATTTTAACAATT 893
GhiFAD3-1A     CATACCCTGTCTATTTGGTGAGAGACATTGATTGAAATGCAAAAGATGATTTTAACAATT 884
               ******* ************************************** *************

GraFAD3-1D     AGTCATGATTTAACAAGATTGAAGTAAATAAACATTGGGTAAATTTTGGATATGCAGTGG 960
GhiFAD3-1D     AGTCATGATTTAACAAGATTGAAGTAAATAAACATTGGGTAAATTTTGGATATGCAGTGG 959
GheFAD3-1A     AGTCATGATTTAACAAAGTTGAAGTAAATTAACATTGGGTAAATTTTGGATATGCAGTGG 953
GhiFAD3-1A     AGTCATGATTTAACAAGATTGAAGTAAATAAACATTGGGTAAATTTTGGATATGCAGTGG 944
               ****************  *********** ******************************

GraFAD3-1D     CACAGAAGTCCAGGAAAGACAGGATCTCACTTCAACCCATACAGCAACTTGTTTGCTCCC 1020
GhiFAD3-1D     CACAGAAGTCCAGGAAAGACAGGATCTCACTTCAACCCATACAGCAACTTGTTTGCTCCC 1019
GheFAD3-1A     CACAGAAGTCCAGGAAAGACAGGATCTCACTTCAACCCATACAGCAACTTGTTTGCTCCC 1013
GhiFAD3-1A     CACAGAAGTCCAGGAAAGACAGGATCTCACTTCAACCCATACAGCAACTTGTTTGCTCCC 1004
               ************************************************************
                                                               ------------
GraFAD3-1D     CAAGAACGAAAACATATAATGACATCAACAACCTGTTGGATAGCAATGGTGGTTTTCCTT 1080
GhiFAD3-1D     CAAGAACGGAAACATATAATGACATCAACAACCTGTTGGATAGCAATGGTGGTTTTCCTT 1079
GheFAD3-1A     CAAGAACGGAAACATATAATGACATCAACAACCTGTTGGATAGCAATGGTGGTTTTCCTT 1073
GhiFAD3-1A     CAAGAACGGAAACATATAATGACATCAACAACCTGTTGGATAGCAATGGTGGTTTTCCTT 1064
               ******** ***************************************************
               ----S1F------>
GraFAD3-1D     GTGTATTTATCCTTTGTAATCGGTCCATCCATGACATTCAAGCTCTACGGTGTTCCCTAC 1140
GhiFAD3-1D     GTGTATTTATCCTTTGTAATCGGTCCATCCATGACATTCAAGCTCTACGGTGTTCCCTAC 1139
GheFAD3-1A     GTGTATTTATCCTCTGTAATCGGTCCATCCATGACATTCAAGCTCTACGGTGTTCCCTAC 1133
GhiFAD3-1A     GTGTATTTATCCTCTGTAATCGGTCCATCCATGACATTCAAGCTCTACGGTGTTCCCTAC 1124
               ************* **********************************************

GraFAD3-1D     TTGGTATGCCACTT-CTACCAAATTCACAATATATATA----TCTATCTCTGAGTAATGA 1195
GhiFAD3-1D     TTGGTATGCCACTT-CCACCAAATTCACAATATATATA----TCTATCTCTGAGTAATGA 1194
GheFAD3-1A     TTGGTATGCCACTTTCTACCAAATTCACAATATATATATATATATATCTCTGAGTAATGA 1193
GhiFAD3-1A     TTGGTATGCCACTT-CTACCAAATTCACAATATATA------TATATCTCTGAGTAATGA 1177
               ************** * *******************      * ****************

GraFAD3-1D     ATATATGATTGTTACCATTTCCATTAGATTTTCGTGGCATGGCTGGATGTGGTGACTTAC 1255
GhiFAD3-1D     ATATATGATTGTTATCATTTCCATTAGATTTTCGTGGCATGGCTGGATGTGGTGACTTAC 1254
GheFAD3-1A     ATATATGATTGTTACCATTTCCATTAGATTTTCGTGGCATGGCTGGATGTGGTGACTTAC 1253
GhiFAD3-1A     ATATATGATTGTTACCATTTCCATTAGATTTTCGTGGCATGGCTGGATGTGGTGACTTAC 1237
               ************** *********************************************
                    <-----S41R-----
GraFAD3-1D     CTTCACCACCACGGATACGAGCAGAAGCTTCCTTGGTATCGTGGCAAGGTAGTAATTACA 1315
GhiFAD3-1D     CTTCACCACCACGGATACGAGCAGAAGCTTCCTTGGTATCGTGGCAAGGTAGTAATTACA 1314
GheFAD3-1A     CTTCATCACCATGGATACGAGCAGAAGCTTCCTTGGTATCGTGGCAAGGTAGTAATTACA 1313
GhiFAD3-1A     CTTCATCACCATGGATACGAGCAGAAGCTTCCTTGGTATCGTGGCAAGGTAGTAATTACA 1297
               ***** ***** ************************************************

GraFAD3-1D     ACCC---CCCTCCATTGCATTTGACACTATCAATGATTTTGTTTGTGTGACGACTATCAG 1372
GhiFAD3-1D     ACCC---CCCTCCATTGCATTTGACACTATCAATGATTTTGTTTGTGTGACGACTATCAG 1371
GheFAD3-1A     ACCCGCCCCCTCCATTGCATTTGACACTATCAATGATTTTGTTTGTGTGACGACTATCAG 1373
GhiFAD3-1A     ACCCGCCCCCTCCATTGCATTTGACACTATCAATGATTTTGTTTGTGTGACGACTATCAG 1357
               ****   *****************************************************
```

```
GraFAD3-1D    GAATGGAGTTACCTGAGAGGAGGGCTTACAACAATAGACCGTGATTATGGTATATTCAAT 1432
GhiFAD3-1D    GAATGGAGTTACCTGAGAGGAGGGCTTACAACAATAGACCGTGATTATGGTATATTCAAT 1431
GheFAD3-1A    GAATGGAGTTACCTGAGAGGAGGGCTTACAACAATAGACCGTGATTATGGTATATTCAAT 1433
GhiFAD3-1A    GAATGGAGTTACCTGAGAGGAGGGCTTACAACAATAGACCGTGATTATGGTATATTCAAT 1417
              ************************************************************

GraFAD3-1D    GGTATCCACCATGACATTGGCACCCATGTCATTCATCATCTCTTTCCTCAGATCCCCCAT 1492
GhiFAD3-1D    GGTATCCACCATGACATTGGCACCCATGTCATTCATCATCTCTTTCCTCAGATCCCCCAT 1491
GheFAD3-1A    GGTATCCACCATGACATAGGCACCCATGTCATTCATCATCTCTTTCCTCAGATCCCCCAT 1493
GhiFAD3-1A    GGTATCCACCATGACATTGGCACCCATGTCATTCATCATCTCTTTCCTCAGATCCCCCAT 1477
              ***************** ******************************************

GraFAD3-1D    TATCACTTAGTCGAAGCTGTAAGTATTCTCAATTTTTGGGTTAATTTTAGATTACTCCTC 1552
GhiFAD3-1D    TATCACTTAGTCGAAGCTGTAAGTATTCTCAATTTTTGGGTTAATTTTAGATTACTCCTC 1551
GheFAD3-1A    TATCACTTAGTCGAAGCTGTAAGTATTCTCAATTTTTGGGTTAATTTTAGATTACTCCTC 1553
GhiFAD3-1A    TATCACTTAGTCGAAGCTGTAAGTATTCTCAATTTTTGGGTTAATTTTAGATTACTCCTC 1537
              ************************************************************

GraFAD3-1D    AAAATGTTTCCATCACCTAGCAGTAAGCTTGAAAACGGGGTGTGGTGCAGACAAAAGCAG 1612
GhiFAD3-1D    AAAATGTTTCCATCACCTAGCAGTAAGCTTGAAAACGGGGTGTGGTGCAGACAAAAGCAG 1611
GheFAD3-1A    AAAATGTTTCCATCACCTAGCAGTAAGCTTGAAAACGGGGTGTGGTGCAGACAAAAGCAG 1613
GhiFAD3-1A    AAAATGTTTCCATCACCTAGCAGTAAGCTTGAAAACGGGGTGTGGTGCAGACAAAAGCAG 1597
              ************************************************************

GraFAD3-1D    CGAAACCGGTGATAGGAAAATACTACCGGGAGCCGAAGAAATCAGGGCCGATTCCGTTTC 1672
GhiFAD3-1D    CGAAACCGGTGATAGGAAAATACTACCGGGAGCCGAAGAAATCAGGGCCGATTCCGTTTC 1671
GheFAD3-1A    CGAAACCGGTGATAGGAAAATACTACCGGGAGCCGAAGAAATCAGGGCCGATTCCGTTTC 1673
GhiFAD3-1A    CGAAACCGGTGATAGGAAAATACTACCGGGAGCCGAAGAAATCAGGGCCGATTCCGTTTC 1657
              ************************************************************

GraFAD3-1D    ACTTAATAGAGAATCTAGTGTCGAGCATGAAACAAGATCATTACGTGAGCAACAGCGGAG 1732
GhiFAD3-1D    ACTTAATAGAGAATCTAGTGTCGAGCATGAAACAAGATCATTACGTGAGCAACAGCGGAG 1731
GheFAD3-1A    ACTTAATAGAGAATCTAGTGTCGAGCATGAAACAAGATCATTACGTGAGCAACAGCGGAG 1733
GhiFAD3-1A    ACTTAATAGAGAATCTAGTGTCGAGCATGAAACAAGATCATTACGTGAGCAACAGCGGAG 1717
              ************************************************************
                                                     <-------S2R-------
GraFAD3-1D    AGATAGTATTTTATCAGACGGATCCCAACCTGTTTTCGCCTCCCAAATCCGCATGAGCCA 1792
GhiFAD3-1D    AGATAGTATTTTATCAGACGGATCCCAACCTGTTTTCGCCTCCCAAATCCGCATGAGCCA 1791
GheFAD3-1A    AGATTGTATTTTATCAGACGGATCCCAACCTGTTTTCTCCTCCCAAATCCGCATGAGCCA 1793
GhiFAD3-1A    AGATTGTATTTTATCAGACGGATCCCAACCTGTTTTCTCCTCCCAAATCCGCATGAGCCA 1777
              **** ******************************** **********************

GraFAD3-1D    TTTTATTTAGAGATCATAGAGCTGAAACAAACAAGGGCCTTCATTCTTTTCGCT C10R  1846
GhiFAD3-1D    TTTTATTTAGAGCATGCGGG                                   C12R  1811
GheFAD3-1A    TTTTATTTAGAGATCATAGAGCTGAAACAAACAAGGGCCTTCATTCTTTTCGCT C10R  1847
GhiFAD3-1A    TTTTATTTAGAGCATGCGGG                                   C12R  1797
              ************
```
