# Supplementary material for: Genome-wide analysis of the omega-3 fatty acid desaturase gene family in Gossypium
Source: BMC Plant Biol. 2014 Nov 18;14:312. doi: 10.1186/s12870-014-0312-5 (PMC4245742; doi:10.1186/s12870-014-0312-5)
Supplement: Additional file 9: — Alignment of FAD7/8-3 gene sequences from G. herbaceum (A diploid), G. raimondii (D diploid), and G. hirsutum (AD tetraploid). The sequences of each gene were aligned using the ClustalW algorithm (http://www.ebi.ac.uk/Tools/msa/clustalw2/; [75]). The start and stop codons are highlighted in bold, and exons are underlined. Gene cloning primers are highlighted in yellow, and in some cases, restriction sites, highlighted in magenta, were included in the sequence to help facilitate subcloning. The name of each forward primer is provided between the gene name and start of the nucleotide sequence, and reverse primers are listed immediately after the end of the nucleotide sequence. The primers used for RT-PCR analysis of gene expression are highlighted green for the A homoeolog of G. hirsutum, while the D homoeolog primers are highlighted in blue. The names of all RT-PCR primers are listed above the highlighted sequence, and the arrows indicate whether they are forward or reverse primers. The nucleotide sequences highlighted for all reverse primers correspond to their forward sequence positions. The actual nucleotide sequence of all primers, listed 5’ to 3’, is provided in Additional files 1 and 2. [file 12870_2014_312_MOESM9_ESM.pdf]

```
GheFAD3-2.1A C26F  AAGGTTCGAAAGGAGTGGAGATAAAGCAACCATTGGTCACCTATCTCATCCTAGATACAT 60
GhiFAD3-2.1A       ------------------------------------------------------------
GraFAD3-2.1D C26F  AAGGTTCGAAAGGAGTGGAGATAAAGCAACCATTGGTCACCTATCTCATCCTAGATACAT
GhiFAD3-2.1D       ------------------------------------------------------------
GheFAD3-2.2A C26F  AAGGTTCGAAAGGAGTGGAGATAAAGCAACCATTGGTCACCTATCTCATCCTAGATACAT 60
GhiFAD3-2.2A       ------------------------------------------------------------

                                     ------S44F------>
GheFAD3-2.1A       GATTTGAAACAATGGAGTTGAAAAGGGAGTATGTTAATGGAATGAATGAGCAAGGTGATT 120
GhiFAD3-2.1A C29F    AAAGAGCTCATGGAGTTGAAAAGGGAGTATGTTAATGGAATGAATGAGCAAGGTGATT 58
GraFAD3-2.1D       GATTTGAAACAATGGAGTTGAAAAGGGAGTATGCCAATGGAATGAATGAGCAAGGTGATT 120
GhiFAD3-2.1D C29F    AAAGAGCTCATGGAGTTGAAAAGGGAGTATGCCAATGGAATGAATGAGCAAGGTGATT 58
GheFAD3-2.2A       GATTTGAAACAATGGAGTTGAAAAGGGTGTATGTTAATGGAATGAATGAGCAAGGTGATT 120
GhiFAD3-2.2A C29F    AAAGAGCTCATGGAGTTGAAAAGGGAGTATGTTAATGGAATGAATGAGCAAGGTGATT 58
                        **    **************** *****  *************************
                                  --------S9F-------->
GheFAD3-2.1A       TTGATCCGAGTTCACCTCCTCCATTCCGGATTGCGGATATCCGAGCTGCCATACCGAAGC 180
GhiFAD3-2.1A       TTGATCCGAGTTCACCTCCTCCATTCCGGATTGCGGATATCCGAGCTGCCATACCGAAGC 118
GraFAD3-2.1D       TTGATCCGAGTTCACCTCCTCCATTCCGGATTGCGGATATCCGAGCTGCCATACCGAAGC 180
GhiFAD3-2.1D       TTGATCCGAGTTCACCTCCTCCATTCCGGATTGCGGATATCCGAGCTGCCATACCGAAGC 118
GheFAD3-2.2A       TTGATCCGAGTTCACCTCCTCAATTCCGGATTGCCGATATCCGAGCTGCCATACCGAAGC 180
GhiFAD3-2.2A       TTGATCCGAGTTCACCTCCTCAATTCCGGATTGCCGATATCCGAGCTGCCATACCGAAGC 118
                   ********************* ************ *************************

GheFAD3-2.1A       AGTGTTGGATCAAGAATCCATGGAGGTCAATGAGTTATGTTTTTAGGGATGTCAGTGTGG 240
GhiFAD3-2.1A       AGTGTTGGATCAAGAATCCATGGAGGTCAATGAGTTATGTTTTTAGGGATGTCAGTGTGG 178
GraFAD3-2.1D       AGTGTTGGATCAAGAATCCATGGAGGTCAATGAGTTATGTTTTTAGGGATGTCAGTGTGG 240
GhiFAD3-2.1D       AGTGTTGGATCAAGAATCCATGGAGGTCAATGAGTTATGTTTTTAGGGATGTCAGTGTGG 178
GheFAD3-2.2A       AGTGTTGGATCAAGAATCCATGGAGGTCAATGAGTTATGTTTTTAGGGATGTCAGTGTGG 240
GhiFAD3-2.2A       AGTGTTGGATCAAGAATCCATGGAGGTCAATGAGTTATGTTTTTAGGGATGTCAGTGTGG 178
                   ************************************************************

GheFAD3-2.1A       TTTTTGCATTAGCAGCAGCTGCAGTCTACTGTAATAATTGGCTGTTTTGGCCAATCTACT 300
GhiFAD3-2.1A       TTTTTGCATTAGCAGCAGCTGCAGTCTACTGCAATAATTGGCTGTTTTGGCCAATCTACT 238
GraFAD3-2.1D       TTTTTGCATTAGCAGCAGCTGCAGTCTACTGCAATAGTTGGTTGTTTTGGCCAATCTACT 300
GhiFAD3-2.1D       TTTTTGCATTAGCAGCAGCTGCAGTCTACTGCAATAGTTGGTTGTTTTGGCCAATCTACT 238
GheFAD3-2.2A       TTTTTTCTTTAGCAGCAGCTGCAGTCTACTGCAATAGTTAGTTGTTTTGGCCAATCTACT 300
GhiFAD3-2.2A       TTTTTTCTTTAGCAGCAGCTGCAGTCTACTGCAATAGTTAGTTGTTTTGGCCAATCTACT 238
                   ***** * ********************** **** ** * *******************

GheFAD3-2.1A       GGTTTGCCCAAGGAACCATGTTTTGGGCTGTCTTTGTCCTTGGACATGATTGGTAATAGT 360
GhiFAD3-2.1A       GGTTTGCCCAAGGAACCATGTTTTGGGCTGTCTTTGTCCTTGGACATGATTGGTAATAGT 298
GraFAD3-2.1D       GGTTTGCCCAAGGAACCATGTTTTGGGCTGTCTTTGTCCTTGGACATGATTGGTAATAGT 360
GhiFAD3-2.1D       GGTTTGCCCAAGGAACCATGTTTTGGGCTGTCTTTGTCCTTGGACATGATTGGTAATAGT 298
GheFAD3-2.2A       GGTTTGCCCAAGGAACCATGTTTTGGGCTGTCTTTGTCCTTGGACATGATTGGTAATAGT 360
GhiFAD3-2.2A       GGTTTGCCCAAGGAACCATGTTTTGGGCTGTCTTTGTCCTTGGACATGATTGGTAATAGT 298
                   ************************************************************

GheFAD3-2.1A       ACACTTCATTTCCTTTTTCTTGTTTTTAGTATAACTCTTTATCTTAGTTTTGGATTTGCA 420
GhiFAD3-2.1A       ACACTTCATTTCCTTTTTCTTGTTTTTAGTATAACTCTTTATCTTAGTTTTGGATTTGCA 358
GraFAD3-2.1D       GCACTTCATTTCCTTTTTCTTGTTTTTAGTATAACTCTTTATCTTAGTTTTGGATTTGCA 420
GhiFAD3-2.1D       GCACTTCATTTCCTTTTTCTTGTTTTTAGTATAACTCTTTATCTTAGTTTTGGATTTGCA 358
GheFAD3-2.2A       ACACTTCATTTCCTTTTTCTTGTTTTTAATATAACTCTTTATCTTAGTTTTGGATTTGCA 420
GhiFAD3-2.2A       ACACTTCATTTCCTTTTTCTTGTTTTTAATATAACTCTTTATCTTAGTTTTGGATTTGCA 358
                    *************************** *******************************

GheFAD3-2.1A       GTGGCCATGGAAGCTTTTCAGACAATGCTATTTTGAATAGTGTGGTGGGACATATCTTAC 480
GhiFAD3-2.1A       GTGGCCATGGAAGCTTTTCAGACAATGCTATTTTGAATAGTGTGGTGGGACATATCTTAC 418
GraFAD3-2.1D       GTGGCCATGGAAGCTTTTCAGACAATGCTATTTTGAATAGTGTGGTGGGACATATCTTAC 480
GhiFAD3-2.1D       GTGGCCATGGAAGCTTTTCAGACAATGCTATTTTGAATAGTGTGGTGGGACATATCTTAC 418
GheFAD3-2.2A       GTGGCCATGGAAGCTTTTCAGACAATGCTATTTTGAATAGTGTGGTGGGACATATCTTAC 480
GhiFAD3-2.2A       GTGGCCATGGAAGCTTTTCAGACAATGCAATTTTGAATAGTGTGGTGGGACATATCTTAC 418
                   **************************** *******************************

GheFAD3-2.1A       ATTCTTCAGTTCTTGTTCCTTACCATGGATGGTGAGTTCAATCAGGTAAATATTTGTTCT 540
GhiFAD3-2.1A       ATTCTTCAGTTCTTGTTCCTTACCATGGATGGTGAGTTCAATCAGGTAAATATTTGTTCT 478
GraFAD3-2.1D       ATTCTTCAATTCTTGTTCCTTACCATGGATGGTAAGTTCAATCAGGTAAATATTTGTTCT 540
GhiFAD3-2.1D       ATTCTTCAATTCTTGTTCCTTACCATGGATGGTAAGTTCAATCAGGTAAATATTTGTTCT 478
GheFAD3-2.2A       ATTCTTCAATTCTTGTTCCTTACCATGGATGGTAAGTTCAATCAGGTAAATATTTGTTCT 540
GhiFAD3-2.2A       ATTCTTCAATTCTTGTTCCTTACCATGGATGGTAAGTTCAATCAGGTAAATATTTGTTCT 478
                   ******** ************************ **************************
```

```
GheFAD3-2.1A      TTATTTTGTATGCTTGCAACTACATAACAGAATCTGAAAATCTTACAGGAGAATAAGCCA 600
GhiFAD3-2.1A      TTATTTTGTATGCTTGCAACTACATAACAGAATCTGAAAATCTTACAGGAGAATAAGCCA 538
GraFAD3-2.1D      TTATTTTGTATGCTTGCAACTACATAACAGAATCTGAAAATCTTACAGGAGAATAAGCCA 600
GhiFAD3-2.1D      TTATTTTGTATGCTTGCAACTACATAACAGAATCTGAAAATCTTACAGGAGAATAAGCCA 538
GheFAD3-2.2A      TTATTTTGTATGATTGCAACTACATAACATAATCTGAAAATCTTACAGGAGAATAAGCCA 600
GhiFAD3-2.2A      TTATTTTGTATGATTGCAACTACATAACATAATCTGAAAATCTTACAGGAGAATAAGCCA 538
                  ************ **************** ******************************
                                 ---------S5F--------->              <---
GheFAD3-2.1A      TAGAACTCATCATCAGAACCACGGAAATGTCGAAAATGATGAGTCATGGGTTCCGGTACA 660
GhiFAD3-2.1A      TAGAACTCATCATCAGAACCACGGAAATGTCGAAAATGATGAGTCATGGGTTCCGGTAAA 598
GraFAD3-2.1D      CAGAACTCATCATCAGAACCACGGAAATGTCGAAAAGGATGAGTCATGGGTTCCGGTACA 660
GhiFAD3-2.1D      CAGAACTCATCATCAGAACCACGGAAATGTCGAAAAGGATGAGTCATGGGTTCCGGTACA 598
GheFAD3-2.2A      TAGAACTCATTATCAGAACCACGGAAATGTCGAAAAGGAAGAGTCATGGGTGCCGGTACA 660
GhiFAD3-2.2A      TAGAACTCATCATCAGAACCACGGAAATGTCGAAAAGGATGAGTCATGGGTGCCGGTACA 598
                   ********* ************************* ** *********** ****** *

GheFAD3-2.1A      ACATTTTCGACCTTATTTTTCTTATAATTCATCCATGCTATATATATCAAGTTGCAGACT 720
GhiFAD3-2.1A      ACATTTTCGACCTTATTTTTCTTATAATTCATCCATGCTATATATATCAAGTTGCAGACT 658
GraFAD3-2.1D      ACATTTTCGACCTTATTTTTCTTATAATTCATCCATGCTATATATATCAGGTTGCAGACT 720
GhiFAD3-2.1D      ACATTTTCGACCTTATTTTTCTTATAATTCATCCATGCTATATATATCAGGTTGCAGACT 658
GheFAD3-2.2A      ACATTTTCGACCTTATTTTTCTTATAATTCATCCGTGCTATATATATCAAGTTCCAGACT 720
GhiFAD3-2.2A      ACATTTTCGACCTTATTTTTCTTATAATTCATCCGTGCTATATATATCAAGTTCCAGACT 658
                  ********************************** ************** *** ******
                                                 -----S10R---------
GheFAD3-2.1A      AACCCTTCATGCTTTTGGTTTTTAATCTCAGTTGCCTGAGAATATTTACAAGAAACTCGA 780
GhiFAD3-2.1A      AACCCTTCATGCTTTTGGTTTTTAATCTCAGTTGCCTGAGAATATTTACAAGAAACTCGA 718
GraFAD3-2.1D      AACCCTTCATACTTTTGGTTTTTAATCTCAGATGCCTGAGAATATTTACAAGAAACTCGA 780
GhiFAD3-2.1D      AACCCTTCATACTTTTGGTTTTTAATCTCAGATGCCTGAGAATATTTACAAGAAACTCGA 718
GheFAD3-2.2A      AACCCTTCATACTTTTGGTTTTTAATCTCAGTTGCCTGAGAATATTTACAAGAAACTCGA 780
GhiFAD3-2.2A      AACCCTTCATACTTTTGGTTTTTAATCTCAGTTGCCTGAGAATATTTACAAGAAACTCGA 718
                  ********** ******************** ****************************

GheFAD3-2.1A      CACCAGTACGCGATTACTGCGATTCACCGTCCCCTTTCCCCTATTTGCATTCCCCGTGTA 840
GhiFAD3-2.1A      CACCAGTACGCGATTACTGCGATTCACCGTCCCCTTTCCCCTATTTGCATTCCCCGTGTA 778
GraFAD3-2.1D      CACCAGTACGCGATTACTGCGATTCACCGTCCCCTTTCCCCTATTTGCATTCCCCGTGTA 840
GhiFAD3-2.1D      CACCAGTACGCGATTACTGCGATTCACCGTCCCCTTTCCCCTATTTGCATTCCCCGTGTA 778
GheFAD3-2.2A      CACCAGTACGCGATTACTGCGATTCATCGTCCCCTTTCTCCTATTTGCATTCCCCATGTA 840
GhiFAD3-2.2A      CACCAGTACGCCATTACTGCGATTCACCGTCCCCTTTCTCCTATTTGCAATTCCCATGTA 778
                  *********** ************** *********** ********** * *** ****

GheFAD3-2.1A      TTTGGTGAGAAAGCTTGTTTTGTTTTAGTCGACAAAAAGATTAACATAATGGCTGGTAAG 900
GhiFAD3-2.1A      TTTGGTGAGAAAGCTTGTTTTGTTTTAGTCGACAAAAAGATTAACATAATGGCTGGTAAG 838
GraFAD3-2.1D      TTTGGTGGGAAAGCTTGTTTTGTTTTAGTCGACAAAAAGATTAACATAATGGCTAGTAAG 900
GhiFAD3-2.1D      TTTGGTGGGAAAGCTTGTTTTGTTTTAGTCGACAAAAAGATTAACATAATGGCTAGTAAG 838
GheFAD3-2.2A      TTTGGTGAGAAAGCTTGTTTTGTTT-------------------------GGCTAGTAAG 875
GhiFAD3-2.2A      TTTGGTGAGAAAGCTTGTTTTGTTT-------------------------GGCTAGTAAG 813
                  ******* *****************                         **** *****

GheFAD3-2.1A      GAGTTAAATTTGGCTATCTTTTGTTTTCATTACAGTGGTATAGAAGCCCAGGCAAAGAAG 960
GhiFAD3-2.1A      GAGTTAAATTTGGCTATCTTTTGTTTTCATTACAGTGGTATAGAAGCCCAGGCAAAGAAG 898
GraFAD3-2.1D      GAGTTAAATTTGGCTATCTTTTGTTTTCATTACAGTGGTATAGAAGCCCAGGCAAAGAAG 960
GhiFAD3-2.1D      GAGTTAAATTTGGCTATCTTTTGTTTTCATTACAGTGGTATAGAAGCCCAGGCAAAGAAG 898
GheFAD3-2.2A      GAGTTAAATTTGGCTAT-TTTTGTTTTCATTACAGTGGTTTAGAAGCCCAGGCAAAGAAG 934
GhiFAD3-2.2A      GAGTTAAATTTGGCTAT-TTTTGTTTTCATTACAGTGGTTTAGAAGCCCAGGCAAAGAAG 872
                  ***************** ********************* ********************
                               <-----S45R----------
GheFAD3-2.1A      GATCTCATTTCAACCCTTACAGCAACTTGTTTAGCCCCCAAGAGAGGAAACTTGTGATGA 1020
GhiFAD3-2.1A      GATCTCATTTCAACCCTTACAGCAACTTGTTTAGCCCCCAAGAGAGGAAACTTGTGATGA 958
GraFAD3-2.1D      GATCTCATTTCAACCCTTACAGCAACTTGTTTAGCCCCCAAGAGAGGAAACTTGTGATGA 1020
GhiFAD3-2.1D      GATCTCATTTCAATCCTTACAGCAACTTGTTTAGCCCCCAAGAGAGGAAACTTGTGATGA 958
GheFAD3-2.2A      GATCTCATTTCAACCCTTACAGCAACTTGTTTAGCCCCCAAGAGAGGAAACTTGTGATGA 994
GhiFAD3-2.2A      GATCTCATTTCAACCCTTACAGCAACTTGTTTAGCCCCCAAGAGAGGAAACTTGTGATGA 932
                  ************* **********************************************

GheFAD3-2.1A      TATCCACTGCCTGTTGGTCTACAATGGCTATTATACTTGCCTACGTATCCTTTACAGTTG 1080
GhiFAD3-2.1A      TATCCACTGCCTGTTGGTCTACAATGGCTATTATACTTGCCTACGTATCCTTTACAGTTG 1018
GraFAD3-2.1D      TATCCACTGCCTGTTGGTCTACAATGGCTATTATACTTGCCTACGTATCGTTTACAGTTG 1080
GhiFAD3-2.1D      TATCCACTGCCTGTTGGTCTACAATGGCTATTATACTTGCCTACGTATCGTTTACAGTTG 1018
GheFAD3-2.2A      TATCCAATGCCTGTTGGTCTCCAATGGTTATTATACTTGCCTTCGTATCTTTTGCAGTTG 1054
GhiFAD3-2.2A      TATCCAATGCCTGTTGGTCTCCAATGGTTATTATACTTGCCTACGTATCTTTTACAGTTG 992
                  ****** ************* ****** ************** ****** *** ******
```

```
GheFAD3-2.1A      GTCCATCCCACATGTTTAAGCTCTATGGCATTCCCTACTTGGTACGTCAAGCTGTGTGAT 1140
GhiFAD3-2.1A      GTCCATCCCACATGTTTAAGCTCTATGGCATTCCCTACTTGGTACGTCAAGCTGTGTGAT 1078
GraFAD3-2.1D      GTCCATCCCACATGTTTAAGCTCTATGGCATTCCCTACTTGGTACGTCAAGTTGTGTGAG 1140
GhiFAD3-2.1D      GTCCATCCCACATGTTTAAGCTCTATGGCATTCCCTACTTGGTACGTCAAGTTGTGTGAG 1078
GheFAD3-2.2A      GTCCATCCCACGTGTTTAAGCTCTATGGCATTCCCTATTTGGTACGTCAAGTTGTGTGAG 1114
GhiFAD3-2.2A      GTCCATCCCACGTGTTTAAGCTCTATGGCATTCCCTATTTGGTACGTCAAGTTGTGTGAG 1052
                  *********** ************************* ************* ******* 

GheFAD3-2.1A      TCACTTGCGCT-AAGTTTCGATCCATCAAGCTGAGCCATTTTATTCTGGTTTCGTAGATT 1199
GhiFAD3-2.1A      TCACTTGCGCT-AAGTTTCGATCCATCAAGCTGAGCCATTTTATTCTGGTTTCGTAGATT 1137
GraFAD3-2.1D      TCACTTGCGCT-AAGTTTCGATCCATCAAGCTGAGCCATTTTATTCTGGTTTCGTAGATT 1199
GhiFAD3-2.1D      TCACTTGCGCT-AAGTTTCGATCCATCAAGCTGAGCCATTTTATTCTGGTTTCGTAGATT 1137
GheFAD3-2.2A      TCACTTGCGCTTAAGTTTCGATCGATCAAGCTGAGCCATTTTATTCTGGTTTCGTAGATT 1174
GhiFAD3-2.2A      TCACTTGCGCTTAAGTTTCGATCGATCAAGCTGAGCCATTTTATTCTGGTTTCGTAGATT 1112
                  *********** *********** ************************************

GheFAD3-2.1A      TTCGTGATATGGCTAGATTTAGTGACTTACCTACATCACCATGGCCATGAGCAGAAACTA 1259
GhiFAD3-2.1A      TTCGTGATATGGCTAGATTTAGTGACTTACCTACATCACCATGGCCATGAGCAGAAACTA 1197
GraFAD3-2.1D      TTCGTGATATGGCTAGATTTAGTAACTTACCTACATCACCATGGCCATGAGCAGAAACTA 1259
GhiFAD3-2.1D      TTCGTGATATGGCTAGATTTAGTAACTTACCTACATCACCATGGCCATGAGCAGAAACTA 1197
GheFAD3-2.2A      TTCGTGGTATGGCTAGATTTATTGACTTATCTACATCACCATGGCCATGAGCACAAACTA 1234
GhiFAD3-2.2A      TTCGTGATATGGCTAGATTTATTGACTTATCTACATCACCATGGCCATGAGCACAAACTA 1172
                  ****** ************** * ***** *********************** ******

GheFAD3-2.1A      CCTTGGTACCGTGGCAAGGTA-CCTACAATTTAACTTTGTATGTACGCATTTGAGAGTAA 1318
GhiFAD3-2.1A      CCTTGGTACCGTGGCAAGGTA-CCTACAATTTAACTTTGTATGTACGCATTTGAGAGTAA 1256
GraFAD3-2.1D      CCTTGGTACCGTGGCAAGGTAACCTACAATTTAACTTTGTATGTACGCATTTGAGAGTAA 1319
GhiFAD3-2.1D      CCTTGGTACCGTGGCAAGGTAACCTACAATTTAACTTTGTATGTACGCATTTGAGAGTAA 1257
GheFAD3-2.2A      CCTTGGTACCGTGGCAAGGTAACCTACAATTTAACTTTGTATGTAGGCATTTAAGAGTAA 1294
GhiFAD3-2.2A      CCTTGGTACCGTGGCAAGGTAACCTACAATTTAACTTTGTATGTAGGCATTTAAGAGTAA 1232
                  ********************* *********************** ****** *******

GheFAD3-2.1A      AAAGTTGTTCAAACTTGAGAGGTTT-GTTCATTTACATAACAGGAATGGAGTTATCTGAG 1377
GhiFAD3-2.1A      AAAGTTGTTCAAACTTGAGAGGTTT-GTTCATTTACATGACAGGAATGGAGTTATCTGAG 1315
GraFAD3-2.1D      AAAATTGTTC--ACTTGAGAGGTTTTGTTCATTTACATGACAGGAATGGAGTTATCTCAG 1377
GhiFAD3-2.1D      AAAATTGTTC--ACTTGAGAGGTTTTGTTCATTTACATGACAGGAATGGAGTTATCTCAG 1315
GheFAD3-2.2A      AAAATTGTTCAAACTTGAGAGGTTTTTTTCATTTACATGACAGGAATGGAATTATCTCAG 1354
GhiFAD3-2.2A      AAAATTGTTCAAACTTGAGAGGTTTTTTTCATTTACATGACAGGAATGGAGTTATCTCAG 1292
                  *** ******  *************  *********** *********** ****** **

GheFAD3-2.1A      AGGAGGGCTTACAACAGTGGATCGTGATTATGGTTTATTCAATAATATCCACCATGACAT 1437
GhiFAD3-2.1A      AGGAGGGCTTACAACAGTGGATCGTGATTATGGTTTATTCAATAATATCCACCATGACAT 1375
GraFAD3-2.1D      AGGAGGGCTTACAACAGTGGATCGTGATTATGGTTTATTCAATAATATCCACCATGACAT 1437
GhiFAD3-2.1D      AGGAGGGCTTACAACAGTGGATCGTGATTATGGTTTATTCAATAATATCCACCATGACAT 1375
GheFAD3-2.2A      AGGAGGGCTTACAACAGTGGATCTTGATTATGGTTTATTCAATAATATCCACCATGACAT 1414
GhiFAD3-2.2A      AGGAGGGCTTACAACAGTGGATCTTGATTATGGTTTATTCAATAATATCCACCATGACAT 1352
                  *********************** ************************************

GheFAD3-2.1A      TGGCACACATGTCATCCATCATCTCTTCCCTCAAATCCCTCATTATCACTTAGTGGAAGC 1497
GhiFAD3-2.1A      TGGCACACATGTCATCCATCATCTCTTCCCTCAAATCCCTCATTATCACTTAGTGGAAGC 1435
GraFAD3-2.1D      TGGCACACATGTCATCCATCATCTCTTCCCTCAAATCCCTCATTATCACTTAGTGGAAGC 1497
GhiFAD3-2.1D      TGGCACACATGTCATCCATCATCTCTTCCCTCAAATCCCTCATTATCACTTAGTGGAAGC 1435
GheFAD3-2.2A      TGGCACACATGTCATCCATCATCTCTTCCCTCAAATCCCTCATTATCACTTAGTGGAAGC 1474
GhiFAD3-2.2A      TGGCACACATGTCATCCATCATCTCTTCCCTCAAATCCCTCATTATCACTTAGTGGAAGC 1412
                  ************************************************************

GheFAD3-2.1A      TGTAAGCAAAATATTCTTTAGAAACAATTAAACCGATTGATTTCATAAGATAAAATGAAG 1557
GhiFAD3-2.1A      TGTAAGCAAAATATTCTTTAGAAACAATTAAACCGATTGATTTCATAAGATAAAATGAAG 1495
GraFAD3-2.1D      TGTATGTAGAGTATTCTTTAGAAACAATTAAACCGATTGATTTCATAAGATAAAATGAAG 1557
GhiFAD3-2.1D      TGTATGTAGAGTATTCTTTAGAAACAATTAAACCGATTGATTTCATAAGATAAAATGAAG 1495
GheFAD3-2.2A      TGTATGTAAAATATTCTTTAGAAACAATTAAACCGATTGATTTCATAAGATAAAATGAAG 1534
GhiFAD3-2.2A      TGTATGTAAAATATTCTTTAGAAACAATTAAACCGATTGATTTCATAAGATAAAATGAAG 1472
                  **** * * * *************************************************

GheFAD3-2.1A      G--GTGTTTTCTGGGTTTTGCAGACTAAAGCGGCAAAACCGGTGATAGGAAAATTCTACC 1615
GhiFAD3-2.1A      G--GTGTTTTCTGGGTTTTGCAGACTAAAGCGGCAAAACCGGTGATAGGAAAATTCTACC 1553
GraFAD3-2.1D      G--GTGTTTTCTGGATTTTGCAGACTAAAGCGGCAAAACCGGTGATAGGAAAATTCTATT 1615
GhiFAD3-2.1D      G--GTGTTTTCTGGATTTTGCAGACTAAAGCGGCAAAACCGGTGATAGGAAAATTCTATT 1553
GheFAD3-2.2A      GCCGTGTTTTCTGGGTTTTGCAGACTAAAGCGGCAAAACCGGTTATAGGAAAATTCTATC 1594
GhiFAD3-2.2A      GCCGTGTTTTCTGGGTTTTGCAGACTAAAGCGGCAAAACCGGTTATAGGAAACTTCTATC 1532
                  *  *********** **************************** ******** *****  
```

```
GheFAD3-2.1A        GGGAGCCGAAGAAATCAGGGGCTGTTCCATTTCATTTGATCAATAATTTAATCACAAGCC 1675
GhiFAD3-2.1A        GGGAGCCGAAGAAATCAGGGGCTGTTCCATTTCATTTGATCAATAATTTAATCACAAGCC 1613
GraFAD3-2.1D        GGGAGCCGAAGAAATCAGGTGCTGTTCCATTTCATTTGATCAAGAATTTAATCACAAGCC 1675
GhiFAD3-2.1D        GGGAGCCGAAGAAATCAGGTGCTGTTCCATTTCATTTGATCAAGAATTTAATCACAAGCC 1613
GheFAD3-2.2A        GGGAGCCGAAGAAATCAGGGGCTGTTCCATTTAATTTGATCAATAATTTAATCACAAGCC 1654
GhiFAD3-2.2A        GGGAGCCGAAGAAATCAGGGGCTGTTCCATTTAATTTGATCAATAATTTAATCACAAGCC 1592
                    ******************* ************ ********** ****************

                                                                      <----S6R--
GheFAD3-2.1A        TCGCACAAGATCATTACGTCAGCAATAGTGGAGATATA--GTGTTTCATCGGACTGATCT 1733
GhiFAD3-2.1A        TCGCACAAGATCATTACGTCAGCAATAGTGGAGATATA--GTGTTTTATCGGACTGATCT 1671
GraFAD3-2.1D        TCGCACAAGATCATTACGTCAGCAATAGTGGAGATATA--GTGTTTTATCAGACTGATCT 1733
GhiFAD3-2.1D        TCGCACAAGATCATTACGTCAGCAATAGTGGAGATATA--GTGTTTTATCAGACTGATCT 1671
GheFAD3-2.2A        TCGGACAAGATCATTACGTCAGCAATAGTGGAGATATATAGTGTTCTATCAGACTGATCT 1714
GhiFAD3-2.2A        TCGGACAAGATCATTACGTCAGCAATAGTGGAGATATATAGTGTTCTATCAGACTGATCT 1652
                    *** **********************************  *****  *** *********
                    -----------------
GheFAD3-2.1A        AAACCTGTTTAAGTTGCTTTCCCTTGCCAAATCTGAATGATACAATAACTAAGATTAAGG 1793
GhiFAD3-2.1A        AAACCTGTTTAAGTTGCTTTCCCTTGCCAAATCTGAATGATACAATAACTAAGATGCATG 1731
GraFAD3-2.1D        AAACCTGTTTCAGTTGCTTTCCCTTGCCAAATATGAATGATACAATAACTAAGATTAAGG 1793
GhiFAD3-2.1D        AAACCTGTTTCAGTTGCTTTCCCTTGCCAAATATGAATGATACAATAACTAAGATGCATG 1731
GheFAD3-2.2A        AAACCTGTTTCAGTTGCTTTCCCATGCCAAATCTGAATGATACAATAACTAAGATTAAGG 1774
GhiFAD3-2.2A        AAACCTGTTTCAGTTGCTTTCCCATGCCAAATCTGAATGATACAATAACTAAGATGCATG 1712
                    ********** ************ ******** **********************  * *

GheFAD3-2.1A        GCCATAATTTGTTCAT C28R 1809
GhiFAD3-2.1A        CCCC------------ C31R 1735
GraFAD3-2.1D        GCCATAATTTGTTCAT C28R 1809
GhiFAD3-2.1D        CCCC------------ C31R 1735
GheFAD3-2.2A        GCCATAATTTGTTCAT C28R 1790
GhiFAD3-2.2A        CCCC------------ C31R 1716
                     **
```
