# Supplementary material for: Genome-wide analysis of the omega-3 fatty acid desaturase gene family in Gossypium
Source: BMC Plant Biol. 2014 Nov 18;14:312. doi: 10.1186/s12870-014-0312-5 (PMC4245742; doi:10.1186/s12870-014-0312-5)
Supplement: Additional file 10: — RNA-seq analysis of omega-3 FAD gene expression in cotton fiber, seeds, petals and leaves. (A) Fibers were harvested from the indicated cotton varieties at 10 and 20 DPA, which represents primary and secondary cell wall synthesis, respectively, then RNA-seq analysis was performed as described [56]. Cotton varieties are indicated along the bottom. (B) Developing seeds were harvested from the indicated cotton varieties at 10, 20, 30, or 40 DPA then RNA-seq analysis was performed as described. Similar RNA-seq analyses were performed on cotton petals (C) and leaves (D), from the indicated plant lines [59,60]. Transcripts were quantified as “reads per kilobase per million mapped reads” (RPKM). For simplicity, data for A and D homoeologous sequences were combined. Values represent average and standard deviation of three biological replicates. For data presented in panels (C) and (D), student’s t-test was used for comparison of FAD7/8-1 to FAD7/8-2, and * denotes p <0.05. [file 12870_2014_312_MOESM10_ESM.pdf]

```
GheFAD78-1A      CGGTGGTTGAGTTTTTCTTTTTACCTGAGTCTTCAATGGCGGGTTTCATTATATCTGGTT 60
GhiFAD78-1A C27F  GGTGGTTGAGTTTTTCTTTTTACCTGAGTCTTCAATGGCGGGTTTCATTATATCTGGTT 59
GraFAD78-1D C13F CGGTGGTTGAGTTTTTCTTTTTATCAGAGTCTTCAATGGCGGGTTTCGTTATATCTGGTT 60
GhiFAD78-1D C15F         CCCGAGCTCTTTTTATCAGAGTCTTCAATGGCGGGTTTCGTTATATCTGGTT 52
                                 ******* * ********************* ************

GheFAD78-1A      TAAAGCCTCTTCCTTGTATCTACGATAGACCCGTCGCTGGCGTTATCTCGAGGAGTTCTT 120
GhiFAD78-1A      TAAAGCCTCTTCCTTGTATCTACGATAGACCCGTCGCTGGCGTTATCTCGAGGAGTTCTT 119
GraFAD78-1D      TAAAGCCTCTTCCTTGTATCTACGATAGACCCGCCGCTGGTGTTATCTCGACGAGTTCTT 120
GhiFAD78-1D      TAAAGCCTCTTCCTTGTATCTACGATAGACCCGCCGCTGGTGTTATCTCGACGAGTTCTT 112
                 ********************************* ****** ********** ********

GheFAD78-1A      CAAAATCCAGATTTTTAACCAAAAACAAGAATTTCCCAGATCTAAAAATATTAAATCCAA 180
GhiFAD78-1A      CAAAATCCAGATTTTTAACCAAAAACAAGAATTTCCCAGATCTAAAAATATTAAATCCAA 179
GraFAD78-1D      CAAAATCCAGATTTTTAACCAAAAACAAGAATTTCCCAGGTCTAAAAACGTTAAATCCAA 180
GhiFAD78-1D      CAAAATCCAGATTTTTAACCAAAAACAAGAATTTCCCAGGTCTAAAAACGTTAAATCCAA 172
                 *************************************** ********  **********

GheFAD78-1A      TCAAATCCAGGAACTGGGGTCTAAACGTGAGTGCCCCATTTAGAGTTGCATCCGTTGAAG 240
GhiFAD78-1A      TCAAATCCAGGAACTGGGGTCTAAACGTGAGTGCCCCATTTAGAGTTGCATCCGTTGAAG 239
GraFAD78-1D      TCAAATCCAGGAACTGGGGTCTAAACGTGAGTGCCCCATTTAGAGTTGCATCCGTTGAAG 240
GhiFAD78-1D      TCAAATCCAGGAACTGGGGTCTAAACGTGAGTGCCCCATTTAGAGTTGCATCCGTTGAAG 232
                 ************************************************************

GheFAD78-1A      AAGATGAGGGAAGGAAAGAGAGGAACCATGGTATTAATGGATTTGAGGAACAAGAACAAG 300
GhiFAD78-1A      AAGATGAGGGAAGGAAAGAGAGGAACCATGGTATTAATGGATTTGAGGAACAAGAACAAG 299
GraFAD78-1D      AAGATGAGGGAAGGAAAGAGAGAAACCATGGTATTAATGGATTTGAGGAACAAGAACAAG 300
GhiFAD78-1D      AAGATGAGGGAAGGAAAGAGAGAAACCATGGTATTAATGGATTTGAGGAACAAGAACAAG 292
                 ********************** *************************************

GheFAD78-1A      AGGCAGGGTTCGACCCTGGGGCGCCTCCGCCGTTTAAGTTGGCTGATATAAGAGCGGCCA 360
GhiFAD78-1A      AGGCAGGGTTCGACCCTGGGGCGCCTCCGCCGTTTAAGTTGGCTGATATAAGAGCGGCCA 359
GraFAD78-1D      AGGCAGGGTTCGACCCTGGGGCGCCTCCGCCGTTTAAGCTGGCTGATATAAGAGCGGCCA 360
GhiFAD78-1D      AGGCAGGGTTCGACCCTGGGGCGCCTCCGCCGTTTAAGCTGGCTGATATAAGAGCGGCCA 352
                 ************************************** *********************

GheFAD78-1A      TACCGAAGCATTGTTGGGTGAAGGATCCATGGAAATCTATGAGCTACGTGGTGAGGGATG 420
GhiFAD78-1A      TACCGAAGCATTGTTGGGTGAAGGATCCATGGAAATCTATGAGCTACGTGGTGAGGGATG 419
GraFAD78-1D      TACCGAAGCATTGTTGGGTGAAGGATCCATGGAAATCTATGAGCTACGTGGTGAGGGATG 420
GhiFAD78-1D      TACCGAAGCATTGTTGGGTGAAGGATCCATGGAAATCTATGAGCTACGTGGTGAGGGATG 412
                 ************************************************************

GheFAD78-1A      TCGCTGTGGTGTTAGGCCTGGCGGCTGCTGCGGTCTATGTTAACAACTGGATTGTTTGGC 480
GhiFAD78-1A      TCGCTGTGGTGTTAGGCCTGGCGGCTGCTGCGGTCTATGTTAACAACTGGATTGTTTGGC 479
GraFAD78-1D      TCGCTGTGGTGTTAGGCCTTGCGGCTGCTGCGGTCTATGTTAACAACTGGATTGTTTGGC 480
GhiFAD78-1D      TCGCTGTGGTGTTAGGCCTTGCGGCTGCTGCGGTCTATGTTAACAACTGGATTGTTTGGC 472
                 ******************* ****************************************

GheFAD78-1A      CTCTTTACTGGGCTGCTCAAGGAACCATGTTTTGGGCTCTTTTTGTTCTTGGTCATGACT 540
GhiFAD78-1A      CTCTTTACTGGGCTGCTCAAGGAACCATGTTTTGGGCTCTTTTTGTTCTTGGTCATGACT 539
GraFAD78-1D      CTCTTTACTGGGCTGCACAAGGAACCATGTTTTGGGCTCTTTTTGTTCTTGGTCATGACT 540
GhiFAD78-1D      CTCTTTACTGGGCTGCACAAGGAACCATGTTTTGGGCTCTTTTTGTTCTTGGTCATGACT 532
                 **************** *******************************************

GheFAD78-1A      GGTAAACTTTTTTTTTTTTTT-GAATTGCATTTGCGAGTTACAACCTCCTCTGTTATTAG 599
GhiFAD78-1A      GGTAAACTTTTTTTTTTTTTTTTGATTGCATTTGCGAGTTACAACCTCCTCTGTTATTAG 599
GraFAD78-1D      GGTAAACTTTTTTTTTTTTTT-GAATTGCATTTGCGAGTTGCAACCTCCTCTGTTATTAG 599
GhiFAD78-1D      GGTAAACTTTTTTTTTTTTTT-GAATTGCATTTGCGAGTTGCAACCTCCTCTGTTATTAG 591
                 ********************* ** *************** *******************

GheFAD78-1A      ATCTGACTGTCTTAATATGTTTCATGGATTTTGTTTCATTTCTTTGTGCAGCGGCCACGG 659
GhiFAD78-1A      ATCTGACTGTCTTAATATGTTTCATGGATTTTGTTTCATTTCTTTGTGCAGCGGCCACGG 659
GraFAD78-1D      ATCTGACTGTCTTAATATGTTTCATGGATTTTGTTTCATTTCATTGTGCAGCGGCCATGG 659
GhiFAD78-1D      ATCTGACTGTCTTAATATGTTTCATGGATTTTGTTTCATTTCATTGTGCAGCGGCCATGG 651
                 ****************************************** ************** **

GheFAD78-1A      TAGCTTTTCAAACGATCCCAAGTTAAACAGTGTAGTGGGGCATCTCTTGCATTCTTCCAT 719
GhiFAD78-1A      TAGCTTTTCAAACGATCCCAAGTTAAACGGTGTAGTGGGGCATCTCTTGCATTCTTCCAT 719
GraFAD78-1D      TAGCTTTTCAAACGATCCCAAGTTAAACAGTGTAGTGGGGCATCTCTTGCATTCTTCCAT 719
GhiFAD78-1D      TAGCTTTTCAAACGATCCCAAGTTAAACAGTGTAGTGGGGCATCTCTTGCATTCTTCCAT 711
                 **************************** *******************************
```

```
GheFAD78-1A     TCTTGTGCCTTACCATGGATGGTATGCCTAGTATTTCTTGAAAGGTTCATCTCTTCTCCA 779
GhiFAD78-1A     TCTTGTGCCTTACCATGGATGGTATGCCTAGTATTTCTTGAAAGGTTCATCTCTTCTCCA 779
GraFAD78-1D     TCTTGTGCCTTACCATGGATGGTATGCCTAGTATTTTTTGAAAGGTTCATCTCTTCCCCA 779
GhiFAD78-1D     TCTTGTGCCTTACCATGGATGGTATGCCTAGTATTTTTTGAAAGGTTCATCTCTTCCCCA 771
                ************************************ ******************* ***

GheFAD78-1A     TTTGCATATGAAAAGTTTTAATTGAATTCATTTCCATTATGATTGAACTTTACAGGAGAA 839
GhiFAD78-1A     TTTGCATATGAAAAGTTTTAATTGAATTCATTTCCATTATGATTGAACTTTACAGGAGAA 839
GraFAD78-1D     TTTGTATATGAAAAGTTTAAATTGAATTCATTTCCATTATGATTGAACTTTACAGGAGAA 839
GhiFAD78-1D     TTTGTATATGAAAAGTTTAAATTGAATTCATTTCCATTATGATTGAACTTTACAGGAGAA 831
                **** ************* *****************************************

GheFAD78-1A     TTAGCCACAGGACTCACCATCAAAACCATGGTCATGTTGAGAATGATGAATCATGGCACC 899
GhiFAD78-1A     TTAGCCACAGGACTCACCATCAAAACCATGGTCATGTTGAGAATGATGAATCATGGCACC 899
GraFAD78-1D     TTAGCCACAGGACTCACCATCAAAACCATGGTCATGTTGAGAATGATGAATCATGGCACC 899
GhiFAD78-1D     TTAGCCACAGGACTCACCATCAAAACCATGGTCATGTTGAGAATGATGAATCATGGCACC 891
                ************************************************************

GheFAD78-1A     CGGTGAGTTAATTTCTCTCATCTTCTTTTTTTTCTCTTATATTGAGAATGATGAATCACG 959
GhiFAD78-1A     CGGTGAGTTAATTTCTCT---CTTCTTTTTTTTCTCTTATATTGAGAATGATGAATCACG 956
GraFAD78-1D     CGGTGAGTTAATTTCTCTCATCTTCTTTCTTTTCCCTTATATTGAGAATGATGAATCATG 959
GhiFAD78-1D     CGGTGAGTTAATTTCTCTCATCTTCTTTCTTTTCCCTTATATTGAGAATGATGAATCATG 951
                ******************   ******* ***** *********************** *

GheFAD78-1A     GCCTTTGGTCTAAATTGTAAACACTTTTGTCAATTCAATTGCAGTTATCTGAGAAGATAT 1019
GhiFAD78-1A     GCCTTTGGTCTAAATTGTAAACACTTTTGTCAATTCAATTGCAGTTATCTGAGAAGATAT 1016
GraFAD78-1D     GCCTTTGGTCTAAATTGTAAACACTTTTGTCAATTCAATTGCAGTTGTCTGAGAAGATAT 1019
GhiFAD78-1D     GCCTTTGGTCTAAATTGTAAACACTTTTGTCAATTCAATTGCAGTTGTCTGAGAAGATAT 1011
                ********************************************** *************

GheFAD78-1A     ACAGGAGTTTAGATACTCTAACACGAACATTGCGGTTCATATTGCCTTTTCCCATGCTTG 1079
GhiFAD78-1A     ACAGGAGTTTAGATACTCTAACACGAACATTGCGGTTCATATTGCCTTTTCCCATGCTTG 1076
GraFAD78-1D     ACAGGAGTTTAGATACTCTAACACGAACATTGCGGTTCATATTGCCTTTTCCCATGCTTG 1079
GhiFAD78-1D     ACAGGAGTTTAGATACTCTAACACGAACATTGCGGTTCATATTGCCTTTTCCCATGCTTG 1071
                ************************************************************

GheFAD78-1A     CATTCCCTTTCTACCTTGTAAGTGTTTTCGTGGTTATTAAATTTTTGATGATTCCTATAT 1139
GhiFAD78-1A     CATTCCCTTTCTACCTTGTAAGTGTTTTCGTGGTTATTAAATTTTTGATGATTCCTATAT 1136
GraFAD78-1D     CATTCCCTTTCTACCTTGTAAGTGTTTTCGTTGTTATTAAATTTTTGATGATTCCTATAT 1139
GhiFAD78-1D     CATTCCCTTTCTACCTTGTAAGTGTTTTCGTTGTTATTAAATTTTTGATGATTCCTATAT 1131
                ******************************* ****************************

GheFAD78-1A     GTTGAGAAATTAATGGTTGATTTCACTTTAAATTGCAGTGGAACAGAAGTCCGGGAAAGA 1199
GhiFAD78-1A     GTTGAGAAATTAATGGTTGATTTCACTTTAAATTGCAGTGGAACAGAAGTCCGGGAAAGA 1196
GraFAD78-1D     GTTGAGAAATTAATGGTTGATTTCACTTTAAATTGCAGTGGAACAGAAGTCCAGGAAAGA 1199
GhiFAD78-1D     GTTGAGAAATTAATGGTTGATTTCACTTTAAATTGCAGTGGAACAGAAGTCCAGGAAAGA 1191
                **************************************************** *******
                                           -------S32F--------->
                                                   ---------S34F---------->
GheFAD78-1A     GCGGTTCGCACTTCGACCCCAGCAGTGATTTGTTTGTCCCGACCGAACGAAAAGATGTTA 1259
GhiFAD78-1A     GCGGTTCGCACTTCGACCCCAGCAGTGATTTGTTTGTCCCGACCGAACGAAAAGATGTTA 1256
GraFAD78-1D     GTGGTTCGCACTTCGACCCCAGCAGTGATTTGTTTGTCCCGACTGAAAGAAAAGATGTGA 1259
GhiFAD78-1D     GTGGTTCGCACTTCGACCCCAGCAGTGATTTGTTTGTCCCGACTGAAAGAAAAGATGTGA 1251
                * ***************************************** *** ********** *

GheFAD78-1A     TTACTTCCACTCTATGTTGGACAGCCATGGCTGCTTTTCTTGTTGGCTTGGGTTTCACAA 1319
GhiFAD78-1A     TTACTTCCACTCTATGTTGGACAGCCATGGCTGCTTTTCTTGTTGGCTTGGGTTTCACAA 1316
GraFAD78-1D     TTACTTCCACTCTATCTTGGACAGCCATGGCTGCTATTCTTGTTGGCTTGGGTTTCACAA 1319
GhiFAD78-1D     TTACTTCCACTCTATCTTGGACAGCCATGGCTGCTATTCTTGTTGGCTTGGGTTTCACAA 1311
                *************** ******************* ************************

GheFAD78-1A     TGGGTCCTATGCAGTTGCTTAAACTATATGGCATTCCATATTGGGTAGGTTTCAAGTTTC 1379
GhiFAD78-1A     TGGGTCCTATGCAGTTGCTTAAACTATATGGCATTCCATATTGGGTAGGTTTCAAGTTTC 1376
GraFAD78-1D     TGGGTCCTATGCAGTTGCTTAAGCTATATGGCATTCCATATTGGGTAGGTTTCAAGTTTC 1379
GhiFAD78-1D     TGGGTCCTATGCAGTTGCTTAAGCTATATGGCATTCCATATTGGGTAGGTTTCAAGTTTC 1371
                ********************** *************************************
```

```
GheFAD78-1A       TTCTTTCAAGGAAACGATATCGGGAAGTAGAAAATATGATTTGACTCGTCTTTGGTTTGA 1439
GhiFAD78-1A       TTCTTTCAAGGAAACGATATCGGGAAGTAGAAAATATGATTTGACTCGTCTTTGGTTTGA 1436
GraFAD78-1D       TTCTTTCAAGGAAACGATATCGGGAAGTAGAAAATATGATTTGACTCGTCTTTGGTTTGA 1439
GhiFAD78-1D       TTCTTTCAAGGAAACGATATCGGGAAGTAGAAAATATGATTTGACTCGTCTTTGGTTTGA 1431
                  ************************************************************

GheFAD78-1A       TGTTTAGATTTTCGTGATGTGGCTGGATGGGGTTACATACTTGCATCACCATGGTCATGA 1499
GhiFAD78-1A       TGTTTAGATTTTCGTGATGTGGCTGGATGGGGTTACATACTTGCATCACCATGGTCATGA 1496
GraFAD78-1D       TGTTTAGATTTTCGTGATGTGGCTGGATGGGGTTACATACTTGCATCACCATGGTCATGA 1499
GhiFAD78-1D       TGTTTAGATTTTCGTGATGTGGCTGGATGGGGTTACATACTTGCATCACCATGGTCATGA 1491
                  ************************************************************

GheFAD78-1A       AGAGAAGCTTCCTTGGTACCGTGGGAAGGTATATGAAATTTGTTGGTCTAATTTAATATG 1559
GhiFAD78-1A       AGAGAAGCTTCCTTGGTACCGTGGGAAGGTATATGAACTTTGTTGGTCTAATTTAATATG 1556
GraFAD78-1D       AGAGAAGCTTCCTTGGTACCGTGGGAAGGTATATGAAATTTGTTGGTCTAATTTAATATG 1559
GhiFAD78-1D       AGAGAAGCTTCCTTGGTACCGTGGGAAGGTATATGAAATTTGTTGGTCTAATTTAATATG 1551
                  ************************************* **********************

GheFAD78-1A       CTAAGGCGCCTATGTTGAAATTCATATGGAAACTTACAAATAAAATAACAAATATGGATA 1619
GhiFAD78-1A       CTAAGGCGCCTATGTTGAAATTCATATGGAAACTTACAAATAAAATAACAAATATGGATA 1616
GraFAD78-1D       CTAAGGCGCCTATGTTGAAATTCATATGGAAACTTACAAATAAAATAATAAATATGGATA 1619
GhiFAD78-1D       CTAAGGCGCCTATGTTGAAATTCATATGGAAACTTACAAATAAAATAATAAATATGGATA 1611
                  ************************************************ ***********

GheFAD78-1A       GGAATGGAGTTACTTAAGGGGAGGTCTTACAACACTTGACCGTGACTATGGATGGATCAA 1679
GhiFAD78-1A       GGAATGGAGTTACTTAAGGGGAGGTCTTACAACACTTGACCGTGACTATGGATGGATCAA 1676
GraFAD78-1D       GGAATGGAGTTACTTAAGGGGAGGTCTTACAACACTTGACCGTGACTATGGATGGATCAA 1679
GhiFAD78-1D       GGAATGGAGTTACTTAAGGGGAGGTCTTACAACACTTGACCGTGACTATGGATGGATCAA 1671
                  ************************************************************

GheFAD78-1A       CAACATCCACCATGATATTGGAACCCATGTCATACACCATTTGTTTCCACAAATCCCACA 1739
GhiFAD78-1A       CAACATCCACCATGATATTGGAACCCATGTCATACACCATTTGTTTCCACAAATCCCACA 1736
GraFAD78-1D       CAACATCCACCATGATATTGGAACCCATGTCATACACCATTTGTTTCCACAAATCCCACA 1739
GhiFAD78-1D       CAACATCCACCATGATATTGGAACCCATGTCATACACCATTTGTTTCCACAAATCCCACA 1731
                  ************************************************************

GheFAD78-1A       CTACCATTTAGTAGAGGCGGTTAGTACCTTTACGTATCTCTTTTTTTTTT----CCACTT 1795
GhiFAD78-1A       CTACCATTTAGTAGAGGCGGTTAGTACCTTTACGTATCTCTTTTTTTTTTTTTCCCACTT 1796
GraFAD78-1D       TTACCATTTAGTAGAGGCGGTTAGTACCTTTACGTATCTCTTTTTTTTTT----CCACTT 1795
GhiFAD78-1D       TTACCATTTAGTAGAGGCGGTTAGTACCTTTACGTATCTCTTTTTTTTTT----CCACTT 1787
                   *************************************************    ******

GheFAD78-1A       TGATGTTCTCTTATTTCATTTTTTT-TCTAATTATTATGTTGGTATAATGTTGTGTCATA 1854
GhiFAD78-1A       TGATGTTCTCTTATTTCATTGGTTTATCTAATTATTATGTTGGTATAATGTTGTGTCATA 1856
GraFAD78-1D       TGATGTTGTCTTATTTCATTGGTTT-TCTAATTGTTATGTTGGTATAATGTTGTGTCATA 1854
GhiFAD78-1D       TGATGTTGTCTTATTTCATTGGTTT-TCTAATTGTTATGTTGGTATAATGTTGTGTCATA 1846
                  ******* ************  *** ******* **************************
                                           <-------S33R----------
GheFAD78-1A       GACCGAGGCAGCGAAGCCAGTTCTCGGAAAATACTATCGAGAGCCGGAACAATCAGGGCC 1914
GhiFAD78-1A       GACCGAGGCAGCGAAGCCAGTTCTCCGAAAATACTATCGAGAGCCGGAACAATCAGGGCC 1916
GraFAD78-1D       GACTGAGGCAGCGAGGCCAGTTCTCGGAAAATACTATCGAGAGCCGGAACAATCAGGGCC 1914
GhiFAD78-1D       GACTGAGGCAGCGAGGCCAGTTCTCGGAAAATACTATCGAGAGCCGGAACAATCAGGGCC 1906
                  *** ********** ********** **********************************
                                                                     <--------
GheFAD78-1A       TTTACCTTTCCACCTCATCGGAAGTTTGATGAGAAGCTTGAAGAACGATCATTATGTTAG 1974
GhiFAD78-1A       TTTACCTTTCCACCTCATCGGAAGTTTGATGAGAAGCTTGAAGAACGATCATTATGTTAG 1976
GraFAD78-1D       TTTACCTTTCCACCTCATCGGAAGTTTGATGAGAAGCTTGAAGAAAGATCACTATGTTAG 1974
GhiFAD78-1D       TTTACCTTTCCACCTCATCGGAAGTTTGATGAGAAGCTTGAAGAAAGATCACTATGTTAG 1966
                  ********************************************* ***** ********
                  ---S35R----------
GheFAD78-1A       TGACACTGGGGATGTTGTTTACTACCAAACTGACCCAGAACTCAAAAAGAATGCTTCATA 2034
GhiFAD78-1A       TGACACTGGGGATGTTGTTTACTACCAAACTGACCCAGAACTCAAAAAGAATGCTTCATA 2036
GraFAD78-1D       TGACACTGGGGATGTTGTTTACTACCAAACTGACCCAGAACTCAAAAAGAATGCTTCATA 2034
GhiFAD78-1D       TGACACTGGGGATGTTGTTTACTACCAAACTGACCCAGAACTCAAAAAGAATGCTTCATA 2026
                  ************************************************************

GheFAD78-1A       AAATAGCTCAGCCCCACAATAATGG      2059
GhiFAD78-1A       AAATAGCTCAGCCCCACACTAATGG C30R 2061
GraFAD78-1D       AAATAGCTCAGCCCCACAACAATGG C14R 2059
GhiFAD78-1D       AAATAGCTCAGCCCCGCATGCCAA  C16R 2050
                  *************** **
```
