# Supplementary material for: Genome-wide analysis of the omega-3 fatty acid desaturase gene family in Gossypium
Source: BMC Plant Biol. 2014 Nov 18;14:312. doi: 10.1186/s12870-014-0312-5 (PMC4245742; doi:10.1186/s12870-014-0312-5)
Supplement: Additional file 11: — Primer optimization for FAD3-type genes in G. hirsutum. The gene targets and primer pairs are listed on the left. Primer sequences are described in Additional file 3. PCR reactions were programmed with plasmid DNA containing either the target gene or the closest related homoeolog (listed beneath each gel picture). A gradient of annealing temperatures, with values listed above each gel, was used during the PCR reactions, then an equal volume of each reaction was analyzed by DNA gel electrophoresis and ethidium bromide staining. The optimal anneal temperature for each primer pair, where DNA fragments can be detected for the target gene, but not the homoeolog, is reported to the right. Also listed on the right are the expected sizes of PCR fragments amplified from either genomic DNA or mRNA. Note that the plasmid DNA templates contained genomic copies of each gene, and bands of expected sizes were obtained for all PCR reactions (DNA ladder not shown). [file 12870_2014_312_MOESM11_ESM.pdf]

```
GraFAD78-2D C17F                              GGCTGAACCTTTGGGAACCTCGTCCCAGAAC 34
GhiFAD78-2D C19F                                                         TTAG  9
GheFAD78-2A C17F GGCTGAACCTTTGGGAACCTTTTTTTAAAGGCTGAAACTTTGTACACCTCGTCCCAAAAA 60
GhiFAD78-2A C19F                                                         TTAG  9
                                                                           *

GraFAD78-2D      ACACTTAGCATCAGCCTCCAATGGCCAGTTGGGTTGTATCAGAATGTAGTTTAAGACCCT 91
GhiFAD78-2D      AGCTCTAGCATCAGCCTCCAATGGCCAGTTGGGTTGTATCAGAATGTAGTTTAAGACCCT 64
GheFAD78-2A      CCACTTAGCATCAGCCTCCAATGGCCAGTTGGGTTGTATCAGAATGTAGTTTAAGACCCT 120
GhiFAD78-2A      AGCTCTAGCATCAGCCTCCAATGGCCAGTTGGGTTGTATCAGAATGTAGTTTAAGACCCT 64
                      *******************************************************

GraFAD78-2D      TCACTGCCGTCAAAATCCACCATAAACCTAGACCTTCATTACCTTCAAACACCATTAATG 151
GhiFAD78-2D      TCACTGCCGTCAAAATCCACCATAAACCTAGACCTTCATTACCTTCAAACACCATTAATG 124
GheFAD78-2A      TCACTGCCGTTAAAATCCACCACAAACCTAGACCTTCATTACCTTCAAACACCATTAATG 180
GhiFAD78-2A      TCACTGCCGTTAAAATCCACCACAAACCTAGACCTTCATTACCTTCAAACACCATTAATG 124
                 ********** *********** *************************************

GraFAD78-2D      CTTTCAAGTTCAAACCTTTACCATGTTCTGCATTCAAGGTCCCAACTTTATGGTCTAAAG 211
GhiFAD78-2D      CTTTCAAGTTCAAACCTTTACCATGTTCTGCATTCAAGGTCCCAACTTTATGGTCTAAAG 184
GheFAD78-2A      CTTTCAAGTTCAAACCTTTACCGTGTTCTTCATTCAAGGTCCCAACTTTATGGTCTAAAG 240
GhiFAD78-2A      CTTTCAAGTTCAAACCTTTACCGTGTTCTTCATTCAAGGTCCCAACTTTATGGTCTAAAG 184
                 ********************** ****** ******************************

GraFAD78-2D      GTAGAAACTTGGGGGGATTGGGGCTGTTGAATGTGAGTGCTCCATTGAAAGTGACAATAA 271
GhiFAD78-2D      GTAGAAACTTGGGGGGATTGGGGCTGTTGAGTGTGAGTGCTCCATTGAAAGTGACAATAA 244
GheFAD78-2A      GTAGAAACTTAGGGGGGTTGGGGTTGTTGAATGTGAGTGCTCCATTGAAAGTGACAATAA 300
GhiFAD78-2A      GTAGAAACTTAGGGGGGTTGGGGTTGTTGAATGTGAGTGCTCCATTGAAAGTGACAATAA 244
                 ********** ***** ****** ****** ******************************
                           -----------S13F----------->
GraFAD78-2D      CTAGTGAAGAAGATAAAGGTAAAGAAGAGAGCACCACTGATGGGGTTAATAATGGTGAGT 331
GhiFAD78-2D      CTAGTGAAGAAGATAAAGGTAAAGAAGAGAGCACCACTGATGGGGTTAATAATGGTGAGT 304
GheFAD78-2A      CTAGTGAAGAAGATAAAGGTAAAGAAGAGAGCGCCATTGATGGGGTTAATAATGGTGAGT 360
GhiFAD78-2A      CTAGTGAAGAAGATAAAGGTAAAGAAGAGAGCGCCATTGATGGGGTTAATAATGGTGAGT 304
                 ******************************** *** ***********************

GraFAD78-2D      TTGACCCTGGTGCAGCACCTCCTTTTAAGTTGTCTGATATTAAAGCTGCAATACCAAAAC 391
GhiFAD78-2D      TTGACCCTGGTGCAGCACCTCCTTTTAAGTTGTCTGATATTAAAGCTGCAATACCAAAAC 364
GheFAD78-2A      TTGACCCTGGTGCAGCACCTCCTTTTAAGTTGTCTGATATTAAAGCTGCAATACCAAAAC 420
GhiFAD78-2A      TTGACCCTGGTGCAGCACCTCCTTTTAAGTTGTCTGATATTAAAGCTGCAATACCAAAAC 364
                 ************************************************************

GraFAD78-2D      ATTGTTGGGTTAAAGATCCATGGAGATCTATGAGTTATGTTGTGAGAGATGTTGTTGTTG 451
GhiFAD78-2D      ATTGTTGGGTTAAAGATCCATGGAGATCTATGAGTTATGTTGTGAGAGATGTTGTTGTTG 424
GheFAD78-2A      ATTGTTGGGTTAAAGATCCATGGAGATCTATGAGTTATGTTGTGAGAGATGTTGTTGTTG 480
GhiFAD78-2A      ATTGTTGGGTTAAAGATCCATGGAGATCTATGAGTTATGTTGTGAGAGATGTTGTTGTTG 424
                 ************************************************************

GraFAD78-2D      TGTTTGGATTGGCTGCTGTTGCTGCTTATTTCAATAACTGGTTTGTTTGGCCACTTTATT 511
GhiFAD78-2D      TGTTTGGATTGGCTGCTGTTGCTGCTTATTTCAATAACTGGTTTGTTTGGCCACTTTATT 484
GheFAD78-2A      TGTTTGGATTGGCTGCTGTTGCTGCTTATTTCAATAACTGGTTTGTTTGGCCACTTTATT 540
GhiFAD78-2A      TGTTTGGATTGGCTGCTGTTGCTGCTTATTTCAATAACTGGTTTGTTTGGCCACTTTATT 484
                 ************************************************************

GraFAD78-2D      GGATTGCTCAAGGAACCATGTTTTGGGCACTTTTTGTTCTTGGTCATGACTGGTAAATAA 571
GhiFAD78-2D      GGATTGCTCAAGGAACCATGTTTTGGGCACTTTTTGTTCTTGGTCATGACTGGTAAATAA 544
GheFAD78-2A      GGATTGCTCAAGGAACCATGTTTTGGGCACTTTTTGTTCTTGGTCATGACTGGTAAAAAA 600
GhiFAD78-2A      GGATTGCTCAAGGAACCATGTTTTGGGCACTTTTTGTTCTTGGTCATGACTGGTAAATAA 544
                 ********************************************************* **

GraFAD78-2D      TAATTATCCTGTTTTGCTTGTCTCCTTCTGTTTGTCACTTTTGGACCATAAAAACATTGA 631
GhiFAD78-2D      TAATTATCCTGTTTTGCTTGTCTCCTTCTGTTTGTCACTTTTGGACCATAAAAACATTGA 604
GheFAD78-2A      TAATTACCCTGTTTTGCTTGTCTTCTTCTGTTTGTCACTTCTGGACCATAAAAACATTGA 660
GhiFAD78-2A      TAATCACCCTGTTTTGCTTGTCTTCTTCTGTTTGTCACTTCTGGAGCATAAAAACATTGA 604
                 **** * **************** **************** **** **************

GraFAD78-2D      ATTTGGACATTGACATTGGTCTTGTTCTT----TTTTTTTTTTTTTTTTGCAGTGGACAT 687
GhiFAD78-2D      ATTTGGACATTGACATTGGTCTTGTTCTTCTTCTTTTTTTTTTTTTTTTGCAGTGGACAT 664
GheFAD78-2A      ATTTGGACATTGACATTGGTCTTGTTCTT------CTTTTTTTTTTT--GCAGTGGACAT 712
GhiFAD78-2A      ATTTGGACATTGACATTGGTCTTGTTCTT------CTTTTTTTTTTTTTGCAGTGGACAT 658
                 *****************************       ***********  **********
```

```
GraFAD78-2D     GGTAGTTTTTCCAATAATCCAGCTTTGAATAGTGTTGCAGGTCATCTTCTTCATTCTTCA 747
GhiFAD78-2D     GGTAGTTTTTCCAATAATCCAGCTTTGAATAGTGTTGCAGGTCATCTTCTTCATTCTTCA 724
GheFAD78-2A     GGTAGTTTTTCCAATAATCCAGCTTTGAATAGTGTTGCAGGTCATCTTCTTCATTCTTCA 772
GhiFAD78-2A     GGTAGTTTTTCCAATAATCCAGCTTTGAATAGTGTTGCAGGTCATCTTCTTCATTCTTCA 718
                ************************************************************

GraFAD78-2D     ATCCTTGTTCCATACCATGGATGGTCAGTTCTCTGAACTTTTTCTTTTGGTCAAATTCTG 807
GhiFAD78-2D     ATCCTTGTTCCATACCATGGATGGTCAGTTCTCTGAACTTTTTCTTTTGGTCAAATTCTG 784
GheFAD78-2A     ATCCTTGTTCCATACCATGGATGGTTAGTTCTCTGAACTTTTTCTTTTGGTTAAATTCTG 832
GhiFAD78-2A     ATCCTTGTTCCATACCATGGATGGTTAGTTCTCTGAACTTTTTCTTTTGGTTAAATTCT- 777
                ************************* ************************* *******

GraFAD78-2D     CTATTAGTCCCTCTACTTTACAGAAATTGTTAATTTGGTCATTTTTAATCCTTGTAATTT 867
GhiFAD78-2D     CTATTAGTCCCTCTACTTTACAGAAATTGTTAATTTGGTCATTTTTAATCCTTGTAATTT 844
GheFAD78-2A     CTATTAGTCCCTCTACTTTACAGAAATTGTTAATTTGGTCATTTTTAATCCTTGTAATTT 892
GhiFAD78-2A     CTATTAGTCCCTCTACTTTACAGAAATTATTAATTTGGTCATTTTTAATCCTTGTAATTT 837
                **************************** *******************************

GraFAD78-2D     CAGTCCGCACTGCTATTTTCAAAATTCAATGCAGTTATACCTATAATGCTTTTTCAGCAT 927
GhiFAD78-2D     CAGTCCGCACTGCTATTTTCAAAATTCAATGTAGTTATACGTGTAATGCTTTTTCAGCAT 904
GheFAD78-2A     CAGTCTGCACTGCTATTTTCAAAATTCAATGCAGTTATACGTGTAATGCTTTTTCAGCAT 952
GhiFAD78-2A     CAGTCTGCACTGCTATTTTCAAAATTCAATGCAGTTATACGTGTAATGCTTTTTCAGCAT 897
                ***** ************************* ******** * *****************

GraFAD78-2D     GTTATTTCAACATATTACTTACTAAAAATCTGGTTAATAGATTAACGACTATTATTTACT 987
GhiFAD78-2D     GTTATTTCAACATATTACTTACTAAAAATCTGGTTAATAGATTAACGACTATTATTTACT 964
GheFAD78-2A     GTTATTTCAACATATTATTTACTAAAAATCCAGTTAATAGATTAACAACTATCATCTACT 1012
GhiFAD78-2A     GTTATTTCAACATATTATTTACTAAAAATCCGGTTAATAGATTAACGACTATCATCTACT 957
                ***************** ************  ************** ***** ** ****

GraFAD78-2D     TCAAGCTTGAAATTTCAAAATTCAAAATCTGTA-GGACTTAGAATGATCCAATTAGAGAA 1046
GhiFAD78-2D     TCAAGCTTGAAATTTCAAAATTCAAAATCTGTA-GGACTTAGAATAATCCAATTAGAGAA 1023
GheFAD78-2A     TCAAGCTTGAAATTTCAAAATTCAAAATCTGTAAGGACTTAGAATGATCTAATTAGAGAA 1072
GhiFAD78-2A     TCAAGCTTGAAATTTCAAAATTCAAAATCTGTAAAGACTTAGAATGATCCAATTAGAGAA 1017
                *********************************  ********** *** **********

GraFAD78-2D     TACAGACTAAATCTACAACTGTACACAGTACAAGACTAGTAATTGAATTTAACCAATAAG 1106
GhiFAD78-2D     TACGGACTAAATCTACAACTGTACACAGTACAAGACTAGTAATTGAATTTAACCAATAAG 1083
GheFAD78-2A     TATGGACTAAATCTACAACTGTACACAATACAAGACTAGTAATTGAATTTAACCAATAAG 1132
GhiFAD78-2A     TATGAACTAAATCTACAACTGTACACAGTACAAGACTATTAATTGAATTTAACCAATAAG 1077
                **   ********************** ********** *********************

GraFAD78-2D     GATTAAAATTGGTCAAATTAAAGTATAAGGACTAAATTCACAACTTTTACAAAGTACAGG 1166
GhiFAD78-2D     GATTAAAATTGGTCAAATTAAAGTATAAGGACTAAATCCACAACTTTTACAAAGTACAGG 1143
GheFAD78-2A     GATTAAAATTGGTCAAACTAAAGTATAAGGACTAAATCCACAACTTTTACAAAGTACAGG 1192
GhiFAD78-2A     GATTAAAATTAGTCAAATTAAAGTATAAGGACTAAATCCACAACTTTTACAAAGTACAAG 1137
                ********** ****** ******************* ******************** *

GraFAD78-2D     GACTAATTGCAGAATTTAACATTTACTTTTATATCCAATTACCAACTTCTTTCAACAATT 1226
GhiFAD78-2D     GACTAATTGCAGAATTTAACATTTACTTTTATATCCAATTACCAACTTCTTTCAACAATT 1203
GheFAD78-2A     GACTTATAGCAGAATTTAACATTTACTTTTATATCCAATTACCAACTTCTTTCAACAATT 1252
GhiFAD78-2A     GAATTATAGCAGAATTTAACATTTACTTTTATATCCAATTACCAACTTCTTTCAACAATT 1197
                ** * ** ****************************************************

GraFAD78-2D     TTGATCGTATATGCAGGAGAATTAGCCATAGAACTCATCACCAGAATCATGGGCACATCG 1286
GhiFAD78-2D     TTGATCGTATATGCAGGAGAATTAGCCATAGAACTCATCACCAGAATCACGGGCACGTCG 1263
GheFAD78-2A     TTGATCATATATGCAGGAGAATTAGCCATAGAACTCATCACCAGAATCACGGGCATGTCG 1312
GhiFAD78-2A     TTGATCATATATGCAGGAGAATTAGCCATAGAACTCATCACCAGAATCACGGGCATGTCG 1257
                ****** ****************************************** *****  ***

GraFAD78-2D     AAAATGACGAGTCATGGCATCCGGTAATGTCGGCCACCACAACCCTTTCTTACATGTAAA 1346
GhiFAD78-2D     AAAATGACGAGTCATGGCATCCGGTAATGTTGGCCACCACAACCCTTTCTTACATGTAAA 1323
GheFAD78-2A     AAAATGACGAGTCATGGCATCCGGTAATGTCCGCCGCCACAACCCTTTCTTACATGTAAA 1372
GhiFAD78-2A     AAAATGACGAGTCATGGCATCCGGTAACGTCCGCCGCCACAACCCTTTCTTACATGTAAA 1317
                *************************** **  *** ************************

GraFAD78-2D     TTTTCATTACAAGTGAGTAATTTCTTTACCTTTTATTGCAGTTGTCTGAGAAAATTTACA 1406
GhiFAD78-2D     TTTTCATTACAAGTGAGTAATTTCTTTACCTTTTATTGCAGTTGTCTGAGAAAATTTACA 1383
GheFAD78-2A     TTTTCATTACAAGTGAGTAATTTCTTTACCTTTTATGGCAGTTGTCTGAGAAAATTTACA 1432
GhiFAD78-2A     TTTTCATTACAAGTGAGTAATTTCTTTACCTTTTATGGCAGTTGTCTGAGAAAATTTACA 1377
                ************************************ ***********************
```

```
GraFAD78-2D     AGAGTTTGGACAATGCAACAAGATTATTGAGGTTCACCTTGCCTTTCCCTATGCTTGCTT 1466
GhiFAD78-2D     AGAGTTTGGACAATGCAACAAGATTATTGAGGTTCACCTTGCCTTTCCCTATGCTTGCTT 1443
GheFAD78-2A     AGAGTTTGGACAATGCAACAAGATTATTGAGGTTCACCCTGCCTTTCCCTATGCTTGCTT 1492
GhiFAD78-2A     AGAGTTTGGACAATGCAACAAGATTATTGAGGTTCACCCTGCCTTTCCCTATGCTTGCTT 1437
                ************************************** *********************

GraFAD78-2D     ATCCTATCTATCTGGTAAGCCATAGATGTTGGATTCGGTTTACGAATCGAGTGATTCGTG 1526
GhiFAD78-2D     ATCCTATCTATCTGGTAAGCCATAGATGTTGGATTCGGTTTACGAATCGAGTGATTCGTG 1503
GheFAD78-2A     ATCCTATCTATCTGGTAAGCCATAGATGTTGGATTCGGTTTACGAATCAAATGAT----A 1548
GhiFAD78-2A     ATCCTATCTATCTGGTAAGCCATAGATGTTGGATTCGGTTTACGAATCGAATGAT----A 1493
                ************************************************ * ****

GraFAD78-2D     TTTATATTGACTCATGTTGTTTCCGATCAACAGTGGAGTCGAAGTCCCGGAAAAAAAGGGT 1586
GhiFAD78-2D     TTTATATTGACTCATGTTGTTTCCGATAAACAGTGGAGTCGAAGTCCCGGAAAAAAAGGGT 1563
GheFAD78-2A     TTTATATTGACTCATGTTGTTTACGGTCAACAGTGGAGTCGAAGTCCCGGAAAAAAAGGGT 1608
GhiFAD78-2A     TTTATATTGACTCATGTTGTTTACGGTCAACAGTGGAGTCGAAGTCCCGGAAAAAAAGGGT 1553
                ********************** ** * ********************************

GraFAD78-2D     TCACATTTCCATCCGGACAGTGATTTATTTGTCCCAAATGAAAGGAAAGATATAATCACC 1646
GhiFAD78-2D     TCACATTTCCATCCAGACAGTGATTTATTTGTCCCAAATGAAAGGAAAGATATAATCACC 1623
GheFAD78-2A     TCACATTTCCATCCAGACAGTGATTTATTTGTCCCAAATGAAAGGAAAGATATAATCACC 1668
GhiFAD78-2A     TCACATTTCCATCCAGACAGTGATTTATTTGTCCCAAATGAAAGGAAAGATATAATCACC 1613
                ************** *********************************************
                                            --------S15F-------->
GraFAD78-2D     TCAACTGCTTGTTGGACGGCAATGGTTGGCTTGCTTGCATATTTGTCTTTTGCAATGGGA 1706
GhiFAD78-2D     TCAACTGCTTGTTGGACGGCAATGGTTGGCTTGCTTGCATATTTGTCTTTTGCAATGGGA 1683
GheFAD78-2A     TCAACTGCTTGTTGGACAGCAATGGTTGGCTTGCTTGCATATTTGTCGTTTACAATGGGA 1728
GhiFAD78-2A     TCAACTGCTTGTTGGACAGCAATGGTTGGCTTGCTTGCATATTTGTCGTTTACAATGGGA 1673
                ***************** ***************************** *** ********

GraFAD78-2D     CCAATGCCATTGCTTAAACTCTACGGCATCCCTTATGCGGTACATGCTTCAGTTCCTACA 1766
GhiFAD78-2D     CCAATGCCATTGCTTAAACTCTACGGCATCCCTTATGCGGTACATGCTTCAGTTCCTACA 1743
GheFAD78-2A     CCAATGCCATTGCTTAAACTCTACGGCATCCCTTATGCGGTACATGCTTCAGTTCCTACA 1788
GhiFAD78-2A     CCAATGCCATTGCTTAAACTCTACGGCATCCCTTATGCAGTACATGCTTCAGTTCCTACA 1733
                ************************************** *********************

GraFAD78-2D     TATATTTCTTCAAAACAATTTCGGTAAAAGTACTACGAAGGCCTTTAGATTAGAAATCGA 1826
GhiFAD78-2D     TATATTTCTTCAAAACAATTTCGGTAAAAGTACTACGAAGGCCTTTAGATTAGAAATCGA 1803
GheFAD78-2A     TATATTTCTTCAAAACAATTTCGGTAAAAGTACTACGAAGGCCTTTAGACTAGAAACCAA 1848
GhiFAD78-2A     TATATTTCTTCAAAACAATTTCGGTAAAAGTACTACGAAGGCCTTTAGACTAGAAACCGA 1793
                ************************************************* ****** * *

GraFAD78-2D     ATTGCATTTTACCCCTTCTATTTAAAATAAGCAAAATGGTCCTTCTGTTAAAATTTTTAT 1886
GhiFAD78-2D     ATTGCATTTTACCCCTTCTATTTAAAATAAGCAAACTGGTCCTTCTGTTAAAATTTTTAT 1863
GheFAD78-2A     ATTGCATTTTACCCCCTCTATTTAAAATAAGCAAACTGGTCCTTCT-------------- 1894
GhiFAD78-2A     ATTGCATTTTACCCCCTCTATTTAAAATAAGCAAACTGGTCCTTCT-------------- 1839
                *************** ******************* **********

GraFAD78-2D     CCATTTTTAGTGTTAAAAGTTGACCCTTATGTTCGGTTATTCAGTCAGTCCAGCAGTTTT 1946
GhiFAD78-2D     CCATTTT-AGTGTTAAAAGTTGACCCTTATGTTTGGTTATTCAGCCAGTCCAGCAGTTTT 1922
GheFAD78-2A     ----------------------------ACGTT---------------------AGTTTT 1905
GhiFAD78-2A     ----------------------------ACGTT---------------------AGTTTT 1850
                                            * ***                     ******

GraFAD78-2D     TAACAATAAAAATGAATGAAATTTTTACCAGAAAGGCCAAGTTTGCTCTTTAATCTAACG 2006
GhiFAD78-2D     TAACAATAAAAATGAATGAAATTTTTACCAGAAAGGCCAAGTTTGCTCTTTAATCTAACA 1982
GheFAD78-2A     TAACAATAAAAATGAACGAAATTTTTAACAGAAAGGCCAAATTTGCTCTTTAATCTAACA 1965
GhiFAD78-2A     TAACAATAAAAATGAACGAAATTTTTAACAGAAAGGCCAAGTTTGCTCTTTAATCTAACA 1910
                **************** ********** ************ ******************

GraFAD78-2D     TAGAAGGACTAAATTGCCCATTTTTTGAGTGAATGGAGTAAAATGCAATCTAACTCTCAG 2066
GhiFAD78-2D     TACAAGGACCAATTTGCCCATTTTTTGAGTGAATGGAGTAAAATGCAATCTAACTCTCAG 2042
GheFAD78-2A     TACAGGGACCAATTTGCCCATTTTTTGAGTGAATGGAGTAAAATGCAATCTAACTCTCAG 2025
GhiFAD78-2A     TACAGGGACCAATTTGCCCATTTTTTGAGTGAATGGAGTAAAATGCAATCTAACTCTCAG 1970
                ** * **** ** ***********************************************

GraFAD78-2D     TACAGGG-CCTTAATGGTACTTCA--AAAAAAAAATCATATTTCTGGTCTGATATAATGG 2123
GhiFAD78-2D     TAGAGGGGCCTTAATGGTACTTTTACAAAAAAAAATCATATTTCTGGTCTGATATAATGG 2102
GheFAD78-2A     TACAGGGGTCTTAATGGTACTTTTACCAAAAAAATTCATATTTCTGGTCTGATATAATGG 2085
GhiFAD78-2A     TACAGTGGTCTTAATGGTACTTTTACCAAAAAAATTCATATTTCTGGTCTGATATAATGG 2030
                ** ** *  *************     ******* *************************
```

```
GraFAD78-2D     ATTCACATATAAAATTGCAGATATTTGTGATGTGGTTGGATTTGGTGACATACCTACATC 2183
GhiFAD78-2D     ATTCACATATAAAATTGCAGATATTTGTGATGTGGTTGGATTTGGTGACATACCTACATC 2162
GheFAD78-2A     ACTCACATATAAAATGGCAGATATTTGTGATGTGGTTGGATTTGGTGACATACCTACATC 2145
GhiFAD78-2A     ACTCACATATAAAATGGCAGATATTTGTGATGTGGTTGGATTTGGTGACATACCTACATC 2090
                * ************* ********************************************

GraFAD78-2D     ACCATGGTCATGACGAGAAGCTTCCATGGTATCGAGGAAAGGTTCGGGATAATGTTTATG 2243
GhiFAD78-2D     ACCATGGTCATGACGAGAAGCTTCCATGGTATCGAGGAAAGGTTCGGGATAATGTTTATG 2222
GheFAD78-2A     ACCATGGTCATGACGAGAAGCTTCCATGGTATCGAGGAAAGGTTCAGGATAATGTTTATG 2205
GhiFAD78-2A     ACCATGGTCATGACGAGAAGCTTCCATGGTATCGAGGAAAGGTTCGGGATAATGTTTATT 2150
                ********************************************* ************* 

GraFAD78-2D     CTTCTTCACCAAGAAACCATCCATGTTATGTATGGTATAACAATTGAAACTTCTCTGTGT 2303
GhiFAD78-2D     CTTCTTCACCAAGAAACCATCCATGTTATGTATGGTATAACAATTGAAACTTCTCTGTGT 2282
GheFAD78-2A     CTTCTTCACCAAGAAACCATCCATGTTATGTATGGTATAACAATTGAAACTTCTCTGTGT 2265
GhiFAD78-2A     CTTCTTCACCAAGAAACCATCCATGTTATGTATGGTATAACAATTGAAACTTCTCTGTGT 2210
                ************************************************************
                                                      <---------S37R------
GraFAD78-2D     TTTACTAACCAGGAGTGGAGTTACCTGAGAGGAGGGCTGACGACGCTTGATCGGGATTAT 2363
GhiFAD78-2D     TTTACTAACCAGGAGTGGAGTTACCTGAGAGGAGGGCTGACGACGCTTGATCGGGATTAT 2342
GheFAD78-2A     TTTACTAACCAGGAGTGGAGTTACCTGAGAGGAGGGCTTACGACGCTTGATCGGGATTAT 2325
GhiFAD78-2A     TTTACTAACCAGGAGTGGAGTTACCTGAGAGGAGGGCTTACGACGCTTGATCGGGATTAT 2270
                ************************************** *********************

GraFAD78-2D     GGATGGATTAACAACATCCACCACGATATCGGAACTCATGTTATACATCATCTCTTCCCT 2423
GhiFAD78-2D     GGATGGATTAACAACATCCACCACGATATCGGAACTCATGTTATACATCATCTCTTCCCT 2402
GheFAD78-2A     GGATGGATTAACAACATCCACCACGATATTGGAACTCATGTTATACATCATCTCTTCCCT 2385
GhiFAD78-2A     GGATGGATTAACAACATCCACCACGATATTGGAACTCATGTTATACATCATCTCTTCCCT 2330
                **************************** *******************************

GraFAD78-2D     CAAATCCCACATTATCACTTGATTGAAGCTGTGAGTATCATCATCATCATC--------- 2474
GhiFAD78-2D     CAAATCCCACATTATCACTTGATTGAAGCTGTGAGTATCATCATCATCATC--------- 2453
GheFAD78-2A     CAAATCCCACATTATCACTTGATTGAAGCTGTGAGTATCATCATCATCATCATCATCATC 2445
GhiFAD78-2A     CAAATCCCACATTATCACTTGATTGAAGCTGTGAGTATCATCATCATCATCATC------ 2384
                ***************************************************

GraFAD78-2D     ---TTATTTTCCTGCATCAGTTATGTTTTAGGTTTTCCACATACTAAAGAAAATGTTACA 2531
GhiFAD78-2D     ---TTATTTTCCTGCATCAGTTATGTTTTAGGTTTTCCACATACTAAAGAAAATGTTATA 2510
GheFAD78-2A     ATCTTATTTTCCTGCATCAGTTATGTTTTAGGTTTTCCACATACTAAAGAAAATGTTACA 2505
GhiFAD78-2A     ATCTTATTTTCCTGCATCAGTTATGTTTTAGGTTTTCCACATACTAAAGAAAATGGTACA 2444
                   **************************************************** ** *

GraFAD78-2D     TGAATTACAGACCGAAGCAGCAAAGCCAGTGCTCGGGAAATACTACAGGGAGCCAAAGAA 2591
GhiFAD78-2D     TGAATTACAGACCGAAGCAGCAAAGCCAGTGCTCGGGAAATACTACAGGGAGCCAAAGAA 2570
GheFAD78-2A     TGAATTACAGACCGAAGCAGCGAAGCCAGTGCTCGGGAAATACTACAGGGAGCCAAAGAA 2565
GhiFAD78-2A     TGAATTACAGACCGAAGCAGCGAAGCCAGTGCTCGGGAAATACTACAGGGAGCCAAAGAA 2504
                ******************** ***************************************
                   <-------S16R-----------
GraFAD78-2D     ATCTGGGCCTTTACCATTTTATTTACTCGGGATACTCATAAAAAGCATGAGAAAGGATCA 2651
GhiFAD78-2D     ATCTGGGCTTTTACCATTTTATTTACTCGGGATACTCATAAAAAGCATGAGAAAGGATCA 2630
GheFAD78-2A     ATCCGGGCCTTTACCATTTTATTTACTTGGGATACTCATAAAAAGCATGAGAAAGGATCA 2625
GhiFAD78-2A     ATCCGGGCCTTTACCATTTTATTTACTTGGGATACTCATAAAAAGCATGAGAAAGGATCA 2564
                *** **** ****************** ********************************

GraFAD78-2D     TTATGTGAGTGACATTGGTGATGTCGTGTACTATCAAACAGATCCTCAACTATATGGAAC 2711
GhiFAD78-2D     TTATGTGAGTGACATTGGTGATGTCGTGTACTATCAAACAGATCCTCAACTATATGGAAC 2690
GheFAD78-2A     TTATGTGAGTGACATTGGTGATGTTGTGTACTATCAAACAGATCCTCAACTATATGGAAC 2685
GhiFAD78-2A     TTATGTGAGTGACATTGGTGATGTTGTGTACTATCAAACAGATCCTCAACTATATGGAAC 2624
                ************************ ***********************************

GraFAD78-2D     TAACAAATCAGATTGAAGCACTGAAGTTTCTTTTTATTCTCTGGCTAAAACCTCTATTCGG C18R 2772
GhiFAD78-2D     TAACAAATCAGATTGAAGCCCTGAAGTTTCTGCATGCTAA                      C20R 2730
GheFAD78-2A     TAACAAATCAGATTGAAGCACTGAAGTTTCTTTTTATTCTCTGGCTAAAACCTCTATTCGG C18R 2746
GhiFAD78-2A     TAACAAATCAGATTGAAGCCCTGAAGTTTCTGCATGCTAA                      C20R 2664
                ******************* ***********   *  *
```
