# Supplementary material for: Genome-wide analysis of the omega-3 fatty acid desaturase gene family in Gossypium
Source: BMC Plant Biol. 2014 Nov 18;14:312. doi: 10.1186/s12870-014-0312-5 (PMC4245742; doi:10.1186/s12870-014-0312-5)
Supplement: Additional file 12: — Primer optimization for FAD7/8-type genes in G. hirsutum. (A) The gene targets and primer pairs are listed on the left. Primer sequences are described in Additional file 3. PCR reactions were programmed with plasmid DNA containing either the target gene or the closest related homoeolog, as indicated above each column of gel pictures. A gradient of annealing temperatures, with values listed immediately above the gel pictures, was used during the PCR reactions, then an equal volume of each reaction was analyzed by DNA gel electrophoresis and ethidium bromide staining. The optimal anneal temperature for each primer pair, where DNA fragments could be detected for the target gene, but not the homoeolog, is reported to the right. Also listed are the expected sizes of PCR fragments amplified from either genomic DNA or mRNA. Note that the plasmid DNA templates contained genomic copies of each gene, and bands of expected sizes were obtained for all PCR reactions (DNA ladder not shown). (B) RT-PCR analysis of RNA extracted from 12-day-old G. hirsutum cotyledons, showing that FAD7/8-1 and FAD7/8-2 genes, from both subgenomes, are predominantly expressed. Bands of expected sizes, as amplified from cDNA and not genomic DNA, were detected, and no bands were observed in the actin control when PCR reactions were programmed with RNA that was not subjected to reverse transcription (no RT). [file 12870_2014_312_MOESM12_ESM.pdf]

```
GraFAD78-3D C21F TGAGGTTCAATTTCCAAGTCGCA--ATATTTAAAAATAAGAAAGAAAAAAGAGAAGAAAC 58
GhiFAD78-3D      ------------------------------------------------------------
GheFAD78-3A C21F TGAGGTTCAATTTCCAAGTCGCACAATATTTAAAAATAAGAAAGAAAAAAGAGAAGAAAC 60
GhiFAD78-3A      ------------------------------------------------------------

GraFAD78-3D      GTCTCAACTCTTTAACAGGCTCTCATTTAGCCGTTTCTGCTATGAAACCCCCAGTAACCC 118
GhiFAD78-3D      ------------------------------------------------------------
GheFAD78-3A      GTCTCAACTCTTTAACAGGCTCTCATTCAGCCGTTTCTGCTATGAAACCCCCAGTAACCC 120
GhiFAD78-3A      ------------------------------------------------------------

GraFAD78-3D      TAAATATAATGCTCCTCAGTCCTTTACTTCCTAACCAAGTAACCTGCCAGGAACCTCTCA 178
GhiFAD78-3D      ----------------------------------------------------------
GheFAD78-3A      TAAATATAATGCTCCTCAGTCCTTTGCTTCCTAACCAAGTAACCTGCCAGGAACCTCTCA 180
GhiFAD78-3A      ----------------------------------------------------------

GraFAD78-3D      GTTTCTTTTTTTTTTTAAGAACCTTTGAGCTTTTACCTGGGATTTTAATGGAGAGTTTTC 238
GhiFAD78-3D C24F ------------------------GGTGAGCTCTACCTGGGATTTTAATGGAGAGTTTTC 36
GheFAD78-3A      GTTTCTTTTTTTT---AAGAACCTTTGAGCTTTTACCTGGGATTTCAATGGAGAGTTTTC 237
GhiFAD78-3A C24F ------------------------GGTGAGCTCTACCTGGGATTTTAATGGAGAGTTTTC 36
                                                   *********** **************

GraFAD78-3D      TCATATCAAAATCTGGTTTAAAGCCTCTTCCTCGTATCTACATAAGACCCATAACTGGTC 298
GhiFAD78-3D      TCATATCAAAATCTGGTTTAAAGCCTCTTCCTCGTATCTACATAAGACCCATAACTGGTC 96
GheFAD78-3A      TCATATCAAAATCTGGTTTAAAGCCTCTTCCTCGTATCTGCATAAGACCCATAACTGGTC 297
GhiFAD78-3A      TCATATCAAAATCTGGTTTAAAGCCTCTTCCTCGTATCTGCATAAGACCCATAACTGGTC 96
                 *************************************** ********************

GraFAD78-3D      TTGTCCCAAGAAATTACTTCTCAAAGCCAAGATTTTTACATACAAGCAAGTGTTTTTCTG 358
GhiFAD78-3D      TTGTCCCAAGAAATTACTTCTCAAAGCCAAGATTTTTACATACAAGCAAGTGTTTTTCTG 156
GheFAD78-3A      TTGTCCCAAGAAATTACTTCTCAAAGCCAAGATTTTTACATACAAGCAAGTGTTTTTCTG 357
GhiFAD78-3A      TTGTCCCAAGAAATTACTTCTCAAAGCCAAGATTTTTACATACAAGCAAGTGTTTTTCTG 156
                 ************************************************************

GraFAD78-3D      ATCTAAAGGTGACTACACCATTGAAAGTTGCATCCGTAGAAGAAGATGAAGAAATAAATG 418
GhiFAD78-3D      ATCTAAAGGTGACTACACCATTGAAAGTTGCATCCGTAGAAGAAGATGAAGAAATAAATG 216
GheFAD78-3A      ATCTAAAGGTGACTACGCCATTGAAAGTTGCATCCGTAGAAGAAGATGAAGAAATAAATG 417
GhiFAD78-3A      ATCTAAAGGTGACTACGCCATTGAAAGTTGCATCCGTAGAAGAAGATGAAGAAATAAATG 216
                 **************** *******************************************
                                         -----------S21F--------->
GraFAD78-3D      AGAGAATGCATGGTATTAACAAGATTGGAGAGCAAGAAGAAGAGACAATATTTGACCCTG 478
GhiFAD78-3D      AGAGAATGCATGGTATTAACAAGATTGGAGAGCAAGAAGAAGAGACAATATTTGACCCTG 276
GheFAD78-3A      AGAGAATGCATGGTATTAACAAGCTTGGAGAGCAAGAACAAGAGACAAGATTTGACCCTG 477
GhiFAD78-3A      AGAGAATGCATGGTATTAACAAGCTTGGAGAGCAAGAACAAGAGACAAGATTTGACCCTG 276
                 *********************** ************** ********* ***********

GraFAD78-3D      CAGCTCCCCCACCGTTTAACTTAGCTGATGTAAGAGCAACCATACCCCAGCATTGTTGGG 538
GhiFAD78-3D      CAGCTCCCCCACCGTTTAACTTAGCTGATGTAAGAGCAACCATACCCCAGCATTGTTGGG 336
GheFAD78-3A      CAGCTCCCCCACCGTTTAACTTAGCTGATGTAAGAGCAAGCATACCCCAGCATTGTTGGG 537
GhiFAD78-3A      CAGCTCCCCCACCGTTTAACTTAGCTGATGTAAGAGCAAGCATACCCCAGCATTGTTGGG 336
                 *************************************** ********************

GraFAD78-3D      TAAAGGATCCATGGAAATCTATGAGCTATGTTGTGAGGGATGTTGCTCTTGTTTTAAGCT 598
GhiFAD78-3D      TAAAGGATCCATGGAAATCTATGAGCTATGTTGTGAGGGATGTTGCTCTTGTTTTAAGCT 396
GheFAD78-3A      TAAAGGATCCATGGAAATCTATGAGCTATGTTGTGAGGGATGTTGCTCTTGTTTTAAGCT 597
GhiFAD78-3A      TAAAGGATCCATGGAAATCTATGAGCTATGTTGTGAGGGATGTTGCTCTTGTTTTAAGCT 396
                 ************************************************************

GraFAD78-3D      TGGCTGCTGCTGTGGTTTATGTTAACAACTGGCTTGTTTGGCCTCTTTACTGGGTTGCTC 658
GhiFAD78-3D      TGGCTGCTGCTGTGGTTTATGTTAACAACTGGCTTGTTTGGCCTCTTTACTGGGTTGCTC 456
GheFAD78-3A      TGGCTGCTGCTGTGGTTTATGTTAACAACTGGCTTGTTTGGCCTCTTTACTGGGTTGCTC 657
GhiFAD78-3A      TGGCTGCTGCTGTGGTTTATGTTAACAACTGGCTTGTTTGGCCTCTTTACTGGGTTGCTC 456
                 ************************************************************

GraFAD78-3D      AAGGAACCATGTTTTGGGCTCTTTTTGTTCTTGGTCATGACTGGTAATTCATACAATGCA 718
GhiFAD78-3D      AAGGAACCATGTTTTGGGCTCTTTTTGTTCTTGGTCATGACTGGTAATTCATACAATGCA 516
GheFAD78-3A      AAGGAACCATGTTTTGGGCTCTTTTTGTTCTTGGTCATGACTGGTAATTCATACAATGCA 717
GhiFAD78-3A      AAGGAACCATGTTTTGGGCTCTTTTTGTTCTTGGTCATGACTGGTAATTCATACAATGCA 516
                 ************************************************************
```

```
GraFAD78-3D     TTGGACACAACCTTCTTTAATTAGGCGTGTGACTGTCTCAATTTATATGTTTATTTTCTT 778
GhiFAD78-3D     TTGGACACAACCTTCTTTAATTAGGCGTGTGACTGTCTCAATTTATATGTTTATTTTCTT 576
GheFAD78-3A     TTGGACACAACCTTCTTTAATTAGGCGTGTGACTGTCTCAGTTTATATGTTTATTTTCTT 777
GhiFAD78-3A     TTGGACACAACCTTCTTTAATTAGGCGTGTGACTGTCTCAGTTTATATGATTATTTTCTT 576
                **************************************** ******** **********

GraFAD78-3D     TTCAAGGCTTAATTGGAATACATTGTGCAGCGGCCATGGCAGCTTTTCAAACAACCCCAA 838
GhiFAD78-3D     TTCAAGGCTTAATTGGAATACATTGTGCAGCGGCCATGGCAGCTTTTCAAACAACCCCAA 636
GheFAD78-3A     TTCAAGGCTTAATTGGAATTCATTGTGCAGCGGCCATGGCAGCTTTTCAAACAACCCCAA 837
GhiFAD78-3A     TTCAAGGCTTAATTGGAATTCATTGTGCAGCGGCCATGGCAGCTTTTCAAACAACCCCAA 636
                ******************* ****************************************

GraFAD78-3D     GTTAAACAGTTTAGTGGGGCATCTATTGCATTCTTTCATTCTTGTGCCTTACCATGGATG 898
GhiFAD78-3D     GTTAAACAGTTTAGTGGGGCATCTATTGCATTCTTTCATTCTTGTGCCTTACCATGGATG 696
GheFAD78-3A     GTTAAACAGTTTAGTGGGGCATCTATTGCATTCTTCCATTCTTGTGCCTTATCATGGATG 897
GhiFAD78-3A     GTTAAACAGTTTAGTGGGGCATCTATTGCATTCTTCCATTCTTGTGCCTTATCATGGATG 696
                *********************************** *************** ********

GraFAD78-3D     GTAAAATTTTGTCATAATGTTAAACTTAGTCCTCGATGTTTGCATTTTTTTGTTATTTTG 958
GhiFAD78-3D     GTAAAATTTTGTCATAATGTTAAACTTAGTCCTCGATGTTTGCATTTTTTTGTTATTTTG 756
GheFAD78-3A     GTAAAATTTTGTCATAATGTTGAACTTAGTCCTCGATGTTTGCATTTTTTTGTTATTTTG 957
GhiFAD78-3A     GTAAAATTTTGTCATAATGTTGAACTTAGTCCTCGATGTTTGCATTTTTTTGTTATTTTG 756
                ********************* **************************************

GraFAD78-3D     TCCATTATTCTTTATCACATGAGTTTCCACACCATAGTTGAATTCACTTTCATTATGATT 1018
GhiFAD78-3D     TCCATTATTCTTTATCACATGAGTTTCCACGCCATAGTTGAATTCACTTTCATTATGATT 816
GheFAD78-3A     TCCATTATTCTTTATTACATGAGTTTCCACACCACAGTTGAATTCACTTTCATTATGATT 1017
GhiFAD78-3A     TCCATTATTCTTTATTACATGAGTTTCCACACCACAGTTGAATTCACTTTCATTATGATT 816
                *************** ************** *** *************************

GraFAD78-3D     AACTTTTACAGGAGAATTAGCCATAGGATTCATCACCAAAACCAGGGTCATGTTGAGAAT 1078
GhiFAD78-3D     AACTTTTACAGGAGAATTAGCCATAGGATTCATCACCAAAACCAGGGTCATGTTGAGAAT 876
GheFAD78-3A     AACTTTTACAGGAGAATTAGCCATAGGATTCATCACCAAAACCAGGGTCATGTTGAGAAT 1077
GhiFAD78-3A     AACTTTTACAGGAGAATTAGCCATAGGATTCATCACCAAAACCAGGGTCATGTTGAGAAT 876
                ************************************************************

GraFAD78-3D     GATGAATCATGGCACCCGGTTGGTTAAACTTTTCACTGATCTTTTATGGTGCTTGCTTGT 1138
GhiFAD78-3D     GATGAATCATGGCACCCGGTTGGTTAAACTTTTCACTGATCTTTTATGGTGCTTGCTTGT 936
GheFAD78-3A     GATGAATCATGGCACCCGGTTGGTTAAACTTTTCACTGAACTTTTATGGTGTTTGCTTGT 1137
GhiFAD78-3A     GATGAATCATGGCACCCGGTTGGTTAAACTTTTCACTGAACTTTTATGGTGTTTGCTTGT 936
                *************************************** *********** ********

GraFAD78-3D     TTGGTCTATTGCAACACCTTTGTTCTTCACCTAGTTTTTTGTGAATTTTATTACAGTTAA 1198
GhiFAD78-3D     TTGGTCTATTGCAACACCTTTGTTCTTCACCTAGTTTTTTGTGAATTTTATTACAGTTAA 996
GheFAD78-3A     TTGGTCTATTGCAATACCTTTGTTCTTCACCTAGTTTTTTGTGAATTTTATTACAGTTAA 1197
GhiFAD78-3A     TTGGTCTATTGCAATACCTTTGTTCTTCACCTAGTTTTTTGTGAATTTTATTACAGTTAA 996
                ************** *********************************************
                                    ----------S17F----------->
GraFAD78-3D     CTGAGAAGACATACATGAGTTTGGATAACAATGAACTAACATGGCGGTTCAAGTTTCCTT 1258
GhiFAD78-3D     CTGAGAAGACATACATGAGTTTGGATAACAATGAACTAACATGGCGGTTCAAGTTTCCTT 1056
GheFAD78-3A     CTGAGAAAATATACATGAATTTGGATAACAATGAACTAACATGGCTGTTCAAGTTTCCTT 1257
GhiFAD78-3A     CTGAGAAAATATACATGAATTTGGATAACAATGAACTAACATGGCTGTTCAAGTTTCCTT 1056
                ******* * ******** ************************** **************

GraFAD78-3D     TCCCCTTGCTTGTATATCCTTTCTACCTTGTAAGTGTTTATAGCTTGCTTGAGAAATTTG 1318
GhiFAD78-3D     TCCCCTTGCTTGTATATCCTTTCTACCTTGTAAGTGTTTATAGCTTGCTTGAGAAATTTG 1116
GheFAD78-3A     TCCCCTTGCTTGTATATCCTTTCTACCTTGTAAGTGTTTATAGCTTGCTTGAGAAATTTG 1317
GhiFAD78-3A     TCCCCTTGCTTGTATATCCTTTCTACCTTGTAAGTGTTTATAGCTTGCTTGAGAAATTTG 1116
                ************************************************************

GraFAD78-3D     ATCCTTGAGACATCCGAGTTATTACTGTCTGTTCTGTTGTTTTTAATTGCAGTGGGGAAG 1378
GhiFAD78-3D     ATCCTTGAGACATCCGAGTTATTACTGTCTGTTCTGTTGTTTTTAATTGCAGTGGGGAAG 1176
GheFAD78-3A     ATCCTTGAGACATC-GAGTTATTACTGACTGTTCTGTTGTTTTTAATTGCAGTGGGGAAG 1376
GhiFAD78-3A     ATCCTTGAGACATC-GAGTTATTACTGACTGTTCTGTTGTTTTTAATTGCAGTGGGGAAG 1175
                ************** ************ ********************************

GraFAD78-3D     AAGTCCAGGCAAGACTGGTTCTCACTTTGATCCTAACAGCGAATTGTTTGTCCCAAGTGA 1438
GhiFAD78-3D     AAGTCCAGGCAAGACTGGTTCTCACTTTGATCCTAACAGCGAATTGTTTGTCCCAAGTGA 1236
GheFAD78-3A     AAGTCCAGGCAAGACTGGTTCTCACTTTGATCCTAACAGCGAATTGTTTGTCCCAAGTGA 1436
GhiFAD78-3A     AAGTCCAGGCAAGACTGGTTCTCACTTTGATCCTAACAGCGAATTGTTTGTCCCAAGTGA 1235
                ************************************************************
```

```
GraFAD78-3D     GAGAAAAGATGTTATTACATCCAGCTTATGTTGGACAGCCATGGCTGCTATTCTTGTTGG 1498
GhiFAD78-3D     GAGAAAAGATGTTATTACATCCAGCTTATGTTGGACAGCCATGGCTGCTATTCTTGTTGG 1296
GheFAD78-3A     GAGAAAAGATGTTATTACATCCAGCTTATGTTGGATAGCCATGGCTGCTATTCTTGTTGG 1496
GhiFAD78-3A     GAGAAAAGATGTTATTACATCCAGCTTATGTTGGACAGCCATGGCTGCTATTCTTGTTGG 1295
                *********************************** ************************

GraFAD78-3D     TCTAGGCTTCACAATGGGTCCTATGCTGTTGCTTAAACTATATGGTGTTCCTTATTGGGT 1558
GhiFAD78-3D     TCTAGGCTTCACAATGGGTCCTATGCTGTTGCTTAAACTATATGGTGTTCCTTATTGGGT 1356
GheFAD78-3A     TCTAGGCTTCACAATGGGTCCTATGCTGTTGCTTAAACTATATGGTGTTCCTTATTGGGT 1556
GhiFAD78-3A     TCTAGGCTTCACAATGGGTCCTATGCTGTTGCTTAAACTATATGGTGTTCCTTATTGGGT 1355
                ************************************************************

GraFAD78-3D     AGGTTTCAACTTTCTTTTGGGATATATTAC-------GTCACCAACTATTAGTAATTTTT 1611
GhiFAD78-3D     AGGTTTCAACTTTCTTTTGGGATATATTAC-------GTCACTAACTATTAGTA-TTTTT 1408
GheFAD78-3A     AGGTTTCAACTTTCTTATGGGATATATTACACTAGTGGTCACCTACTATTAGTA-----T 1611
GhiFAD78-3A     AGGTTTCAACTTTCTTATGGGATATATTACACTAGTGGTCACCTACTATTAGTA-----T 1410
                **************** *************       *****  **********     *

GraFAD78-3D     TTTTGTTCATTTAACTATGAAAAATTATAATATAGTCACCTAAACTATTTAATTTTGTCA 1671
GhiFAD78-3D     TTTTGTTCATTTAACTATGAAAAATTATAATATAGTCACCTAAACTATTTAATTTTGTCA 1468
GheFAD78-3A     TTTTTTTCATTTAACTATGAAAAATTACAATATAATCACTTAA-TTATTTAATTTTGCCT 1670
GhiFAD78-3A     TTTTTTTCATTTAACTATGAAAAATTATAATATAGTCACTTAA-TTATTTAATTTTGCCT 1469
                **** ********************** ****** **** ***  ************ * 

GraFAD78-3D     TTTTTG-TTACCAGCTAACCAGCAATGACAGCTTTTAAAATTAGGATAATAGCAACTTTA 1730
GhiFAD78-3D     TTTTTG-TTACCAGCTAACCAGCAATGACAGCTTTTAAAATTAGGATAATAGCAACTTTA 1527
GheFAD78-3A     TTTTTGGTCACTAACTAACCAACAATGACAGCTTTTAAAATTAGGATAATAGCAACTTTA 1730
GhiFAD78-3A     TTTTTGGTCACTAACTAACCAACAATGACAGCTTTTAAAATTAGGATAATAGCAACTTTA 1529
                ****** * ** * ******* **************************************

GraFAD78-3D     ACCCTCATTTTACTCCTGTTCTAGAAAATCAAACCCTCAACATTTACACATTCTGTAATT 1790
GhiFAD78-3D     ACTCTCATTTTACTCCTGTTCTAGAAAATCAAACCCTCAACATTTACACATTCTGTAATT 1587
GheFAD78-3A     ACCCTCAATTTACTCCTGTTCTAGAAAATCAAACTCTCAACATTTACACATTCTGTAATT 1790
GhiFAD78-3A     ACCCTCAATTTACTCTTGTTCTAGAAAATTAAACTCTCAACATTTACACATTCTGTAATT 1589
                ** **** ******* ************* **** *************************

GraFAD78-3D     TGGTCTTTTTGCGGTTTTGCTATGCTTTGGGACCCCTTTCACCTAAAAAGCTAAAAAAAAG 1850
GhiFAD78-3D     TGGTCTTTTTGCGGTTTTGCTATGCTTTGGGACCCCTTTCACCTAAAAAGCTAAAAAAAAG 1647
GheFAD78-3A     TAATCTTTTTGCAGTTTTGCTTTTCTTTAGAACCCCTTTCACCTAAAAAGCTAAAAAAAA- 1849
GhiFAD78-3A     TAATCTTTTTGCAGTTTTACTTTTCTTTAGAACCCCTTTCACCTAAAAAGCTAAAAAAAA- 1648
                *  ********* ***** ** * **** * ****************************

GraFAD78-3D     TTTATTAGCTAATTTTGATTAAAAATATACAAAAAAAAAA---TAAACATAAAAAA-TCC 1906
GhiFAD78-3D     TTTATTAGCTAATTTTGATTAAAAATATACAAAAAAAA-----TAAACATAAAAAA-TCC 1701
GheFAD78-3A     -TCATTAGCTAATTTTAATTGAAAATATACAAAAAAAAAGAAGGAAACATAAAAAAATCC 1908
GhiFAD78-3A     -TCATTAGCTAATTTTAATTGAAAATATACAAAAAAAAAGAAGGAAACATAAAAAAATCC 1707
                 * ************* *** *****************      ************ ***

GraFAD78-3D     AAGATAATAATTTTCATCTTTTCTCATTCTTTTAAAATTAATCCTCAACGTCACA----- 1961
GhiFAD78-3D     AAGATAATAATTTTCATCTTTTCTCATTCTTTTAAAATTAATCCTCAACGTCACA----- 1756
GheFAD78-3A     AAGATAATAATTTTTATTTTTTTCTCATTCTTTTAAAATTAATCCTCAACGTCACACACAC 1968
GhiFAD78-3A     AAGATAATAATTTTTATTTTT-CTCATTCTTTTAAAATTAATCCTTAACGTCACACACAC 1766
                ************** ** *** *********************** *********

GraFAD78-3D     -----AAAAAA----A-CTGTAAAAGGAAGACCAAATTACACAATGTGTAAACATTGAGG 2011
GhiFAD78-3D     -----AAAAAA----AACTGTAAAAGGAAGACCAAATTACACAATGTGTAAACATTGAGG 1807
GheFAD78-3A     ACAAAAAAAAAGCACAACTGTAAAAGAAAGTCCAAATTACACAATGTGTAAATATTAAGG 2028
GhiFAD78-3A     A----AAAAAAGCACAACTGTAAAAGAAAGTCCAAATTACACATTGTGTAAATATTAAGG 1822
                     ******    * ********* *** ************ ******** *** ***

GraFAD78-3D     ATTAAATTCTTTTTGAATTAAGACTAAATTGACATAATTTATAAACATTGAGTATTAAAG 2071
GhiFAD78-3D     ATTAAATTCTTTTTGAATTAAGACTAAATTGACATAATTTATAAACATTGAGTATTAAAG 1867
GheFAD78-3A     ATTAAATTCTTTTAGAATTAAGACTAAACTGGCATAATTTATAAACATTGAGTATTAAAG 2088
GhiFAD78-3A     ATTAAATTCTTTTAGAATTAAGACTAAACTGACATAATTTATAAACATTGAGTATTAAAG 1882
                ************* ************** ** ****************************

GraFAD78-3D     TTGCTATTATGTCAATTTTACAAATTGCCACAATTAGCCAACTAGTGACAAAAAAGATAA 2131
GhiFAD78-3D     TTGCTATTATGTCAATTTTACAAATTGCCACAATTAGCCAACTAGTGACAAAAAAGATAA 1927
GheFAD78-3A     TTGCTAGTTTGTCAATTTTACAAATTGCCACAGTTAGTCAACTAGTGATAAAAAAGATAA 2148
GhiFAD78-3A     TTGCTAGTATGTCAATTTTACAAATTGCCACAGTTAGTCAACTAGTGATAAAAAAGATAA 1942
                ****** * ********************** **** ********** ***********
```

```
GraFAD78-3D     AATTGAATAATTGGATGATCGTTTTATAACTTTTTATAATTAAATGACCTAAAAAGAAAT 2191
GhiFAD78-3D     AATTGAATAATTGGATGATCGTTTTGTAACTTTTCATAATTAAATGACCTAAAAAGAAAT 1987
GheFAD78-3A     AATTAAATAATTAGATGATCGTT--CTAACTTTTCATAATTAGATGATTAAAAAAGAAAT 2206
GhiFAD78-3A     AATTGAATAATTAGATGATCGTT--GTAACTTTTCATAATTAGATGATTAAAAAAGAAAT 2000
                **** ******* **********   ******** ******* ****   **********

GraFAD78-3D     TTACTAATAGATGAATAATTACTTGTATAGTTTACCCTTCTTTTGGTCATACATATGTAT 2251
GhiFAD78-3D     TTACTAATAGATGAATAATTACTTGTATAGTTTACCCTTCTTTTGGTCATACATATGTAT 2047
GheFAD78-3A     TTATTAATAGATGAGTAATTACTTGTATAGTTTACCCTTCTTTTGGTCATACATATGTCT 2266
GhiFAD78-3A     TTACTAATAGATGAGTAATTACTTGTATAGTTTACCCTTCTTTTGGTCATACATATGTCT 2060
                *** ********** ******************************************* *
                                                                          <-
GraFAD78-3D     TTACCAGTTATAGAATACAGCTTGACTTTGTTTTCAAGTTTAGATATTCGTAATTTGGTT 2311
GhiFAD78-3D     TTACCAGTTATAGAATACAGCTTGACTTTGTTTTCAAGTTTAGATATTCGTAATTTGGTT 2107
GheFAD78-3A     TTACCAGTTATAGAATACAGCTTGACTTTGTTTTCAAGTTTAGATATTCGTAATTTGGCT 2326
GhiFAD78-3A     TTACCAGTTATAGAATACAGCTTGACTTTGTTTTCAAGTTTAGATATTCGTAATTTGGCT 2120
                ********************************************************** *
                --------S23R------------
GraFAD78-3D     GGATTTTGTAACATACATGCATCATCATGGTCATCAAGACAAACTTCCTTGGTACCGTAG 2371
GhiFAD78-3D     GGATTTTGTAACATACATGCATCATCATGGTCATCAAGACAAACTTCCTTGGTACCGTGG 2167
GheFAD78-3A     GGATTTTGTAACATACATGCATCATCATGGTCATCAAGACAAACTTCCTTGGTACCGTGG 2386
GhiFAD78-3A     GGATTTTGTAACATACATGCATCATCATGGTCATCAAGACAAACTTCCTTGGTACCGTGG 2180
                ********************************************************** *

GraFAD78-3D     GAAGGTATGATTCTCTTACCTTGGTCTAATATGTTCTGTCTGTGAGAAGACATCTTTTTG 2431
GhiFAD78-3D     GAAGGTATAATTCTCTTACCTTGGTCTAATGTGTTCTGTCTGTGAGAAGACATCTTTTTG 2227
GheFAD78-3A     GAAGGTATGATTCTCTTACCTTGATCTAATGTGCTCTGTCTGTGAGGAGACATCTTTTTG 2446
GhiFAD78-3A     GAAGGTATGATTCTCTTACCTTGGTCTAATGTGTTCTGTCTGTGAGGAAACATCTTTTTG 2240
                ******** ************** ****** ** ************ * ***********

GraFAD78-3D     GTTTATTTTGAACTTGACATTACAACATTCTGCAGGAATGGAGTTACTTAAGGGGAGGGC 2491
GhiFAD78-3D     GTTTATTTTGAACTTGACATTACAACATTCTGCAGGAATGGAGTTACATAAGGGGAGGGC 2287
GheFAD78-3A     GTTTATTTTGAGCTTGACATTACAACATTCTGCAGGAATGGAGTTACTTAAGGGGAGGGC 2506
GhiFAD78-3A     GTTTATTTTGAGCTTGACATTACAACATTCTGCAGGAATGGAGTTACTTAAGGGGAGGGC 2300
                *********** *********************************** ************

GraFAD78-3D     TTACAACACTTGACCGTGATTATGGATGGATGAACAACATTCATCATGATATTGGAACCC 2551
GhiFAD78-3D     TTACAACACTTGACCGTGATTATGGATGGATGAACAACATTCATCATGATATTGGAACCC 2347
GheFAD78-3A     TTACAACACTTGACCGTGATTATGGATGGATGAACAACATTCATCATGATATTGGAACCC 2566
GhiFAD78-3A     TTACAACACTTGACCGTGATTATGGATGGATGAACAACATTCATCATGATATTGGAACCC 2360
                ************************************************************

GraFAD78-3D     ATGTTATACACCATCTCTTTCCTCAGATCCCTCATTACCACTTAATAGAGGCTGTGAGTT 2611
GhiFAD78-3D     ATGTCATACACCATCTCTTTCCTCAGATCCCTCATTACCACTTAATAGAGGCTGTGAGTT 2407
GheFAD78-3A     ATGTCATACACCATCTCTTTCCTCAGATCCCTCATTACCGCCTAATAGAGGCTGTGAGTT 2626
GhiFAD78-3A     ATGTCATACACCATCTCTTTCCTCAGATCCCTCATTACCGCCTAATAGAGGCTGTGAGTT 2420
                **** ********************************** * ******************

GraFAD78-3D     CATCTCTTTAGTAGTTTTGGCTTGGGTAAAGATACTATGGAAGTCCCCGTATTAGGATTT 2671
GhiFAD78-3D     CATCTCTTTAGTAGTTTTGGCTTGGGTAAAGATACTATGGAAGTCCCCGTATTAGGATTT 2467
GheFAD78-3A     CATCTCTTTAGTAGTTTTGGCTTGGGTAGAAATACCATGGAAGTCCCTATACTAGGATTT 2686
GhiFAD78-3A     CATCTCTTTAGTAGTTTTGGCTTGGGTAGAAATACCATGGAAGTTCCTATATTAGGATTT 2480
                **************************** * **** ******** **  ** ********

GraFAD78-3D     AGATTGCATTTTGCCTTATTTACTCAAAAAATTGAATAAACTAGTCCCTTACGTTAAATC 2731
GhiFAD78-3D     AAATTGCATTTTGTCTTATTTACTCAAAAAATTGAATAAACTAGTCCCTTACGTTAAATC 2527
GheFAD78-3A     AGATTGCATTTTACTTTAATTACTCAAAAAATTGAATAAACTAGTTCGTTACGTTAAATC 2746
GhiFAD78-3A     AGATTGCATTTTACCTTAATTACTCAAAAAATTGAATAAACTAGTTCGTTACGTTAAATC 2540
                * **********   *** ************************** * ************

GraFAD78-3D     AAAGAGAAAATTAGTCCTTTCAGTTAAAATTTCCATCTATTTCTATTGTTAAA-TTGGCA 2790
GhiFAD78-3D     AAAGAGAAAATTAGTCATTTCAGTTAAAATTTCCATCTATTTCTATTGTTAAAATTGGCA 2587
GheFAD78-3A     AAAGAGCAAATTAGTCCTTTCAGTTAAAATTTTCATCTATTTTTACGGTTAAAATTGGCA 2806
GhiFAD78-3A     AAAGAGCAAATTAGTCTTTTCAGTTAAAACTTCCATCTATTTCTACTGTTAAAATTGACA 2600
                ****** ********* ************ ** ********* **  ****** *** **

GraFAD78-3D     TGACTGATAGAATAACCAGATAGTTACATATGATACGTCACGTGTACCTCATGTTAACAT 2850
GhiFAD78-3D     TGACTGATAGAATAACCAGATAGTTACATATGATACGCCACGTGTACCTCATGTTAATAT 2647
GheFAD78-3A     TGACTAATAGAATAACTATACAGTTACATATGATATGCCACGTATACCTCATGTTAATGT 2866
GhiFAD78-3A     TGACTAATAGAATAACTAGACAGTTACATATGATATGTCAAGTATACCTCATGTTAATGT 2660
                ***** ********** * * ************** * ** ** *************  *
```

```
GraFAD78-3D      ATAGAGATGGATGAAATTT-----------TTAATACGAGGATCATTTTGCTCTTTACTC 2899
GhiFAD78-3D      ATAGAGATGGATGAAATTT-----------TTAATACGAGGACCACTTTGCTCTTTACTC 2696
GheFAD78-3A      ATAAAGATGGATGAAATTT-----------TTAATATGAGGACCACTTTGCTCTTTACTC 2915
GhiFAD78-3A      ATAAAGATGGACGAAATTTGGACGAAATTTTTAATTTGAGGACCACTTTGCTCTTTACTC 2720
                 *** ******* *******           *****  ***** ** **************

GraFAD78-3D      TAACATGTAGGTATTAATTTACTCATTTTTTAGTAAAGGGGCAAAATGTAATTTAACTTC 2959
GhiFAD78-3D      TAACATGTAGGTATTAATTTACTCGTTTTTTAGTAAATGGGCAAAATGTAATTTAACTTC 2756
GheFAD78-3A      TAATATTTAGGTATTAATTTGCTTATTTTTTAGTAAATAGGTAAAATGTAATCTAACTTT 2975
GhiFAD78-3A      TAATATGTAGGCATTAATTTGCTTATTTTTTAGTAAATAGGTAAAATGTAATCTAACTTT 2780
                 *** ** **** ******** **  ************  ** ********** ******

GraFAD78-3D      TAATACAAAGTCCTCCGTGGTAGTTTTACTTTTTGGCTTGTGATATGCTCTTTTTTCTAT 3019
GhiFAD78-3D      TAATACAAAGTC-TCCGTGGTAGTTTTACTTTTTGGCTTGTGATATGCTCTTTTTTCTAT 2815
GheFAD78-3A      TAATACAAAGGCATCCATGGTAGTTTTACTTTTTGG--TGTGATATGCTCTTTTTTCTAT 3033
GhiFAD78-3A      TAATACAAAGGCATCCATGGTAGTTTTACTTTTTGG--TGTGATATGCTCTTTTTTCTAT 2838
                 ********** * *** *******************  **********************

GraFAD78-3D      TTATTTTATGTGTCCTAATGTTTTGGTCTCATAGACTAAGGCAGCTAAGCCGGTTCTTGG 3079
GhiFAD78-3D      TTATTTTATGTGTCCTAATGTTTTGGTCTCATAGACTAAGGCAGCTAAGCCGGTTCTTGG 2875
GheFAD78-3A      TTATTTTATGTGTCCTAATGTTTTAGTCTCATAGACTAAGGCAGCTAAGCCGGTTCTTGG 3093
GhiFAD78-3A      TTATTTTATGTGTCCTAATGTTTTAGTCTCATAGACTAAGGCAGCTAAGCCGGTTCTTGG 2898
                 ************************ ***********************************
                                                <---------S18R-----------
GraFAD78-3D      AAAATACTATCGGGAGCCAGAACGATCAGGACCTCTACCTTTTTACCTCATCGGGGTTTT 3139
GhiFAD78-3D      AAAATACTATCGGGAGCCAGAACGATCAGGACCTATACCTTTTTACCTCATCGGAGTTTT 2935
GheFAD78-3A      AAAATACTATCGGGAGCCAGAACGATCGGGACCTCTACCTTTTCACCTCATCGGAGTTTT 3153
GhiFAD78-3A      AAAATACTATCGGGAGCCAGAACGATCGAGACCTCTACCTTTTCACCTCATCGGAGTTTT 2958
                 ***************************  ***** ******** ********** *****

GraFAD78-3D      TATAAGAAGCTTGAAGGAAGATCACTACGTTAGTGACACTGGCGATGTTGTCTACTAC-A 3198
GhiFAD78-3D      TATAAGAAGCTTGAAGGAAGATCACTACGTTAGTGACACCGGCGATGTTGTCTACTAC-A 2994
GheFAD78-3A      TATAAGAAGCTTGAAGGAAGATCACTACGTTAGTGACACCGGCGATGTTGTCTACTACCA 3213
GhiFAD78-3A      TATAAGAAGCTTGAAGGAAGATCACTACGTTAGTGACACCGGCGATGTTGTCTACTACCA 3018
                 *************************************** ****************** *

GraFAD78-3D      AAACTGACCCAAACATTTTCAAGTCAGCATGATGCTTCATAAAACTTCAAGCTTTTTGAA      3258
GhiFAD78-3D      AAACTGACCCAAACATTTTCAAGTCAGCATGAGCATGCTAA------------------- C25R 3035
GheFAD78-3A      AAACTGACCCAAACATTTTCAAGTCAGCATGATGCTTCATAAAACTTCAA---------- C23R 3263
GhiFAD78-3A      AAACTGACCCAAACATTTTCAAGTCAGCATGAGCATGCTAA------------------- C25R 3059
                 ********************************

GraFAD78-3D      CCGGACTCGGGTGCTTAACCATAAGAGATCTAAGCACTGAACTTTGTATGGCCAT      C22R 3313
GhiFAD78-3D      -------------------------------------------------------
GheFAD78-3A      -------------------------------------------------------
GhiFAD78-3A      -------------------------------------------------------
```
